# Developing Photoactive Coumarin-Caged *N*-Hydroxysulfonamides for Generation of Nitroxyl (HNO)

**DOI:** 10.3390/molecules29163918

**Published:** 2024-08-19

**Authors:** Mohammad S. Rahman, Vinay Bharadwaj, Anau K. H. S. Lautaha, Paul Sampson, Nicola E. Brasch, Alexander J. Seed

**Affiliations:** 1 Department of Chemistry and Biochemistry, Kent State University, Kent, OH 44242, USA; 2 School of Science, Auckland University of Technology, Private Bag 92006, Auckland 1142, New Zealand; 3 The Dodd-Walls Centre for Quantum and Photonic Technologies, Dunedin 9054, New Zealand; 4 The Maurice Wilkins Centre for Molecular Biodiscovery, Private Bag 92019, Auckland 1142, New Zealand

**Keywords:** nitroxyl, HNO, photochemistry, photocage, *N*-hydroxysulfonamide, Piloty’s acid

## Abstract

Photoactive *N*-hydroxysulfonamides photocaged with the (6-bromo-7-hydroxycoumarin-4-yl)methyl chromophore have been successfully synthesized, and the mechanisms of photodecomposition investigated for two of the compounds. Upon irradiation up to 97% of a diagnostic marker for (H)NO release, sulfinate was observed for the trifluoromethanesulfonamide system. In the absence of a species that reacts rapidly with (H)NO, (H)NO instead reacts with the carbocation intermediate to ultimately generate (*E*)-BHC-oxime and (*Z*)-BHC-oxime. Alternatively, the carbocation intermediate reacts with solvent water to give a diol. Deprotonation of the N(H) proton is required for HNO generation via concerted C-O/N-S bond cleavage, whereas the protonation state of the O(H) does not affect the observed photoproducts. If the N(H) is protonated, C-O bond cleavage to generate the parent *N*-hydroxysulfonamide will occur, and/or O-N bond cleavage to generate a sulfonamide. The undesired competing O-N bond cleavage pathway increases when the volume percentage of water in acetonitrile/water solvent mixtures is increased.

## 1. Introduction

Nitroxyl (HNO) is now recognized to be an important biological molecule, due to its unique chemical reactivity and significant promise in medicine [1,2,3,4,5,6]. HNO shows distinctly different physiological and pharmacological properties from the well-studied nitric oxide [6,7,8]. HNO is a vasodilator and improves myocardial contractility, two vital features that render HNO a promising therapeutic agent for the treatment of acute congestive heart failure [4,5,9,10,11]. Furthermore, the HNO-generating compounds CXL-1020 and CXL-1427 (Cimlanod) have shown improved cardiovascular performance in failing hearts [4,5,9,11,12,13]. HNO is likely to be generated endogenously via a range of reactions. However, rapid dimerization of HNO in solution complicates studies of HNO’s fundamental chemical and biological reactivity. Molecules that release HNO in situ (HNO donors) are therefore an integral part of investigations on the reactivity of this emerging biological signaling molecule.

A range of HNO-releasing compounds have been developed, where the release of HNO at physiological pH conditions is typically on the minutes to hours timescale, although the half-life is less than a minute in rare cases [14,15]. Since HNO reacts rapidly with biomolecules, including reacting with thiols [16], metalloproteins [5] and oxidants [17], this has inspired us and others to investigate the potential of light for rapid and spatiotemporally controlled release of HNO from photoactive molecules. Photoactive HNO donor molecules are valuable chemical tools for in situ investigations of the physiological and pharmacological roles of the unstable and highly reactive HNO in biological systems. These molecules could also be used for in vitro studies to directly obtain kinetic information on the rapid rates of HNO’s reactions with biomolecules. Nakagawa et al. investigated the photochemical generation of HNO from hetero-Diels–Alder cycloadducts [18,19,20]. This group also recently reported the synthesis of (7-diethylaminocoumarin-4-yl)methyl photocaged derivatives of the Piloty’s acid derivatives (2-Br)PhSO_2_NHOH and (2-NO_2_)PhSO_2_NHOH [21]. It was suggested that the HNO-generating pathway proceeds via C-O bond cleavage although detailed studies on the mechanism of photodecomposition were not carried out. Our group has also investigated the potential of photocaged *N*-hydroxysulfonamides to generate HNO, using *O*-(3-hydroxynaphthalen-2-yl)methyl (3,2-HNM) [22,23], *O*-(6-hydroxynaphthalen-2-yl)methyl (6,2-HNM) [24], *O*-2-nitrobenzyl (2-NB) [25], and 2-(2-nitrophenyl)ethyl phototriggers [26]. Interestingly, whether or not the HNO-generating pathway dominates is highly dependent on the chromophore, sulfohydroxamate and solvent. For 6,2-HNM-caged *N*-hydroxysulfonamides, up to 98% HNO was observed in acetonitrile/aqueous phosphate buffer (pH 7.0) solvent mixtures.

Based on our recent success with the (hydroxynapthalen-2-yl)methyl systems, in this study, a family of photoactivatable (6-bromo-7-hydroxycoumarin-4-yl)methyl-caged *N*-hydroxysulfonamides (BHCM-ONHSO_2_R, R = CF_3_ (**8a**), CH_3_ (**8b**) and (2-SO_2_Me)Ph (**8c**)) were targeted. The rationale for choosing the (6-bromo-7-hydroxycoumarin-4-yl)methyl (BHCM) chromophore is that, unlike with our previously studied systems, visible light can be used to trigger photodegradation. Furthermore, rapid heterolytic bond cleavage of the excited parent molecule is expected (~10^9^ s^−1^) [27] to generate (H)NO and a carbocation intermediate. The BHCM chromophore also exhibits good aqueous solubility, in addition to a strong absorption extending into the visible range [28,29,30,31].

## 2. Results

**Scheme 1:**
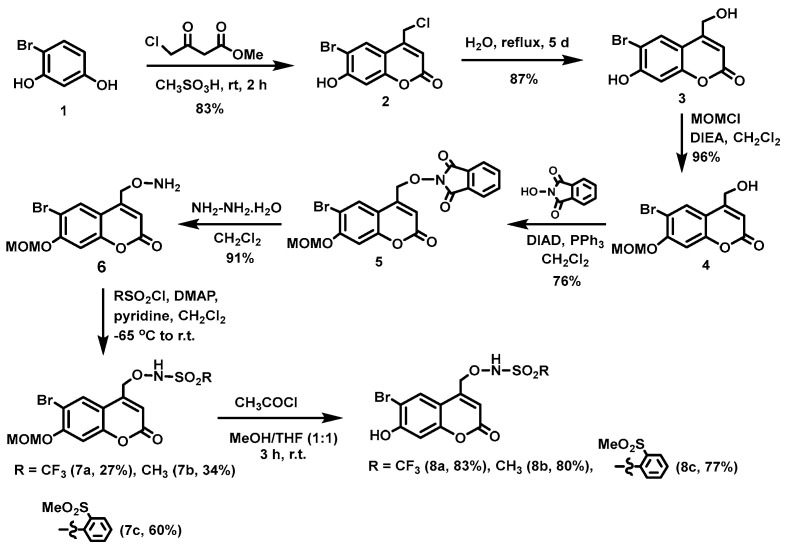
Synthesis of BHCM-caged *N*-hydroxysulfonamides **8a**–**c**.

(6-Bromo-7-hydroxycoumarin-4-yl)methyl-caged *N*-hydroxysulfonamide HNO donors **8a**–**c** were prepared as shown in Scheme 1. The diol **3** was generated by Pechmann condensation of 4-bromoresorcinol (**1**) with methyl 4-chloroacetoacetate in the presence of CH_3_SO_3_H, followed by hydrolysis of **2** using water at reflux for 5 days [32]. Chemoselective protection of the phenol with methoxymethyl chloride (MOMCl) generated MOM-protected alcohol **4** [32], which was then coupled with *N*-hydroxyphthalimide under Mitsunobu conditions to afford **5** [22]. Transamidation of **5** with NH_2_NH_2_.H_2_O generated alkoxyamine **6**. Then, sulfonation of **6** was accomplished by treating with a series of sulfonylating agents RSO_2_Cl (R = CF_3_, CH_3_, ((2-SO_2_Me)Ph) to obtain MOM-protected adducts **7**. Finally, deprotection of the MOM group under mild acidic conditions (methanolic HCl generated in situ from the reaction of AcCl and MeOH [33]) afforded target compounds **8a**–**c** in 11–22% overall yield over 8 steps.

The thermal stabilities of **8a** and **8b** were assessed in anaerobic CD_3_CN in the absence of light, by ^19^F and/or ^1^H NMR spectroscopy. No detectable decomposition was observed after ~10 min for both **8a** and **8b**. After 8 days, **8a** had undergone 10% decomposition to give CF_3_SO_2_NH_2_ (^19^F NMR spectroscopy), whereas **8b** was stable over this time period.

The UV–vis spectra of **8a** and **8b** are shown in Section S1 in the Supplementary Information. The wavelength maxima of **8a** and **8b** (370 and 366 nm, respectively) can be assigned to a π–π* transition [34]. These transitions are lower in energy compared to 7-hydroxycoumarin (λ_max_ = 350 nm [35]) due to the presence of the 6-bromo substituent [36]. The molar extinction coefficients for **8a** and **8b** are 2.50 × 10^4^ (370 nm) and 1.42 × 10^4^ (366 nm) M^−1^ cm^−1^, respectively.

**8a** and **8b** absorb significantly in the visible region. The photostability of **8a** in aerobic CD_3_CN under typical lab light conditions was therefore investigated. After 12 h, 58% **8a** had decomposed to give CF_3_SO_2_NH_2_ (33%), CF_3_SO_2_^−^ (17%) and CF_3_SO_3_^−^ (8%) (^19^F NMR), Section S2, SI. **8a** was found to be stable in the solid form under the same light conditions. The photostability of **8b** in CD_3_CN was also examined under typical lab light conditions, with no detectable photodecomposition after 12 h (^1^H NMR spectroscopy). Both **8a** (see Section S3, SI) and **8b** were found to be stable under red light conditions for at least 4 h in CD_3_CN. Therefore, all the photolysis experiments were carried out under red light, including sample preparation.

### 2.1. Characterization of the Photoproducts in CD_3_CN/Phosphate Buffer Mixtures

**Scheme 2:**
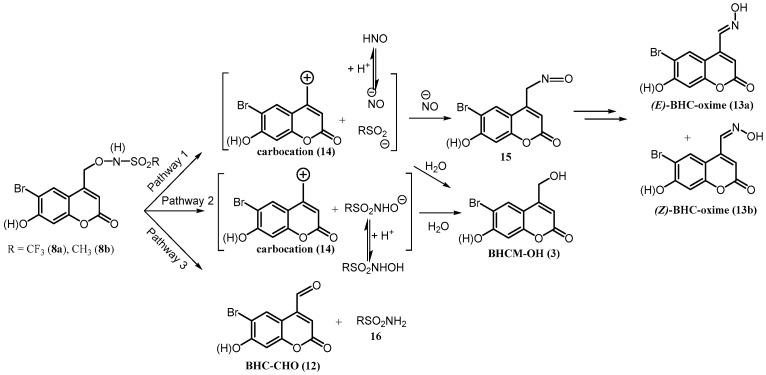
Proposed pathways for the photodecomposition of **8a** and **8b**.

It is well established that (coumarin-4-yl)methyl-caged derivatives incorporating good leaving groups on the benzylic carbon undergo photolysis to eliminate the leaving group and generate a solvent-caged (coumarin-4-yl)methyl carbocation intermediate [28,30,32,37,38,39,40]. Previous studies of photocaged *N*-hydroxysulfonamides have shown that heterolytic O-N bond cleavage can also occur in addition to C-O and concomitant C-O/N-S bond cleavage [22,23]. Possible pathways for photodecomposition of **8a** and **8b** are presented in Scheme 2. In Pathway 1, concerted C-O/N-S bond cleavage occurs to generate a solvent-caged reactive carbocation intermediate **14**, NO^−^ and the corresponding sulfinate. Previously, (*E*)-6-hydroxynaphthalene-2-aldoxime ((*E*)-6,2-HNM-oxime) was observed as a photoproduct upon the irradiation of (6-hydroxynaphthalen-2-yl)methyl (6,2-HNM)-caged trifluoromethanesulfonyl hydroxamic acid, from the reaction of the carbocation intermediate with (H)NO [24]. We speculate here that analogous rapid trapping of the carbocation intermediate **14** by the nucleophilic (H)NO in the solvent cage leads to the formation of nitroso intermediate **15**, as shown in Scheme 2, before diffusion of (H)NO from the solvent cage can occur. This species then tautomerizes to (*E*)-BHC-oxime **13a** which photoisomerizes to give (*Z*)-BHC-oxime **13b**. The reaction of the carbocation intermediate **14** with solvent H_2_O would instead generate 4-hydroxymethylcoumarin diol **3**, BHCM-OH [28,30,32,37,38,39,40]. Competing C-O bond cleavage would generate the same carbocation intermediate and the parent anion of the *N*-hydroxysulfonamide, RSO_2_NHO^−^ (R = CF_3_ or CH_3_), Pathway 2. Again, the carbocation could react with H_2_O to generate **3**. O-N bond cleavage is also possible, with the generation of the corresponding sulfonamide **16** and (6-bromo-7-hydroxycoumarin-4-yl)methyl aldehyde (BHC-CHO) (**12**), Pathway 3.

The effect of the solvent composition (CD_3_CN and phosphate buffer (0.10 M, pH 7.0)) on the photoproducts obtained upon irradiation of **8a** was investigated under anaerobic conditions. The samples were irradiated using a Rayonet photoreactor (350 nm bulbs). The percentage of the fluorinated aliphatic photoproducts was established by integrating the peaks in the ^19^F NMR spectrum of the photoproducts upon complete photodecomposition of **8a**. The release of one equivalent of RSO_2_^−^ (R = CF_3_ for **8a**) for each (H)NO generated, Pathway 1 Scheme 2, is a convenient marker for determining the percentage of the HNO-generating pathway for photocaged *N*-hydroxysulfonamides by NMR spectroscopy [23]. The aromatic photoproducts were characterized using ^1^H NMR spectroscopy.

**Figure 1:**
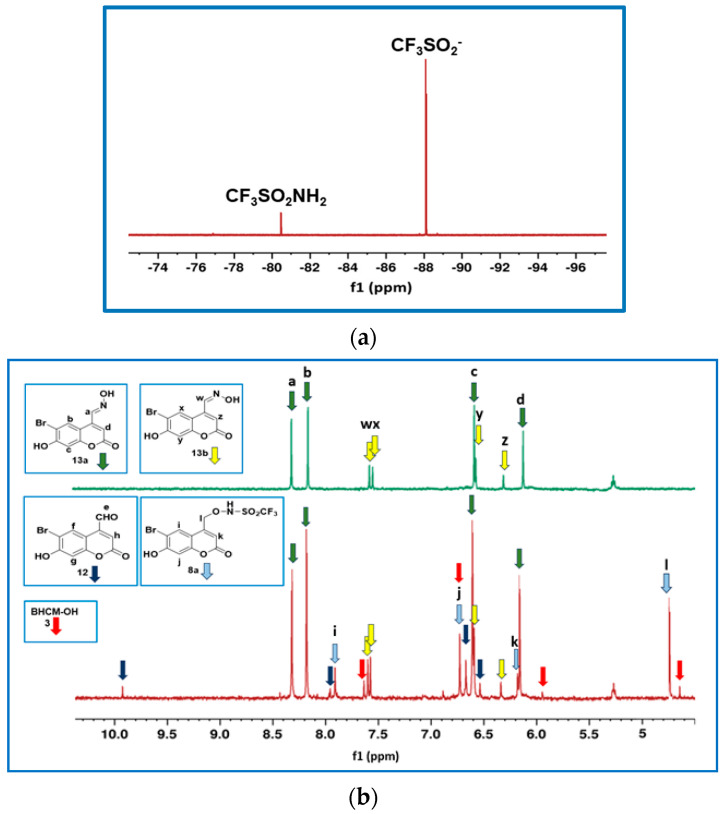
(**a**) ^19^F NMR spectrum of **8a** (1.0 mM) in 80:20 *v*/*v* CD_3_CN:phosphate buffer (pH 7.0, 0.1 M) after complete photodecomposition (irradiation for 5.30 min). A Rayonet photoreactor (eight 4 W lamps, 350 nm) was used as the light source. The estimated error in the percentage photoproducts is 2%. (**b**) Comparison of the ^1^H NMR spectrum of a partially irradiated sample (60 s duration) of **8a** (1.0 mM) in 80:20 *v*/*v* CD_3_CN:phosphate buffer (pH 7.0, 0.1 M) with (*E*)-BHC oxime (**13a**) and (*Z*)-BHC oxime (**13b**). Small peaks attributable to BHCM-OH (**3**) are observed at 7.65 and 6.72, with the remaining two peaks (6.22, 4.66 ppm) overlapping with the peaks of other compounds. The minor peaks at 6.88 ppm and 8.43 ppm could not be assigned. The peak at 5.27 ppm is an impurity in the CD_3_CN solvent.

Figure 1a shows the ^19^F NMR spectrum obtained upon completely photolyzing **8a** in anaerobic 80:20 *v*/*v* CD_3_CN:phosphate buffer (pH 7.0, 0.1 M). Upon photolysis, **8a** released CF_3_SO_2_^−^ (94%, −88.1 ppm); that is, almost stoichiometric release of (H)NO. In addition, a small amount of CF_3_SO_2_NH_2_ (6%, −80.5 ppm) was generated via competing photoinduced O-N cleavage of **8a** (Scheme 2, Pathway 3). The chemical shifts of both fluorinated photoproducts were confirmed using authentic samples of these compounds. No formation and subsequent decomposition of CF_3_SO_2_N(H)O(H) was observed, in line with results found on related systems [22,23,24]. If CF_3_SO_2_N(H)O(H) was an intermediate, we would have expected to observe it in the ^19^F NMR spectra during data collection since the half-life (t_1/2_) for decomposition of this compound is ~13 min in pH 7.4 buffer solution [41]. Hence, CF_3_SO_2_^−^ can be presumed to form exclusively via Pathway 1, Scheme 2.

The aromatic photoproducts of **8a** were characterized after ~90% photodecomposition using ^1^H NMR spectroscopy. Figure 1b shows the ^1^H NMR spectrum of a partially irradiated sample (1.0 min) of **8a**. The ^1^H NMR spectra of authentic samples of BHC-CHO (**12**), BHCM-OH (**3**), (*E*)-BHC-oxime (**13a**), and an irradiated sample of (*E*)-BHC-oxime (**13a**) (which isomerizes to give (*Z*)-BHC-oxime (**13b**), Section S4, SI) were also recorded for comparison purposes. The chemical shifts of the main photoproduct at 8.32, 8.17, 6.62 and 6.16 ppm can be assigned to (*E*)-BHC-oxime (**13a**), with the smaller peaks at 7.59, 7.56, 6.60 and 6.34 ppm attributable to its isomer (*Z*)-BHC-oxime (**13b**). Small ^1^H NMR peaks from BHC-CHO (**12**) and BHCM-OH (**3**) are observed at 9.93, 7.97, 6.68 and 6.54 ppm, and 7.65, 6.72, 6.22 and 4.66, respectively. Both BHC-CHO (**12**) and BHCM-OH (**3**) were found to be photostable. Small unknown peaks from secondary photoproducts (6.88, and 8.43 ppm) were also observed.

**Figure 2:**
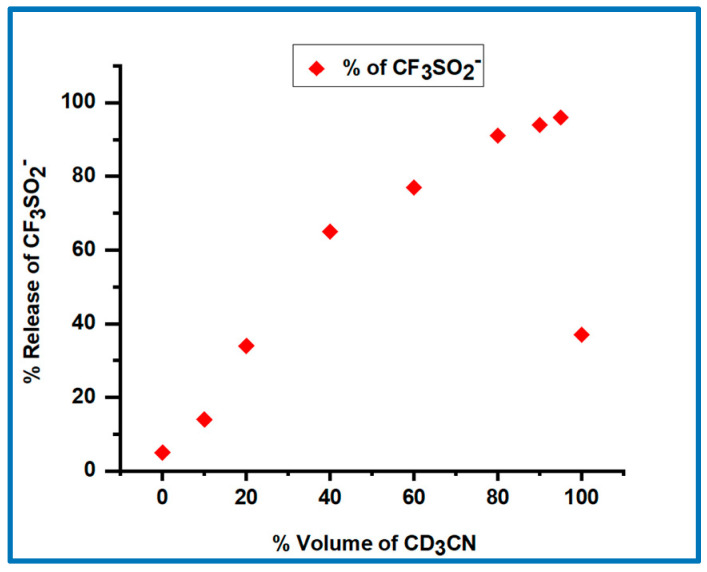
Percentage of CF_3_SO_2_^−^ released versus the % volume of CD_3_CN for the photolysis of **8a** under anaerobic conditions in CD_3_CN/phosphate buffer (0.10 M, pH or pD 7.0) solvent mixtures. The estimated error in each value is 2–3%.

The effect of the solvent composition on the photoproducts obtained from **8a** is shown in Figure 2. The percentage of the desired pathway (Pathway 1, Scheme 2) increases as the volume percentage of CD_3_CN in the CD_3_CN/phosphate buffer (0.10 M, pH 7.0) increases, with 97% CF_3_SO_2_^−^ (and (H)NO) generated in 95:5 *v*/*v* CD_3_CN:phosphate buffer. However, in pure CD_3_CN only ~36% CF_3_SO_2_^−^ is produced. A dramatic decrease in the percentage of the (H)NO-generating pathway in pure CD_3_CN compared to solvent mixtures of CD_3_CN and phosphate buffer (pH 7.0) was also observed for the (6-hydroxylnapthylen-2-yl)methyl-ON(H)SO_2_CF_3_ system, with only O-N bond cleavage observed in pure CD_3_CN [23,24].

The photoproducts obtained upon the irradiation of **8b** were also determined as a function of solvent composition in phosphate buffer (30 mM, pH 7.0)/CD_3_CN solvent mixtures. **8b** solutions were irradiated using the Rayonet photoreactor (350 nm). The photoproducts were characterized by recording the ^1^H NMR spectra of fully and partially photodecomposed samples. Products were assigned by recording the ^1^H NMR spectra of authentic samples in the same solvent mixture.

**Figure 3:**
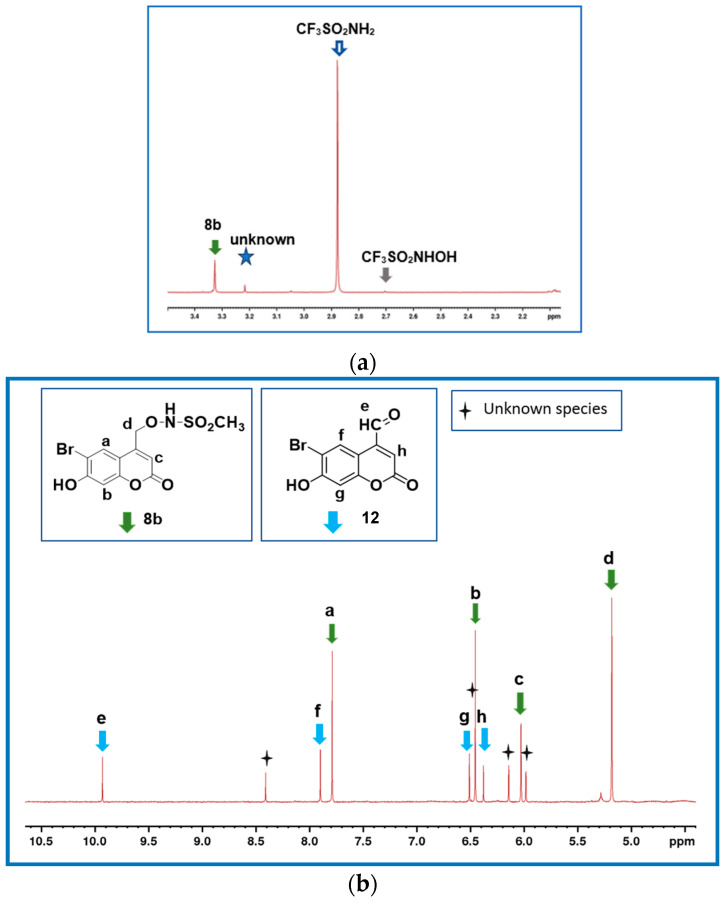
(**a**) ^1^H NMR spectrum (2.1–3.5 ppm region) upon complete photodecomposition of **8b** (1.0 mM) recorded after 5 min irradiation in a 60:40 *v*/*v* mixture of CD_3_CN and phosphate buffer (30 mM, pH 7.0). The small peak at 3.21 ppm could not be assigned. (**b**) ^1^H NMR spectrum (4.5–10.5 ppm region) of **8b** (1.0 mM) after partial photodecomposition (16 s) in a 60:40 *v*/*v* mixture of CD_3_CN:phosphate buffer (30 mM, pH 7.0). The small peak at 5.37 ppm is an impurity in the CD_3_CN solvent. The peaks at 8.41, 6.46, 6.14 and 5.98 ppm could not be assigned.

Figure 3a shows the ^1^H NMR spectrum of a completely photodecomposed sample of **8b** in the 2–5.5 ppm region in 60:40 *v*/*v*, CD_3_CN to 30 mM phosphate buffer, pH 7.0. CH_3_SO_2_NH_2_ was observed, consistent with O-N bond cleavage occurring. A very small amount of an unknown species was also observed at 3.21 ppm, and a trace of CH_3_SO_2_NHOH. Figure 3b shows the ^1^H NMR spectrum of a partially irradiated (16 s) sample of **8b**. The peaks at 9.93, 7.90, 6.51 and 6.38 ppm can be assigned to BHC-CHO (**10**) by comparison with an authentic sample of **12**. BHC-CHO was found to be photostable under the irradiation conditions. There was no evidence for (*E*)-BHC-oxime (**13a**; 8.30, 8.27, 6.56 and 6.18 ppm), (*Z*)-BHC-oxime (**13b**; 7.57, 7.67, 6.54 and 6.37 ppm) or BHCM-OH (**3**; 7.62, 6.51, 6.08 and 4.67 ppm) in the photoproduct mixture, consistent with essentially only O-N bond cleavage occurring. Peaks which could not be assigned were observed at 8.41 (1H, s), 6.46 (1H, s), 6.14 (2H, s) and 5.98 (2H, s) ppm, and a major peak with *m*/*z* values consistent with the parent molecule being C_10_H_5_BrO_5_ was observed by LC-MS. Further experiments were not undertaken to fully characterize this species since it is a photoproduct(s) of the undesired O-N bond cleavage pathway.

Interestingly, varying the solvent composition from pure CD_3_CN to 90:10 *v*/*v* phosphate buffer: CD_3_CN does not significantly affect the observed photoproducts (see Table 1). In each case, CH_3_SO_2_NH_2_ (79–93%, O-N bond cleavage) was observed as the major product; small amounts of CH_3_SO_2_NHOH (1–8%), CH_3_SO_2_^−^ (0–3%), CH_3_SO_3_^−^ (0–9%) and an unknown species were also observed.

### 2.2. Evidence for HNO Generation Using a Phosphine Trap

Triphenylphosphine derivatives have been widely used to confirm that HNO is released from HNO donor molecules [42,43,44,45,46,47,48,49,50]. An anaerobic solution of **8a** (1.0 mM) and the established phosphine trap **S1** (see Figure S6, SI) (2.0 mM) in 80:20 *v*/*v* CD_3_CN:phosphate buffer (5.0 mM, pH 7.0) was irradiated at 350 nm using a Rayonet photoreactor. **8a** decomposed (^19^F NMR spectroscopy) to give CF_3_SO_2_^−^ (89%, −88.2 ppm) and CF_3_SO_2_NH_2_ (11%, −80.5 ppm). In the ^31^P NMR spectrum, the corresponding phosphine aza-ylide and phosphine amide were observed, as expected if HNO is released upon irradiation of **8a**. These experiments offer convincing qualitative evidence for HNO release on photolysis of **8a**. Further details are given in Section S5, SI.

### 2.3. Effect of O_2_

The aerobic photolysis of **8a** was performed, to determine the effect of O_2_ on photodecomposition. Since photodecomposition of **8a** and release of HNO is predicted to occur via the singlet excited state, this would suggest that the impact of O_2_ (a triplet species in the ground state) on the observed photoproducts should be minimal. Like anaerobic photolysis, aerobic photolysis of **8a** predominantly occurred via C-O/N-S bond cleavage, generating 94% CF_3_SO_2_^−^. Surprisingly, however, aerobic photolysis of **8a** resulted in the generation of larger amounts of diol **3** (a secondary diagnostic marker of HNO release) (30% vs. 4% observed under anaerobic conditions), implying enhanced release of (H)NO from the solvent cage under aerobic conditions. Direct quantification of HNO released in the presence of air was not possible using HNO trapping agents like aquacobalamin (H_2_OCbl(III)) [23] or a phosphine, due to instability of the diagnostic products and interference from secondary reactions of these traps with O_2_. The release of larger amounts of diol **3** under aerobic conditions would be consistent with competing oxidation of released (H)NO to NO by air, prior to any trapping with the incipient carbocation to afford an oxime byproduct. NO is unlikely to react rapidly with this carbocation intermediate, leading to increased trapping of the carbocation by solvent to form diol **3** [51].

### 2.4. Determination of the pKa Values for ***8a*** and ***8b***

To understand the role of the protonation state of the ground state molecule on the mechanism of photodecomposition, the ground state p*K*a values for **8a** and **8b** were determined. There are two sites where deprotonation may occur—the 7-hydroxy substituent and the N(H) of the sulfonamide. Control experiments showed that **8a** is stable under the experimental conditions used for determining the p*K*a, Section S6, SI.

**Figure 4:**
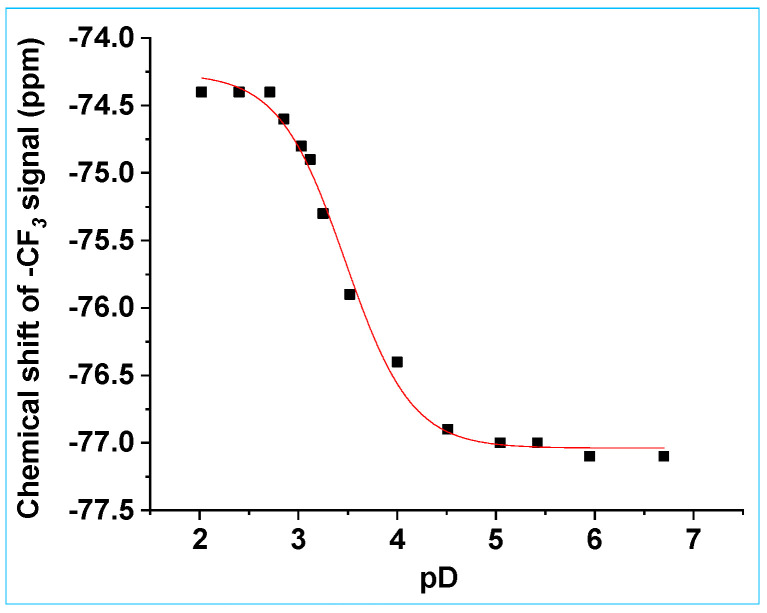
Chemical shift of the CF_3_ peak of **8a** versus pD. Data were fitted to Equation (1), giving p*K*a = 3.42 ± 0.02, δ_HA_ = −74.7 ± 0.1 ppm and δ_A−_ = −77.0 ± 0.1 ppm.

The p*K*a values of **8a** were initially investigated using NMR spectroscopy. The ^19^F and ^1^H NMR spectra of **8a** were recorded in D_2_O with the pD of the aqueous component of the solvent mixture varying from pD 2.03–11.23, to obtain an estimate of p*K*a values of both sites. A small amount of CH_3_CN (8% *v*/*v* CH_3_CN) was required to ensure that the compound was fully dissolved. The ^19^F NMR spectra are shown in Figure S10, SI, and a plot of chemical shift of the CF_3_ signal versus pD is shown in Figure 4. The chemical shift of the CF_3_ signal of **8a** moves upfield upon deprotonation of the N(H) proton, due to shielding by the electron-rich N^−^ proximate to the CF_3_ group.

Fitting the data in Figure 4 to the equation

(1) $$\delta_{\mathrm{obs}}=\frac{\delta_{\mathrm{HA}}+\delta_{A-}-(10^{\mathrm{pD}-pKa})}{1+(10^{\mathrm{pD}-pKa})}$$

where δ_obs_ = observed chemical shift (ppm), δ_HA_ = the chemical shift (ppm) of the acid form of **8a** and δ_A−_ = the chemical shift (ppm) of the conjugate base of **8a**, gives a p*K*a value of 3.42 ± 0.02. This p*K*a value is assigned to the N(H) proton of the *N*-hydroxysulfonamide, based on its value and the large changes in the chemical shift of the CF_3_ peak under varying pD conditions. ^1^H NMR spectroscopy was not useful for determining this p*K*a value, since the BHC-CH_2_-ON(H)-SO_2_CF_3_ peak overlapped with the HDO peak. The p*K*a value of 3.42 ± 0.02 is similar to that observed for 6,2-HNM-ON(H)-SO_2_CF_3_ (4.4 ± 0.1 in aqueous solution) [24] and 2-NPE-ON(H)-SO_2_CF_3_ (3.77 ± 0.03 (in D_2_O with 5% *v*/*v* CH_3_CN, *I* = 1.0 M, NaCF_3_SO_3_)) [26]. Essentially no change in the CF_3_ shift was observed from pD 5.05 to 11.22 (where the coumarinyl OH proton is lost) (≤0.2 ppm), because of the distal location of the CF_3_ moiety relative to the 7-hydroxy substituent.

**Figure 5:**
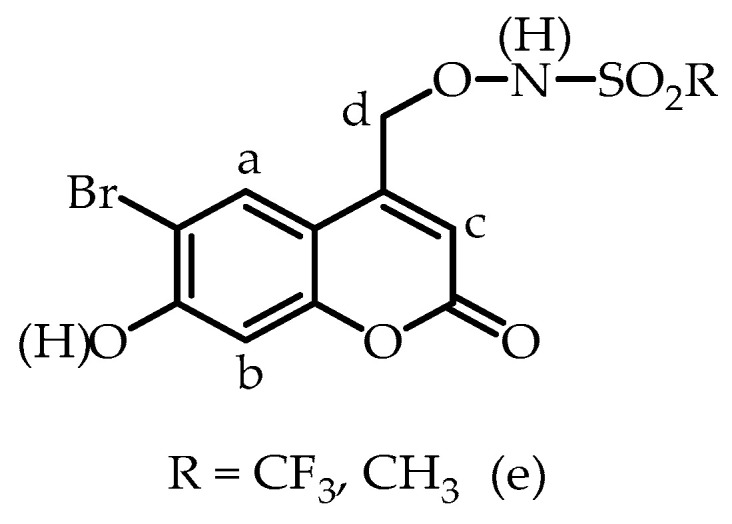
Proton assignments for **8a** and **8b**.

**Figure 6:**
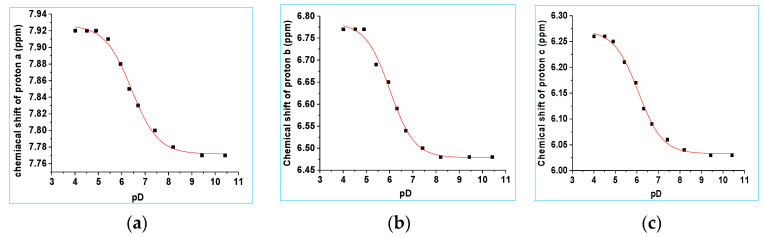
Plots of the chemical shift of protons **a**, **b** and **c** for **8a** versus pD. Data were fitted to Equation (1). (**a**) Proton **a**: p*K*a = 6.35 ± 0.03, δ_HA_ = 7.92 ± 0.01 ppm and δ_A−_ = 7.77 ± 0.01 ppm. (**b**) Proton **b**: p*K*a = 6.28 ± 0.03, δ_HA_ = 6.77 ± 0.01 ppm and δ_A−_ = 6.47 ± 0.01 ppm. (**c**) Proton **c**: p*K*a = 6.32 ± 0.03, δ_HA_ = 6.26 ± 0.01 ppm and δ_A−_ = 6.04 ± 0.01 ppm.

The p*K*a for deprotonation of the second ionizable site, the 7-hydroxy substituent of **8a**, was determined by recording ^1^H NMR spectra from pD 4.90 to 9.43. The assignments of the aromatic protons are shown in Figure 5. The chemical shifts were unchanged (≤0.02 ppm) from pD 2.02 to 4.92. Deprotonation of the O(H) moiety resulted in an upfield shift of chemical shifts **a**, **b**, and **c**, due to shielding effects by the more electron-rich aryloxy anion with increasing pD. As expected, greater shielding effects at high pD were seen for protons **b** and **c** due to resonance delocalization of the oxyanion to the carbons directly bearing these two protons. Selected ^1^H NMR spectra are shown in Figure S11, SI. The chemical shift of protons **d** overlapped with the HDO peak of the aqueous component. The chemical shifts of protons **a**, **b** and **c** were plotted as a function of pD, Figure 6, and data were fitted to Equation (1), giving p*K*a = 6.35, (proton **a**), p*K*a = 6.28 (proton **b**) and p*K*a = 6.32 (proton **c**), with an average p*K*a value of 6.32 ± 0.03. The p*K*a of 6-bromo-7-hydroxy-4-methylcoumarin was separately determined by UV–vis spectroscopy (p*K*a 6.91 ± 0.03 in 8:92 *v*/*v* CH_3_CN:H_2_O; Section S7, SI). p*K*a values of 6.2 (aqueous solution) and 5.88 (10:90 *v*/*v* DMSO:H_2_O) have been reported by others for the 7-hydroxy substituent of BHCM-OH and (6-bromo-7-hydroxycoumarin-4-yl)methyl acetate (BHCM-OAc), respectively [52].

**Figure 7:**
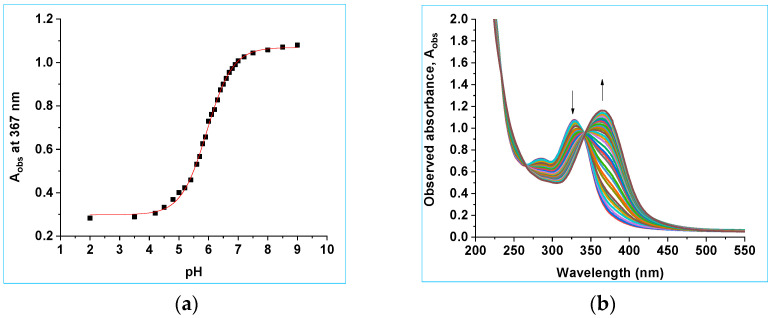
(**a**) UV–vis spectra of **8a** as a function of pH from pH 2.0 to 9.5 (8:92 *v*/*v* CH_3_CN:H_2_O). (**b**) Plot of absorbance at 367 nm versus pH. The data have been fitted to Equation (2), giving p*K*a_2_ = 6.35 ± 0.03, A_HA_ = 0.30 ± 0.03 and A_A−_ = 1.08 ± 0.03.

The ground state p*K*a for the 7-hydroxy substituent of **8a** was also determined using UV–vis spectroscopy. A control experiment showed that **8a** does not decompose under the conditions used for these experiments, Section S8, SI. Significant changes in the UV–vis spectra were only observed upon deprotonation of the O(H) and not the N(H), since the O(H) is an aromatic substituent while the N lone pair is not conjugated into the arene chromophore. As the O(H) site deprotonates, the wavelength maximum shifts from 330 nm to 365 nm, Figure 7a. The observed absorbance, A_obs_, at 367 nm was plotted as a function of pH, Figure 7b and the data fitted to Equation (2).

(2) $$A_{\mathrm{obs}}=\frac{A_{\mathrm{HA}}+A_{A-}-(10^{\mathrm{pH}-pKa})}{1+(10^{\mathrm{pH}-pKa})}$$

where A_HA_ = the absorbance of the conjugate acid and A_A−_ = the absorbance of the conjugate base. Fitting the data to Equation (2) gives p*K*a = 6.35 ± 0.03 (Figure 7b).

The p*K*a of the O(H) of **8a** determined by ^1^H NMR spectroscopy was 6.31 ± 0.03. This study was conducted in D_2_O with 8% *v*/*v* CH_3_CN, *I* = 1.0 M (CF_3_SO_3_Na). The value obtained by UV–vis spectroscopy was 6.35 ± 0.03, in 8:92 *v*/*v* CH_3_CN:H_2_O. The p*K*a values in H_2_O are typically 0.05–0.6 lower than in D_2_O [53,54,55]; that is, acids are stronger in H_2_O versus D_2_O. The differences in ionic strength will also have an effect on the p*K*a value.

### 2.5. Determination of the pKa Values for ***8b***

**Figure 8:**
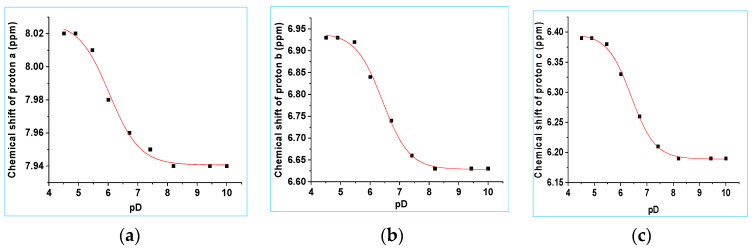
(**a**) Chemical shifts of protons **a**, **b** and **c** vs. pD. The data were fitted to Equation (1). (**a**) Proton **a**: p*K*a = 6.28 ± 0.01, δ_HA_ = 8.02 ± 0.01 ppm and δ_A−_ = 7.94 ± 0.01 ppm. (**b**) Proton **b**: p*K*a = 6.42 ± 0.03, δ_HA_ = 6.94 ± 0.01 ppm and δ_A−_ = 6.63 ± 0.01 ppm. (**c**) Proton **c**: p*K*a = 6.38 ± 0.03, δ_HA_ = 6.39 ± 0.01 ppm and δ_A−_ = 6.18 ± 0.01 ppm.

The p*K*a values for **8b** were initially investigated using NMR spectroscopy. ^1^H NMR spectra of **8b** were recorded with the pD of the aqueous component of the D_2_O/CH_3_CN (8% *v*/*v* CH_3_CN, *I* = 1.0 M, CF_3_SO_3_Na) solvent mixture varying from pD 2.52–11.03. A control experiment showed that **8b** does not decompose under the pH(D) conditions of these experiments, Section S9, SI. Selected ^1^H NMR spectra are shown in Figures S13 and S14, SI. As seen for compound **8a** (see above), the increase in electron density on the aromatic ring upon deprotonation of the O(H) substituent of **8b** leads to shielding of all three aryl protons, with resonance effects leading to greater shielding for protons **b** and **c**. The chemical shift of protons **a**, **b** and **c** were plotted as a function of pD (Figure 8a–c). Fitting the data to Equation (1) gave p*K*a (proton **a**) = 6.28, p*K*a (proton **b**) = 6.42, p*K*a (proton **c**) = 6.38 (I = 1.0 M, NaCF_3_SO_3_). Averaging the values from the two experiments where substantial changes in chemical shift were seen (protons **b** and **c**) gave an average p*K*a value of 6.40 ± 0.03. This p*K*a value is assigned to deprotonation of the OH substituent of **8b**. The chemical shift of protons **a**, **b** and **c** were unchanged from pD 2.52 to 4.90 and from pD 10.30 to 11.03 ppm. Protons **e** (the CH_3_ group) are 6 bonds away from the aromatic group, and as expected, their chemical shift is unchanged in this region within experimental error (pD 2.52–9.75).

**Figure 9:**
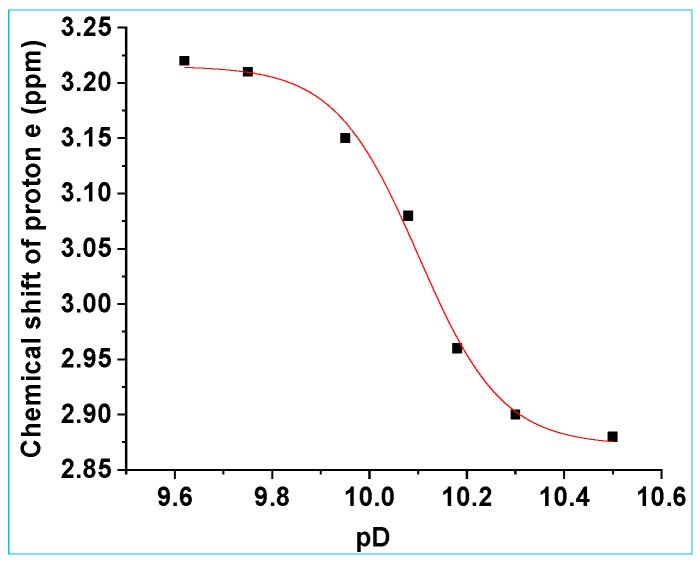
^1^H NMR chemical shift (ppm) for the CH_3_ protons (protons **e**) of **8b** as a function of pD, in D_2_O with 8% *v*/*v* CH_3_CN, *I* = 1.0 M, CF_3_SO_3_Na. Data were fitted to Equation (1), giving p*K*a = 10.11 ± 0.03, δ_HA_ = 3.22 ± 0.01 ppm and δ_A−_ = 2.87 ± 0.01 ppm.

The chemical shift of the CH_3_ (**e**) proton signal moved upfield from 3.22 ppm to 2.88 ppm as pD of the solution was increased from 9.62 to 10.50, Figure S14, SI (^1^H NMR spectra). Since proton **e** is adjacent to the N(H) moiety of the *N*-hydroxysulfonamide of **8b**, the change in the chemical shift is attributed to deprotonation of this site. The chemical shift of the protons **e** was plotted as a function of pD (Figure 9). Fitting the data to Equation (1) gives p*K*a = 10.11 ± 0.03. Like **8a**, the BHC-CH_2_-ON(H)-SO_2_CH_3_ peak overlapped with the HDO peak from the solvent, so protons **d** could not be used to determine the p*K*a of the N(H) proton of **8b**. This p*K*a value is very similar to the p*K*a for the N(H) in 2-NPE-ON(H)-SO_2_CH_3_ (10.06, in D_2_O with 5% *v*/*v* CH_3_CN, *I* = 1.0 M, CF_3_SO_3_Na [26]).

**Figure 10:**
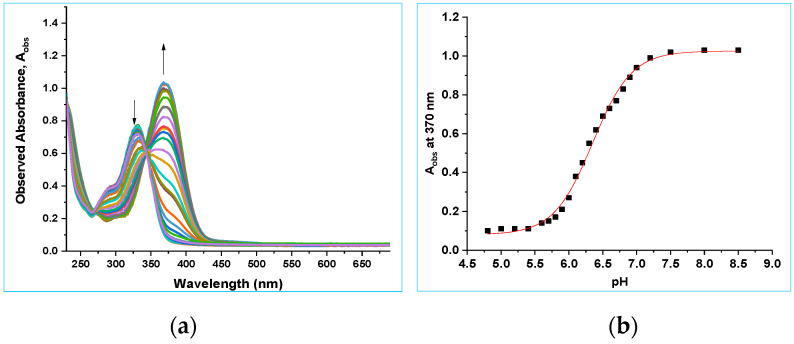
(**a**) UV–vis spectra of **8b** as a function of pH from pH 4.4–8.7 (8:92 *v*/*v* CH_3_CN:H_2_O). (**b**) Plot of absorbance at 370 nm versus pH. The data have been fitted to Equation (2), giving p*K*a_2_ = 6.47 ± 0.03, A_HA_ = 0.10 ± 0.03 and A_A−_ = 1.03 ± 0.03.

The p*K*a value of **8b** was also investigated by UV–vis spectroscopy. A UV–vis spectroscopic titration experiment for **8b** was carried out to determine the p*K*a value of the O(H) substituent. A UV–vis spectrophotometric titration of **8b** (3.1 × 10^−5^ M, in 8:92 *v*/*v* CH_3_CN:H_2_O, 30 mL) was conducted using the same flow set up used for **8a** (Figure 10a). The absorbance at 370 nm was plotted as a function of pH. Data were fitted to Equation (2), giving p*K*a = 6.47 ± 0.03 (8:92 *v*/*v* CH_3_CN:H_2_O, Figure 10b). The p*K*a for the O(H) proton from the NMR spectroscopic studies reported above was 6.40 ± 0.03 (in D_2_O with 8% *v*/*v* CH_3_CN, *I* = 1.0 M, CF_3_SO_3_Na). This is once again consistent with the p*K*a values in H_2_O being typically 0.05–0.6 lower than in D_2_O [53,54,55].

**Scheme 3:**
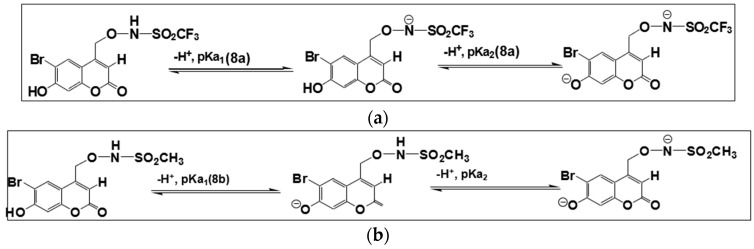
Ground state acid dissociation equilibria for (**a**) **8a**, p*K*a_1_ =3.42 ± 0.02 (in D_2_O with 8% *v*/*v* CH_3_CN, *I* = 1.0 M, CF_3_SO_3_Na), p*K*a_2_ = 6.31 ± 0.03 (in D_2_O with 8% *v*/*v* CH_3_CN, *I* = 1.0 M, CF_3_SO_3_Na) and 6.35 ± 0.03 (in 8:92 *v*/*v* CH_3_CN:H_2_O). (**b**) **8b**, p*K*a_1_ = 6.40 ± 0.03 (in D_2_O with 8% *v*/*v* CH_3_CN, *I* = 1.0 M, CF_3_SO_3_Na) and p*K*a_1_ = 6.47 ± 0.03 (in 8:92 *v*/*v*, CH_3_CN:H_2_O), p*K*a_2_ = 10.11 ± 0.03 (in D_2_O with 8% *v*/*v* CH_3_CN, *I* = 1.0 M, CF_3_SO_3_Na).

To summarize, two p*K*a values were determined for **8a** and **8b** using both NMR and UV–vis spectroscopy. For **8a**, the N(H) site deprotonates first followed by the O(H) site. For **8b**, the O(H) site instead deprotonates first followed by the N(H) site. Deprotonation sites for **8a** and **8b** are shown in Scheme 3.

### 2.6. Effect of pH on the Photoproducts

The effect of the pH of the aqueous component of a solvent mixture on the photoproducts obtained after irradiation of **8a** was investigated in 10% *v*/*v* CD_3_CN in aqueous solution. Prior to doing these experiments, the stability of **8a** under the ambient red-light conditions used for these experiments was checked at pH 2.1, 5.0, 7.0 and 10.0 using ^1^H and ^19^F NMR spectroscopy. **8a** (1.0 mM) was found to be photostable for at least 210 min under these conditions.

Figure S15a in Section S10, SI, shows ^19^F NMR spectra of **8a** recorded before and after 1.0 and 3.0 min total irradiation, in a solution of 10:90 *v*/*v* CD_3_CN:phosphate buffer, pH 2.1. At pH 2.1, only CF_3_SO_2_NH_2_ (O-N bond cleavage) was observed. To characterize the aromatic photoproducts at this pH condition, the ^1^H NMR spectrum of a partially decomposed sample (1.0 min irradiation) was recorded, Figure S15b. The chemical shifts at 10.01, 8.66, 8.11 and 7.00 ppm were assigned to BHC-CHO using authentic BHC-CHO.

Photolysis experiments were also conducted at other pH conditions. Figures S15a–S18a show the ^19^F NMR spectra of fully photodecomposed samples of **8a** at pH 2.1, 5.0, 7.0 and 10.0, respectively (10:90 *v*/*v* CD_3_CN:30 mM acetate buffer (pH 5.0), 30 mM phosphate buffer (pH 7.0) or 30 mM carbonate buffer (pH 10.0)). The results are summarized in Table 2. The fluorinated photoproducts were CF_3_SO_2_^−^ (~80%) and CF_3_SO_2_NH_2_ (~20%) at pH 5.0, 7.0 and 10.0, whereas O-N bond heterolysis dominates at pH 2.1 (96% CF_3_SO_2_NH_2_). ^1^H NMR spectra after partial photodecomposition showed the expected signals from BHC-CHO (**12**), BHCM-OH (**3**), (*E*)-BHC-oxime (**13a**) and (*Z*)-BHC-oxime (**13b**), with peaks assigned using authentic samples of these compounds (Figures S15b–S18b and Tables S1 and S2, SI).

The effect of the pH on the photoproducts obtained upon irradiating **8b** was determined under anaerobic conditions in 10% *v*/*v* CD_3_CN in aqueous buffered solution, Section S11, SI. The photoproducts generated at pH 2.5, 5.0, 7.0, 10.0 and 10.7 were characterized using ^1^H NMR spectroscopy, using both fully and partially photolyzed samples. Spectra are shown in Figures S19–S22. Table 3 summarizes the percentages of the aliphatic photoproducts upon complete photodecomposition. The percentage of each photoproduct is unchanged from pH 2.1 to pH 7.0 within experimental error, with O-N bond cleavage (~80%) dominating to give CF_3_SO_2_NH_2_ and BHC-CHO (**12**). At pH 10.0 and 10.7 both O-N bond cleavage (40% CH_3_SO_2_NH_2_) and concomitant C-O/N-S bond cleavage (48–54% CF_3_SO_2_^−^) bond cleavage pathways are important. The amount of C-O bond cleavage is small and is almost pH independent within experimental error. Peaks were observed in partially irradiated samples that could be attributed to BHCM-OH (**3**) and BHC-CHO (**12**).

### 2.7. Effect of the Excitation Wavelength on the Photoproducts

The photoproducts obtained upon irradiation of **8a** and **8b** (1.0 mM) at two different excitation wavelengths (355 and 270 nm) were investigated in a mixture of phosphate buffer (30 mM, pH 7.0) and CD_3_CN (40:60, *v*/*v*), using a xenon lamp in conjunction with a monochromator. In each system, the excitation wavelength did not have any effect on the observed photoproducts (Table S3, SI). This is consistent with a π–π* transition occurring for this system regardless of the excitation wavelength.

### 2.8. Effect of Triplet Quenchers on the Photoproducts

Steady state photolysis experiments were carried out in the presence of the triplet quenchers *p*-terphenyl and cyclohexadiene [56]. Photolyzing **8b** in the presence of 20 mol equivalent *p*-terphenyl or 20 mol equivalent cyclohexadiene under aerobic conditions in a mixture of phosphate buffer (30 mM, pH 7.0) and CD_3_CN (40:60, *v*/*v*) gave CF_3_SO_2_NH_2_ (78%), CH_3_SO_2_NHOH (7%), CH_3_SO_2_^−^ (4%), CH_3_SO_3_^−^ (3%) and 8% of an unknown species. Under anaerobic conditions the photoproducts were CH_3_SO_2_NH_2_ (77%), CH_3_SO_2_NHOH (7%), CH_3_SO_2_^−^ (2%), CH_3_SO_3_^−^ (7%) and an unknown species (7%). The presence of the triplet quenchers oxygen, *p*-terpene or cyclohexadiene had no significant effect on the photoproducts and hence the mechanism of photodecomposition (Pathways 1–3, Scheme 2).

### 2.9. Photoproduct Quantum Yield

The photoproduct quantum yields for **8a** and **8b** at 313 nm were determined by actinometry, using the isomerization of *trans*-azobenzene to its *cis* isomer as a reference compound (Φ = 0.14 at 313 nm in CH_3_OH [57]). Details are given in Section S13, SI. The percentage of *trans*-azobenzene converted to *cis*-azobenzene upon irradiation was determined by UV–vis spectroscopy, whereas the photodecomposition of **8a** and **8b** was assessed using ^19^F or ^1^H NMR spectroscopy. All the experiments were carried out on the same day. The photoproduct quantum yields were 0.038 (**8a**) and 0.079 (**8b**). These values are within the range typically observed for (coumarin-4-yl)methyl caged systems [29,58]. The photoproduct quantum yields for **8a** and **8b** are lower than those reported for the corresponding *N*-hydroxysulfonamide systems caged with the (6-hydroxynapthalen-2-yl)methyl (0.48 (R = CF_3_), in 60:40 *v*/*v* CH_3_CN:5.0 mM phosphate buffer solution) [24], 2-(2-nitrophenyl)ethyl (0.47 (R = CF_3_) and 0.32 (R = CH_3_); in CH_3_OH) [26] and 2-nitrobenzyl (0.67 (R = CF_3_) and 0.77 (R = CH_3_); in CH_3_OH) [25] chromophores. **8a** and **8b** are fluorescent (data not shown). Fluorescence competes with bond cleavage in (coumarin-4-yl)methyl caged systems [59], resulting in lower photoproduct quantum yields.

## 3. Discussion

Three (6-bromo-7-hydroxycoumarin-4-yl)methyl-caged *N*-hydroxysulfonamides were synthesized. Detailed mechanistic studies were carried out for two of these compounds (**8a** and **8b**). Studies by others of molecules photocaged with the (6-bromo-7-hydroxycoumarin-4-yl)methyl moiety have shown that for these systems, upon π–π* excitation, internal conversion to the lowest-energy singlet excited state occurs, followed by rapid heterolytic C-O bond cleavage, to release the molecule of interest and generate a carbocation intermediate [27,29,57,58,60]. The carbocation rapidly reacts with nucleophiles including solvent H_2_O or CH_3_OH [61].

For both of our systems, the photoproduct percentages were not affected by the excitation wavelength selected using a xenon lamp in conjunction with a monochromator, consistent with π–π* excitation occurring regardless of the excitation wavelength. The triplet state quenchers oxygen (aerobic versus anaerobic conditions), *p*-terphenyl and cyclohexadiene had no significant effect on the observed percentages of the photoproducts, which indicates no involvement of triplet excited state species in the bond cleavage events leading to the primary photoproducts. Hence, both **8a** and **8b** undergo bond cleavage from singlet excited state species. The photoproduct quantum yields of both systems were also determined. The photoproduct quantum yield values for **8a** and **8b** were 0.038 and 0.079, respectively, at 313 nm in CH_3_OH. The quantum yield of 6-bromo-7-hydroxy-4-methylcoumarin was reported to be 0.13 (350 nm, aqueous solution) [36].

The p*K*a of the O(H) of the coumarin photocage and N(H) of the *N*-hydroxysulfonamide moiety played a crucial role in determining the mechanism of photodecomposition for the analogous (6-hydroxynapthalen-2-yl)methyl (6,2-HNM) photocaged *N*-hydroxytrifluoromethanesulfonamide system (6,2-HNM-ON(H)SO_2_CF_3_) [62]. This latter molecule also generates a carbocation, (H)NO and CF_3_SO_2_^−^ upon rapid concomitant C-O/N-S heterolytic bond cleavage in the lowest singlet excited state of this system [62]. The ground state p*K*a values for the N(H) and O(H) protons of **8a** and **8b** were therefore determined using a combination of ^19^F NMR spectroscopy, ^1^H NMR spectroscopy and UV–vis spectroscopy titration experiments. The p*K*a value for the N(H) proton of **8a** was 3.42 ± 0.02 (8:92 *v*/*v* CH_3_CN:D_2_O, *I* = 1.0 M, CF_3_SO_3_Na). The p*K*a of the O(H) of the coumarin photocage of **8a** was 6.31 ± 0.03 (8:92 *v*/*v* CH_3_CN:D_2_O, *I* = 1.0 M, NaCF_3_SO_3_; ^1^H NMR spectroscopy) and 6.35 ± 0.03 (in 8:92 *v*/*v* CH_3_CN:H_2_O); UV–vis spectroscopy). The electron-withdrawing ON^−^SO_2_CF_3_ moiety of **8a** decreases the p*K*a of the O(H) proton compared with 6-bromo-7-hydroxy-4-methylcoumarin. This group still behaves as an electron-withdrawing group, despite the deprotonation at the N center.

The p*K*a values were also determined for **8b**. The p*K*a of the N(H) of **8b** is 10.11 ± 0.03 (8:92 *v*/*v* CH_3_CN:D_2_O, *I* = 1.0 M, NaCF_3_SO_3_, ^1^H NMR spectroscopy). The p*K*a of the O(H) was found to be 6.47 ± 0.03 for **8b** (8:92 *v*/*v* CH_3_CN:H_2_O) using UV–vis spectroscopy. The p*K*a of the O(H) in these systems therefore follows the order **8a** (6.35 ± 0.03) < **8b** (6.47 ± 0.03) < 6-bromo-7-hydroxy-4-methylcoumarin (6.91 ± 0.03). Interestingly, this order indicates that the electron-withdrawing ability of the deprotonated ON^−^SO_2_CF_3_ moiety in **8a** is slightly better than the neutral N(H)SO_2_CH_3_ group in **8b**.

Studying the effect of varying the volume percentages of phosphate buffer (pH 7.0) and CD_3_CN gave valuable information on the mechanisms of photodecomposition of **8a**. Analysis of the photoproducts showed that two pathways for decomposition occur—concomitant C-O/N-S bond cleavage and O-N bond cleavage, Pathways 1 and 3, Scheme 2. The aromatic photoproducts observed in partially photolyzed solutions were BHCM-OH and (*E*)- and (*Z*)-BHC-oximes from C-O/N-S bond cleavage, Pathway 1, and BHC-CHO from O-N bond cleavage, Pathway 3. The generation of HNO was also demonstrated, using an established phosphine trapping agent, which reacts with HNO to produce the characteristic phosphine ylide; hence not all the released NO^−^ generated upon C-O/N-S bond cleavage is trapped by the carbocation intermediate to give an oxime. The amount of C-O/N-S bond cleavage progressively decreases as the percentage of phosphate buffer in the solvent mixture, Figure 2. Hence, increasing the volume percentage of H_2_O in the solvent mixture results in a decrease in the desired HNO-releasing pathway. This result parallels what was observed for the 6,2-HNM-ON(H)SO_2_CF_3_ system [24]. However, only O-N bond cleavage was observed for 6,2-HNM-ON(H)SO_2_CF_3_ in pure CD_3_CN, whereas 36% C-O/N-S bond cleavage occurs for **8a** in this solvent. For 6,2-HNM-ON(H)SO_2_CF_3_ it was proposed that the addition of a trace amount of water or another weak base was required for C-O/N-S bond cleavage since the N(H) of the *N*-hydroxysulfonamide of the reactant must be deprotonated [23]. DFT calculations are currently underway, with preliminary results suggesting that deprotonation of the N(H) of the reactant is required for HNO generation. One important difference between the two systems is the excited state p*K*a* for the O(H), with the value anticipated to be lower for (6-bromo-7-hydroxycoumarin-4-yl)methyl-caged molecules (p*K*a* for O(H) = 0.9 for 7-hydroxy-4-methylcoumarin compared with 6,2-HNM-ON(H)SO_2_CF_3_ (p*K*a* for O(H)~3.4 ± 0.4)) [62,63]. Unfortunately, a fluorescence titration experiment could not be conducted to obtain an estimate of p*K*a* for the O(H) substituent of the singlet excited state of **8a**, since the compound was not stable at pH values less than 0.9.

The effect of pH on the photoproducts and hence the mechanism of photodecomposition was also studied for **8a**, in 10:90 *v*/*v* mixtures of CD_3_CN:aqueous buffer. When the pH of the aqueous component was pH 2.1, essentially only O-N bond cleavage was observed. However, at pH 5.0, 7.0 and 10.0, 20% CF_3_SO_2_^−^ and 80% CF_3_SO_2_NH_2_ was observed at all pH conditions. This is consistent with deprotonation of the N(H) of **8a** being essential for concomitant C-O/N-S bond cleavage (p*K*a 3.42 ± 0.02 in 8:92 *v*/*v* CH_3_CN:D_2_O, *I* = 1.0 M, NaCF_3_SO_3_). Interestingly, deprotonation of the 6-hydroxy substituent of **8a** (p*K*a 6.35 ± 0.03 in 8:92 *v*/*v* CH_3_CN:H_2_O) had no effect on the percentages of the observed photoproducts in 92:8 *v*/*v* mixtures of aqueous buffer:CD_3_CN. Given that the N(H) is some distance from the chromophore and that bond conjugation does not extend to this site, p*K*a(NH)* is probably not that different from the ground state p*K*a(NH). However, this will not be the case for the 6-hydroxy substituent of the coumarin, as it is well-known that the p*K*a of aromatic OH groups can drop several orders of magnitude upon excitation of the molecule [63], consistent with a p*K*a* value of 0.9 for the 7-hydroxy substituent of 7-hydroxy-4-methylcoumarin [63]. Hence, it is likely that this site is deprotonated in the excited state at even the lowest pH condition (pH 2.1) of this study. Unpublished data from our labs show that substituting the OH substituent of both 6,2-HNM-ON(H)SO_2_CF_3_ and **8a** with a non-ionizable group (the methoxymethyl ether (MOM) protecting group) does lower the amount of O-N bond cleavage in this system. We hypothesize that the main roles of H_2_O are (i) to promote H_2_O-assisted deprotonation of the methylene carbon in the excited state molecule, leading to O-N bond cleavage and generation of RSO_2_NH_2_, and (ii) to deprotonate the N center (especially in **8a**) which will favor the desired C-O/N-S cleavage pathway versus the O-N bond cleavage pathway. The presence of a neutral strongly electron-withdrawing RSO_2_N(H)O- group will assist in weakening the C-H methylene bond to facilitate O-N cleavage, while the deprotonated RSO_2_N^−^O- group will be less effective in weakening this C-H bond and so will presumably disfavor O-N bond cleavage.

Examination of the results for the related **8b** system revealed some key differences. Firstly, unlike the **8a** system, increasing the volume percentage of aqueous phosphate buffer (pH 7.0) in CD_3_CN/phosphate buffer mixtures does not have much effect on the photoproduct percentages, Table 1. For this system, 79–93% CH_3_SO_2_NH_2_ was observed. Others have reported that CH_3_SO_3_^−^ can be formed from the reaction of CH_3_SO_2_^−^ with the hydroxyl radical [64]. Trace amounts of the hydroxyl radical are generated upon photolysis of aqueous solutions. Importantly, the p*K*a of the N(H) of **8b** was 10.11 ± 0.03 (in D_2_O with 8% *v*/*v* CH_3_CN, *I* = 1.0 M, CF_3_SO_3_Na); hence generation of HNO via C-O/N-S bond cleavage is not favorable since this site is protonated in all but the most basic solvent systems studied. This conclusion is supported by the results obtained upon varying the pH of the aqueous component for 92:8 *v*/*v* aqueous solution:CD_3_CN mixtures, Table 3. Specifically, while the amount of HNO generation remained low when the pH of the aqueous component was pH 5.0 and 7.0 (~3% CH_3_SO_2_^−^ and ~5% CH_3_SO_3_^−^), at pH 10.0 and 10.7 the percentage of C-O/N-S bond cleavage increased 15-fold (~50% CH_3_SO_2_^−^, 2–8% CH_3_SO_3_^−^), consistent with deprotonation of the N(H) of the sulfonamide playing a key role in favoring C-O/N-S cleavage over O-N bond cleavage during the photodecomposition even for **8b**. Note that the percentage of C-O/N-S bond cleavage is even higher for **8b** than that observed for **8a** in a highly basic solvent mixture (48–54% CH_3_SO_2_^−^ versus 19% CF_3_SO_2_^−^ in 10:90 % *v*/*v* CD_3_CN:aqueous pH 10.0 buffer). Hence, it is clear that the electron-withdrawing effect of the CF_3_SO_2_ group of **8a** promotes O-N bond cleavage, while the concomitant lowering of the p*K*a of the proximal NH proton by the same group favors C-O/N-S cleavage over O-N cleavage. Under more neutral photolytic conditions, this latter effect dominates to favor C-O/N-S cleavage over O-N cleavage for **8a** (where the NH is deprotonated), while for **8b** the neutral N(H)SO_2_CH_3_ under these conditions favors O-N bond cleavage.

Nakagawa et al. recently reported the synthesis of (7-diethylaminocoumarin-4-yl)methyl photocaged derivatives of the Piloty’s acid derivatives (2-Br)PhSO_2_NHOH and (2-NO_2_)PhSO_2_NHOH [21]. HNO generation was confirmed indirectly by observation of N_2_O formation by GC-MS, observing the formation of the sulfinamide and disulfide products from the reaction of HNO with *N*-acetylcysteine, and an enhancement in fluorescence upon binding to an HNO-specific fluorescence probe. The corresponding oxime compound was also observed upon irradiation of the photocaged 2-nitro derivative of Piloty’s acid and the authors speculated that the HNO-generating pathway proceeds via C-O bond cleavage to generate the carbocation intermediate and the anion of the parent *N*-hydroxysulfonamide. The latter molecule would then decompose further to generate HNO and the sulfinate product. Alternatively, subsequent nucleophilic attack of the N lone pair of the anion of the *N*-hydroxysulfonic acid was proposed, to give the oxime product via a nitroso compound which subsequently tautomerizes. The possible role of the protonation state of the N(H) was not investigated in this study and there was no experimental evidence for C-O bond cleavage to give the anion of the parent *N*-hydroxysulfonic acid in addition to the carbocation. Based on our results, we think that deprotonation of the N(H) will also be required for HNO generation for these two systems, and that the HNO-generating pathway instead likely involves concomitant C-O/N-S bond cleavage. It is likely that NO^−^ is released and is then trapped by the resulting carbocation before it can escape the solvent cage.

## 4. Conclusions

Three photoactive *N*-hydroxysulfonamides photocaged with the (6-bromo-7-hydroxy-coumarin-4-yl)methyl chromophore have been successfully synthesized, and the mechanisms of photodecomposition investigated for **8a** and **8b**. The p*K*a values for the N(H) and O(H) sites were determined for the ground state molecules by UV–vis and/or NMR spectroscopy titration experiments. The release of HNO from these molecules depends on the pH of the aqueous component of the system, with deprotonation of the N(H) proton required for efficient concerted C-O/N-S bond cleavage to give a carbocation, NO^−^ and a sulfinate. The protonation state of the O(H) does not have any effect on the observed photoproducts. In the absence of a species that reacts rapidly with (H)NO, this species reacts with the carbocation intermediate to ultimately generate (*E*)-BHC-oxime and (*Z*)-BHC-oxime. However, O-N bond cleavage competes with the desired HNO-generating pathway and becomes more favorable when the volume percentage of water in acetonitrile/water solvent mixtures is increased. We hypothesize that one of the roles of water is to deprotonate the methylene carbon in the excited state molecule, which leads to O-N bond cleavage. Additionally, water can solvate the developing anionic leaving group, stabilizing the transition state required for O-N bond cleavage. Water is also expected to stabilize the carbocation intermediate generated upon C-O and concomitant C-O/N-S bond cleavage.

With photolysis under neutral or basic conditions, only C-O/N-S and O-N bond cleavage are observed for **8a**, whereas a small amount of C-O bond cleavage is also observed for **8b**. From a comparison of the photoproducts at pH conditions where the N(H) is deprotonated for both systems, it is clear that the presence of the electron-withdrawing CF_3_SO_2_ moiety of **8a** promotes undesired O-N bond cleavage compared with the **8b** system, presumably by weakening the C-H bond and lowering the p*K*a of the methylene proton, resulting in O-N bond cleavage. It is likely that bond cleavage occurs in the singlet excited state as observed for a closely related (6-hydroxynapthalen-2-yl)methyl-photocaged system [62], since triplet quenchers had no effect on the observed photoproducts. To summarize, detailed mechanistic studies have allowed us to obtain fundamental information of the factors that ultimately determine the pathway(s) of photodecomposition for photoactive Piloty’s acid-based systems.

## 5. Materials and Methods

### 5.1. General Information

All precursors, reagents and solvents were purchased from commercially available sources and used without purification, unless otherwise stated. Reagent-grade dichloromethane (CH_2_Cl_2_) was distilled from CaH_2_ before use. Anhydrous THF and anhydrous pyridine were purchased from commercial sources. Flash column chromatography was performed using silica gel (60 Å, 40–63 μm or 230–400 mesh), typically with elution using ACS-grade ethyl acetate and petroleum ether (boiling point range: 30–60 °C). Commercial anhydrous ethanol was used for trituration. All solid products were ground and dried in high *vacuo* before characterizing by ^1^H, ^13^C and ^19^F NMR spectroscopy.

NMR spectra were recorded using a Bruker 400 MHz NMR spectrometer with a 5 mm probe at 25 ± 1 °C and analyzed by MestReNova (MNOVA, version 11.3) or TOPSPIN NMR software (version 4.3.0). TSP (3-(trimethylsilyl)propionic-2,2,3,3-d_4_ acid, sodium salt) was used as an internal reference standard for ^1^H NMR spectroscopy, and an α,α,α-trifluorotoluene solution in CD_3_CN (−62.9 ppm) was used as an external reference standard for ^19^F NMR spectroscopy. Anaerobic NMR solutions were prepared and transferred to NMR tubes in a glovebox.

Preparation of the anaerobic sample solutions was carried out inside an MBraun Labmaster 130 glove box equipped with O_2_ and H_2_O sensors. Solutions were prepared in dim or red light conditions. Buffer solutions (0.10 M and 30 mM; phosphate, acetate or carbonate buffers) were adjusted to the desired pH (pD) with the addition of a solution of either HCl or NaOH solutions and subsequently degassed with argon or nitrogen.

pH measurements were carried using a Thermo Scientific Orion Star A211 pH meter connected to Mettler-Toledo pH combination micro electrode or an Orion Model 710A pH meter equipped with Mettler-Toledo Inlab 423 or 421 electrodes. Measurements were carried out at room temperature. The pH was adjusted using either NaOH or HCl solutions in water or D_2_O. For solvent mixtures the reported pH (pD) value is for the aqueous component.

High-Resolution mass spectra were performed using an Exactive Plus mass spectrometer (Thermo Scientific, Bremen, Germany). Mass spectra were recorded in the positive ionization mode with a scan range of 50–700 *m*/*z*, a mass resolving power setting of 140,000. The reported compounds were measured as pure solids ionized by electronically excited Helium gas using an ID-cube DART-source (Ionsense, Saugus, MA, USA). Isotopic patterns were simulated using the software Thermo Xcalibur 3.0.63 (Thermo Scientific, Bremen, Germany).

### 5.2. Photolysis Experiments

A Rayonet mini-photoreactor (RMR-600, 8 bulbs, 4 W) equipped with a fan was used to irradiate the sample solutions using 350 nm light bulbs (8 bulbs, 4 W). The samples (~1.0 mM) were prepared under anaerobic conditions inside a glovebox by dissolving in a mixture of buffer and CD_3_CN and transferred in air-tight J-Young quartz NMR tubes. After each irradiation, the sample was analyzed by ^1^H and/or ^19^F NMR spectroscopy.

### 5.3. Determination of pKa by UV–Vis Spectroscopy

**8a** or **8b** were dissolved in a mixture of aerobic CH_3_CN and H_2_O (8:92 *v*/*v* CH_3_CN:H_2_O). The solution was circulated using a peristaltic pump through a 1 cm path length quartz flow-through cell at 25.0 °C. A small volume of acid (~1 M HCl; negligible change in total volume of the solution) was added to the solution (3.5 × 10^−5^ M, 30 mL). Absorbance spectra were recorded after the pH had stabilized in the reservoir flask.

### 5.4. Determination of pKa by NMR Spectroscopy

The following aqueous solutions were prepared in D_2_O: 0.10 M HCl (pD 1.4), phosphate buffer (30 mM, pD 2.4–3.9 and 6.2–8.2), acetate buffer (30 mM, pD 4.0–6.0), borate buffer (30 mM, pD 8.9–9.9), carbonate buffer (30 mM, pD 9.6–11.0), and 0.010 M NaOH (pD 12.4). The pD of the deuterated buffered solutions were adjusted to the desired value using either HCl or NaOH (pD = pH + 0.4) [65]. A small volume of **8a** or **8b** dissolved in CH_3_CN was then added to an aerobic aqueous buffered solution. The final solution was ~1.0 mM, with 8% *v*/*v* CH_3_CN in deuterated aqueous buffer. A constant ionic strength of 1.0 M was maintained, by the addition of NaCF_3_SO_3_.

### 5.5. Determination of the Photoproduct Quantum Yields (Φ)

The photoproduct quantum yields for **8a** and **8b** at 313 nm were determined by actinometry in aerobic CH_3_OH, using the isomerization of *trans*-azobenzene to its *cis* isomer as a reference compound (Φ = 0.14 at 313 nm [57]), following our earlier published procedure [25]. The percentage of *trans*-azobenzene converted to *cis*-azobenzene upon irradiation was followed by UV–vis spectroscopy, whereas the photodecomposition of **8a** (1.00 mM) and **8b** (1.00 mM) was followed by ^19^F or ^1^H NMR spectroscopy.

### 5.6. Synthesis of HNO Donors BHC-ONHSO_2_R (R = CF_3_ (***8a***), CH_3_ (***8b***) and (2-SO_2_Me)Ph- (***8c***)) and the Related Photoproducts

#### 5.6.1. 6-Bromo-4-chloromethyl-7-hydroxycoumarin (**2**)


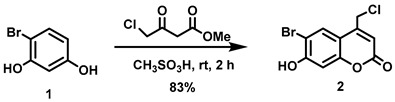


Methyl 4-chloroacetoacetate (4.82 mL, *d* = 1.305 g/mL at 25 °C, 41.8 mmol) was added all at once to a stirred solution of 4-bromoresorcinol (**1**, 5.26 g, 27.8 mmol) in methanesulfonic acid (40 mL) under nitrogen at room temperature. The reaction mixture was stirred for 2 h at room temperature before the resulting reddish-brown solution was slowly poured into ice-water (200 mL) and stirred for 30 min to give an off-white precipitate. The precipitate was collected by vacuum filtration, washed with cold water (3 × 50 mL) and dried in vacuo. The off-white solid was triturated with a 20:80 mixture of ethyl acetate/petroleum ether (100 mL), filtered, washed with petroleum ether, and dried in vacuo to afford the title compound as a pale pink solid (6.66 g, 83%). ^1^H NMR (400 MHz, DMSO-*d*_6_) δ 11.57 (s, 1H), 8.00 (s, 1H), 6.92 (s, 1H), 6.48 (t, 1H, *J* = 0.8 Hz), 5.00 (d, 2H, *J* = 0.8 Hz) [30].

#### 5.6.2. 6-Bromo-7-hydroxymethylcoumarin (**3**)


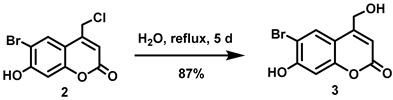


A stirred suspension of compound **2** (1.86 g, 6.42 mmol) in water (500 mL) was heated under reflux for 5 days before the resulting suspension was allowed to cool to room temperature. The precipitate settled down at the bottom of the flask. Most of the water from the top was carefully decanted and the remaining water was removed in vacuo. The resulting off-white residue was triturated with diethyl ether/petroleum ether (1:2, 240 mL) and filtered off to afford the title compound as an off-white solid (1.51 g, 87%). ^1^H NMR (400 MHz, DMSO-*d*_6_) δ 11.41 (s, 1H), 7.85 (s, 1H), 6.90 (s, 1H), 6.27 (t, 1H, *J* = 1.6 Hz), 5.63 (t, 1H, *J* = 5.6 Hz), 4.70 (dd, 2H, *J* = 5.6, 1.6 Hz) [32].

#### 5.6.3. 6-Bromo-7-O-(methoxymethyl)-4-hydroxymethylcoumarin (**4**)


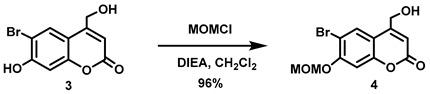


Anhydrous *N*,*N*-diisopropylethylamine (DIPEA, 3.21 mL, *d* = 0.742 g/mL at 25 °C, 18.4 mmol) was added all at once to a stirred suspension of **3** (5.12 g, 18.9 mmol) in anhydrous CH_2_Cl_2_ (210 mL) at room temperature under Ar. The resulting brown suspension was cooled to 0 °C before adding chloromethyl methyl ether (MOMCl, 1.36 mL, *d* = 1.06 g/mL at 25 °C, 18.0 mmol) dropwise over 5 min at 0 °C. The resulting brown suspension was stirred at 0–16 °C for 2 h before being allowed to warm to room temperature and stirred overnight. The resulting light brown suspension was quenched by mixing with aqueous citric acid (0.5 M, 160 mL). A light brown precipitate was observed which we attempted to dissolve by adding CHCl_3_ (150 mL) but the precipitate remained undissolved. The precipitate was filtered, washed with CHCl_3_ (50 mL) and dried in vacuo to afford an off-white solid (3.77 g). The light brown organic layer from the filtrate was separated, and the aqueous layer was extracted with CHCl_3_ (2 × 100 mL). The combined organic extracts were washed with brine (500 mL), dried over anhydrous Na_2_SO_4_, filtered, and the solvent was removed in vacuo to obtain an off-white solid. The solid was purified by trituration using a mixture of petroleum ether and CH_2_Cl_2_ (5:1, 120 mL) to afford an off-white solid (1.66 g). Total isolated yield of the title compound was 5.43 g (96%). ^1^H NMR (400 MHz, DMSO-*d*_6_) δ 7.94 (s, 1H), 7.25 (s, 1H), 6.36 (t, 1H, *J* = 1.6 Hz), 5.65 (t, 1H, *J* = 5.6 Hz), 5.42 (s, 2H), 4.72 (dd, 2H, *J* = 5.6, 1.6 Hz), 3.42 (s, 3H).

#### 5.6.4. 2-[[6-Bromo-7-*O*-(methoxymethyl)coumarin-4-yl]methoxy)]isoindoline-1,3-dione (**5**)


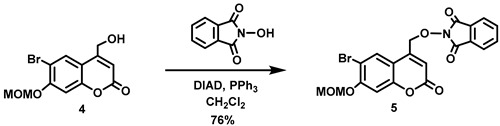


*N*-Hydroxyphthalimide (4.32 g, 26.5 mmol) and triphenylphosphine (6.93 g, 26.4 mmol) were added all at once to a stirred solution of **4** (5.56 g, 17.6 mmol) in anhydrous THF (350 mL) at 2 °C under Ar. Diisopropyl azodicarboxylate (DIAD, 5.20 mL, *d* = 1.027 g/mL at 25 °C, 26.4 mmol) was added dropwise to the reddish solution over 10 min at 0 °C. At this point, the reaction mixture turned to a pale yellow emulsion, which was stirred at 0–10 °C for 1 h before being allowed to warm to room temperature and stirred overnight. The resulting suspension was filtered to afford a white solid, which was washed with EtOH (50 mL) followed by petroleum ether (20 mL), before being dried in vacuo to give an off-white crude solid. The reddish filtrate was concentrated in vacuo, and the resulting solid was triturated with EtOH (100 mL), filtered and the resulting solid washed with EtOH (20 mL) followed by petroleum ether (20 mL) to obtain an off-white crude solid. The combined crude solid product was ground and washed sequentially with H_2_O (30 mL), EtOH (30 mL) and petroleum ether (20 mL) and dried in vacuo to afford the title compound as an off-white solid (6.21 g, 76%). Mp: 226–227 °C. ^1^H NMR (400 MHz, CDCl_3_) δ 8.28 (s, 1H), 7.91–7.85 (m, 2H), 7.83–7.76 (m, 2H), 7.18 (s, 1H), 6.51 (t, 1H, *J* = 1.2 Hz), 5.34 (s, 2H), 5.29 (d, 2H, *J* = 1.2 Hz), 3.54 (s, 3H). ^13^C NMR (101 MHz, CDCl_3_) δ 163.15, 159.86, 156.52, 154.36, 145.84, 134.92, 129.27, 128.64, 123.93, 115.09, 112.94, 108.75, 103.84, 95.17, 75.43, 56.72. HRMS (DART) *m*/*z* calculated for [M + H]^+^ 460.0026 (^79^Br) and 462.0006 (^81^Br), found 460.0024 and 462.0004.

#### 5.6.5. *O*-[[6-Bromo-7-*O*-(methoxymethyl)coumarin-4-yl]methyl]hydroxylamine (**6**)


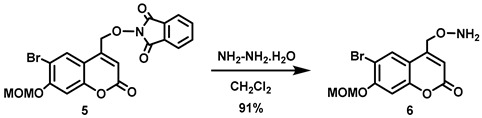


Hydrazine monohydrate (2.2 mL, *d* = 1.032 g/mL at 25 °C, 45 mmol) was added all at once to a stirred white emulsion of **5** (6.19 g, 13.5 mmol) in CH_2_Cl_2_ (350 mL) at 0 °C. The reaction mixture was stirred 0–4 °C for 30 min before being allowed to warm to room temperature and stirred overnight. The resulting suspension was filtered, and the resulting white solid was washed with CH_2_Cl_2_ (100 mL). The white solid was stirred in CH_2_Cl_2_ (100 mL) for 30 min and filtered off. The combined organic filtrates were washed with H_2_O (2 × 450 mL) and brine (500 mL), and dried over anhydrous Na_2_SO_4_ for 30 min. The drying agent was filtered off, and the solvent was removed in vacuo to afford the title compound as a pure white solid (4.02 g, 91%). Mp: 155–158 °C. ^1^H NMR (400 MHz, CDCl_3_) δ 7.80 (s, 1H), 7.16 (s, 1H), 6.37 (t, 1H, *J* = 1.2 Hz), 5.69 (s, 2H), 5.32 (s, 2H), 4.81 (d, 2H, *J* = 1.2 Hz), 3.52 (s, 3H). ^13^C NMR (101 MHz, CDCl_3_) δ 160.40, 156.14, 154.30, 150.10, 128.19, 113.24, 112.39, 108.38, 103.97, 95.13, 73.46, 56.67. HRMS (DART) *m*/*z* calculated for [M + H]^+^ 329.9972 (^79^Br) and 331.9951 (^81^Br), found 329.9971 and 331.9948.

#### 5.6.6. 1,1,1-Trifluoro-*N*-[(6-bromo-7-*O*-(methoxymethyl)coumarin-4-yl)methoxy]methanesulfonamide (**7a**)


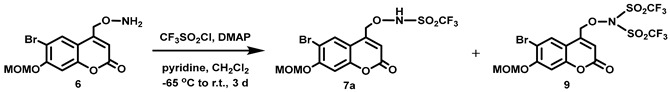


4-(*N*,*N*-Dimethylamino)pyridine (DMAP, 0.370 g, 3.03 mmol) and anhydrous pyridine (0.25 mL, *d* = 0.978 g/mL at 25 °C, 3.1 mmol) were added in one portion to a stirred colorless solution of **6** (1.01 g, 3.06 mmol) in anhydrous CH_2_Cl_2_ (100 mL) at −62 °C under Ar. A solution of CF_3_SO_2_Cl (0.48 mL, *d* = 1.583 g/mL at 25 °C, 4.5 mmol) in anhydrous CH_2_Cl_2_ (3 mL) was added dropwise over 3 min at the same temperature. The pale yellow reaction mixture was stirred at −65 °C for 2 h before being allowed to warm to room temperature and stirred overnight. An aliquot was removed and analyzed by ^1^H NMR spectroscopy, which showed some unreacted starting material. Additional CF_3_SO_2_Cl (0.24 mL, *d* = 1.583 g/mL at 25 °C, 2.3 mmol) in CH_2_Cl_2_ (2 mL) was added in one portion to the pale yellow solution at room temperature and the mixture was stirred overnight. Complete consumption of the starting material was observed by ^1^H NMR spectroscopy. The reaction mixture was passed through a short silica plug, eluting with CH_2_Cl_2_ (300 mL) followed by EtOAc (100 mL), and the solution was concentrated in vacuo to obtain a pale yellow sticky solid. The crude product was purified by flash column chromatography (silica gel, 20:80 ethyl acetate:petroleum ether) to afford the title compound as an off-white solid (0.383 g, 27%). Mp: 152–154 °C. ^19^F NMR (376 MHz, DMSO-*d*_6_) δ −73.1 (s). ^1^H NMR (400 MHz, DMSO-*d*_6_) δ 8.03 (s, 1H), 7.28 (s, 1H), 6.49 (br s, 1H), 5.44 (s, 2H), 5.21 (br s, 2H), 3.42 (s, 3H, overlap with H_2_O peak), NH peak was not observed. ^13^C NMR (101 MHz, DMSO-*d*_6_) δ 159.88, 155.84, 154.27, 148.60, 129.56, 119.94 (q, *J* = 327 Hz), 113.75, 113.11, 108.11, 104.02, 95.27, 75.55, 56.71. HRMS (DART) *m*/*z* calculated for [M + H]^+^ 461.9464 (^79^Br) and 463.9443 (^81^Br), found 461.9463 and 463.9442.

Sulfonylation of **6** with CF_3_SO_2_Cl under these reaction conditions also generate a small amount of bis-sulfonylation product **9** (0.114 g, 6%), which was isolated from the flash silica chromatography column using a mixture of 20:80 *v*/*v* ethyl acetate:petroleum ether. ^1^H NMR (400 MHz, CD_3_Cl) δ 7.84 (s, 1H), 7.18 (s, 1H), 6.46 (d, 1H, *J* = 0.4 Hz), 5.33 (s, 2H), 4.61 (d, 2H, *J* = 0.9 Hz), 3.53 (s, 3H), and ^19^F NMR (470 MHz, CD_3_Cl) δ −77.45.

#### 5.6.7. *N*-[(6-Bromo-7-*O*-(methoxymethyl)coumarin-4-yl)methoxy]methanesulfonamide (**7b**)


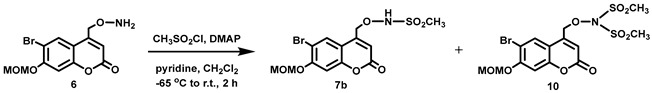


DMAP (0.093 g, 0.76 mmol) and anhydrous pyridine (0.060 mL, *d* = 0.978 g/mL at 25 °C, 0.74 mmol) were added all at once to a stirred colorless solution of **6** (0.252 g, 0.763 mmol) in anhydrous CH_2_Cl_2_ (30 mL) at room temperature under Ar. A solution of CH_3_SO_2_Cl (0.065 mL, *d* = 1.48 g/mL at 25 °C, 0.84 mmol,) in anhydrous CH_2_Cl_2_ (3 mL) was added dropwise to the solution over 5 min at room temperature. The reaction mixture was stirred at room temperature for 2 h under Ar and completion of the reaction was confirmed by ^1^H NMR spectroscopy. The solution was concentrated in vacuo and the resulting white sticky solid was purified by flash column chromatography (silica gel, 40:60 ethyl acetate:petroleum ether) to afford the title compound as a white solid (0.105 g, 34%). Mp: 190–193 °C. ^1^H NMR (400 MHz, DMSO-*d*_6_) δ 10.32 (s, 1H, NH), 8.03 (s, 1H), 7.28 (s, 1H), 6.48 (br s, 1H), 5.43 (s, 2H), 5.16 (br s, 2H), 3.43 (s, 3H), 3.07 (s, 3H). ^13^C NMR (101 MHz, DMSO-*d*_6_) δ 159.95, 155.82, 154.22, 149.73, 129.33, 113.14, 112.77, 108.14, 104.06, 95.31, 74.18, 56.72, 37.14. HRMS (DART) *m*/*z* calculated for [M + H]^+^ 407.9747 (^79^Br) and 409.9726 (^81^Br), found 407.9745 and 409.9723.

Sulfonylation of **6** with CH_3_SO_2_Cl under these reaction conditions also generated a small amount of bis-sulfonylation product **10** (0.24 g, 8%), which was isolated from the flash silica column chromatography using a mixture of 40:60 *v*/*v* ethyl acetate: petroleum ether. ^1^H NMR (400 MHz, DMSO-*d*_6_) δ 8.09 (s, 1H), 7.28 (s, 1H), 6.60 (t, 1H, *J* = 1.0 Hz, 5.44 (s, 2H), 5.39 (d, 2H, *J* = 1.1 Hz), 3.54 (s, 6H), 3.43 (s, 3H).

#### 5.6.8. *N*-[(6-Bromo-7-*O*-(methoxymethyl)coumarin-4-yl)methoxy]-2-methanesulfonyl-benzenesulfonamide (**7c**)


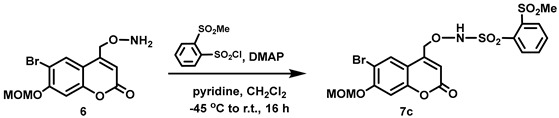


DMAP (0.131 g, 0.107 mmol) and pyridine (0.09 mL, *d* = 0.978 g/mL at 25 °C, 1 mmol) were added all at once to a stirred solution of **6** (0.354 g, 1.07 mmol) in anhydrous CH_2_Cl_2_ (50 mL) at −45 °C under Ar. A solution of (2-methanesulfonyl)benzenesulfonyl chloride (0.414 g, 1.63 mmol) in anhydrous CH_2_Cl_2_ (20 mL) was added to the resulting clear solution in one portion at −45 °C and the reaction mixture was allowed to stir at −40 °C for 30 min before being allowed to warm to room temperature and stirred for 16 h (completion of the reaction was confirmed by ^1^H NMR spectroscopy). The resulting pale yellow mixture was passed through a short silica plug using an eluent mixture of CH_2_Cl_2_ and EtOAc (4:1, 300 mL). The solvent was removed in vacuo to afford a yellow solid (0.624 g). The crude product was purified by flash column chromatography (silica gel, 2:98 MeOH:CH_2_Cl_2_) to afford the title compound as a white solid (0.353 g, 60%). Mp: 174–177 °C. ^1^H NMR (400 MHz, DMSO-*d*_6_) δ 10.39 (s, 1H, NH), 8.32–8.23 (m, 1H), 8.22–8.15 (m, 1H), 8.08–7.99 (m, 2H), 7.96 (s, 1H), 7.26 (s, 1H), 6.44 (s, 1H), 5.43 (s, 2H), 5.19 (s, 2H), 3.47 (s, 3H), 3.42 (s, 3H). ^13^C NMR (101 MHz, DMSO-*d*_6_) δ 159.90, 155.76, 154.24, 148.80, 139.48, 136.15, 135.35, 135.04, 133.10, 132.49, 129.40, 113.76, 113.30, 108.04, 104.03, 95.27, 74.76, 56.72, 44.40. HRMS (DART) *m*/*z* calculated for [M + H]^+^ 547.9679 (^79^Br) and 549.9657 (^81^Br), found 547.9678 and 549.9656.

#### 5.6.9. 1,1,1-Trifluoro-*N*-[(6-bromo-7-hydroxycoumarin-4-yl)methoxy]methanesulfonamide (**8a**)


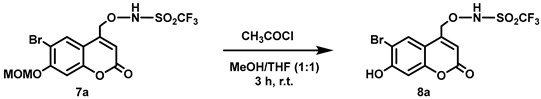


Acetyl chloride (1.8 mL, *d* = 1.104 g/mL at 25 °C, 25 mmol) was added dropwise over 5 min to a stirred solution of **7a** (0.175 g, 0.379 mmol) in anhydrous MeOH and anhydrous THF (1:1 mixture, 8 mL) at room temperature under Ar. Bubbles of HCl gas were observed during the addition. The resulting colorless clear solution was stirred at room temperature for 4 h (completion of the reaction was confirmed by ^1^H NMR analysis). The solvent was removed in vacuo to obtain an off-white solid (0.161 g). The crude solid product was purified by gravity column chromatography (silica gel, initially 2:98 MeOH:CH_2_Cl_2_ followed by 5:95 MeOH:CH_2_Cl_2_) followed by trituration using MeOH and CH_2_Cl_2_ (1:99, 2 × 5 mL) to afford the title compound as an off-white solid (0.132 g, 83%). Dec: 141–152 °C. ^19^F NMR (376 MHz, DMSO-*d*_6_) δ −75.0. ^1^H NMR (400 MHz, DMSO-*d*_6_) δ 11.38 (s, 1H, Ar-OH), 8.02 (s, 1H), 6.87 (s, 1H), 6.29 (s, 1H), 4.74 (s, 2H), NH peak was not observed. ^13^C NMR (101 MHz, DMSO-*d*_6_) δ 159.48, 157.32, 153.85, 148.12, 128.99, 119.25 (q, *J* = 328 Hz), 111.74, 110.73, 106.08, 103.02, 75.14. HRMS (DART) *m*/*z* calculated for [M + H]^+^ 417.9202 (^79^Br) and 419.9181 (^81^Br), found 417.9200 and 419.9178.

#### 5.6.10. *N*-[(6-Bromo-7-hydroxycoumarin-4-yl)methoxy]methanesulfonamide (**8b**)


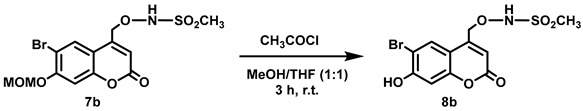


Acetyl chloride (0.80 mL, *d* = 1.104 g/mL at 25 °C, 11 mmol) was added dropwise over 3 min to a stirred solution of **7b** (0.069 g, 0.17 mmol) in an anhydrous mixture of MeOH/THF (1:1, 6 mL) at room temperature under Ar. The resulting clear solution was stirred for 3 h at room temperature before the solvent was removed in vacuo. The resulting crude white solid was triturated with CH_2_Cl_2_ to afford the title compound as a white solid (0.049 g, 80%). Dec: 187–195 °C. ^1^H NMR (400 MHz, DMSO-*d*_6_) δ 11.51 (s, 1H, Ar-OH), 10.32 (s, 1H, NH), 7.93 (s, 1H), 6.92 (s, 1H), 6.37 (s, 1H), 5.14 (s, 2H), 3.07 (s, 3H). ^13^C NMR (101 MHz, DMSO-*d*_6_) δ 160.17, 157.81, 154.36, 150.02, 129.34, 111.34, 111.29, 106.65, 103.60, 74.21, 37.11. HRMS (DART) *m*/*z* calculated for [M + H]^+^ 363.9485 (^79^Br) and 365.9464 (^81^Br), found 363.9486 and 365.9465.

#### 5.6.11. *N*-[(6-Bromo-7-hydroxycoumarin-4-yl)methoxy]-2-methanesulfonylbenzenesulfonamide (**8c**)


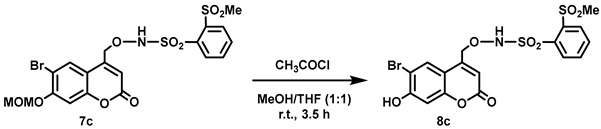


Acetyl chloride (1.8 mL, *d* = 1.104 g/mL at 25 °C, 25 mmol) was added dropwise over 5 min to a stirred suspension of **7c** (0.203 g, 0.370 mmol) in a mixture of anhydrous THF and anhydrous MeOH (1:1, 6 mL) at room temperature under Ar. The suspension turned into a clear solution after complete addition of the acetyl chloride and the reaction mixture was allowed to stir at room temperature. After 2 h, a white precipitate with reduced volume of the solvent was observed and an additional amount of anhydrous MeOH (5 mL) was added to the suspension to increase the solvent volume before stirring for an additional 1.5 h. The reaction suspension was filtered, and the resulting white solid was washed with a mixture of petroleum ether and CH_2_Cl_2_ (10:1, 11 mL) and dried in vacuo to afford a white flaky solid (0.144 g, 77%). Dec: 182–186 °C. ^1^H NMR (400 MHz, DMSO-*d*_6_) δ 11.52 (br. s, 1H, Ar-OH), 10.37 (br. s, 1H, NH), 8.31–8.25 (m, 1H), 8.23–8.15 (m, 1H), 8.08–8.00 (m, 2H), 7.87 (s, 1H), 6.89 (s, 1H), 6.32 (br s, 1H), 5.17 (br s, 2H), 3.47 (s, 3H). ^13^C NMR (101 MHz, DMSO-*d*_6_) δ 160.09, 157.79, 154.39, 148.96, 139.46, 136.03, 135.39, 135.04, 133.11, 132.56, 129.41, 112.36, 111.53, 106.58, 103.61, 74.83, 44.44. HRMS (DART) *m*/*z* calculated for [M + H]^+^ 503.9417 (^79^Br) and 505.9395 (^81^Br), found 503.9417 and 505.9396.

#### 5.6.12. Alternative Approach to Intermediate **5** via **11**


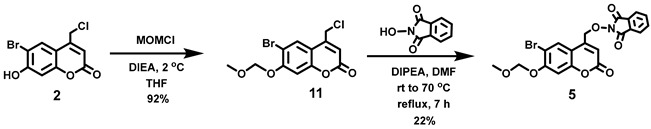


In addition, to minimize the number of synthetic steps en route to intermediate **5**, an alternative approach was explored via intermediate **11**. Compound **11** was prepared in high yield from **2** (see above for the preparation of **2**). However, S_N_2 displacement of chloride from **11** using the conjugate base of *N*-hydroxyphthalimide in the presence of triethylamine afforded **5** in only 22% yield. Significant decomposition of **5** under the reaction conditions and during subsequent silica column chromatography was observed.

#### 5.6.13. 6-Bromo-7-*O*-(methoxymethyl)-4-chloromethylcoumarin (**11**)


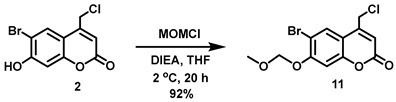


*N*,*N*-Diisopropylethylamine (DIPEA, 7.90 mL, *d* = 0.742 g/mL at 25 °C, 45.4 mmol) was added all at once to a stirred reddish solution of 2 (5.24 g, 18.1 mmol) in anhydrous THF (120 mL) at 2 °C under Ar. Upon addition, the color of the solution immediately turned yellow. Chloromethyl methyl ether (MOMCl, 3.20 mL, *d* = 1.06 g/mL at 25 °C, 42.1 mmol) was added to the yellow solution dropwise over 20 min at 1–2 °C. At this point, the solution turned a reddish color and was stirred at 1–2 °C for 30 min before stirring at room temperature overnight (20 h). After 20 h stirring, the solvent was removed in vacuo to obtain a pale yellow solid, which was re-dissolved in CHCl_3_ (180 mL). The organic solution was washed with H_2_O (3 × 200 mL) and brine (200 mL) and dried over anhydrous Na_2_SO_4_. Filtration and concentration in vacuo afforded the title compound as an off-white solid (5.547 g, 92%). ^1^H NMR (400 MHz, CDCl_3_) δ 7.83 (s, 1H), 7.17 (s, 1H), 6.45 (t, 1H, *J* = 0.8 Hz), 5.33 (s, 2H), 4.60 (d, 2H, *J* = 0.8 Hz), 3.52 (s, 3H).

#### 5.6.14. 2-[(6-Bromo-7-*O*-(methoxymethyl)coumarin-4-yl)methoxy]isoindoline-1,3-dione (**5**)


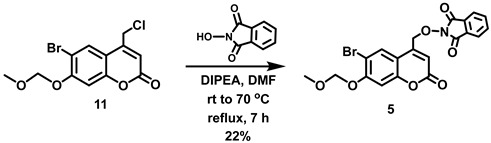


A solution of **11** (4.67 g, 14.0 mmol) in anhydrous DMF (60 mL) was added in one portion to a stirred reddish solution of *N*-hydroxyphthalimide (2.51 g, 15.4 mmol) and DIPEA (2.6 mL, *d* = 0.742 g/mL at 25 °C, 15.12 mmol) in anhydrous DMF (30 mL) at room temperature under Ar. The reaction mixture was heated to 70 °C with stirring until the reaction had proceeded to completion (7 h, checked by TLC). The reaction mixture was allowed to cool to room temperature, diluted with EtOAc (200 mL) and washed with H_2_O (200 mL) to remove substantial amounts of the DMF reaction solvent. A white suspension was observed in between the layers that was not soluble even upon addition of EtOAc (50 mL). The organic layer along with the white suspension was separated from the aqueous layer. Filtration of the organic layer led to the isolation of the white solid, which was identified as phthalimide by ^1^H NMR spectroscopy. The aqueous layer was extracted with EtOAc (2 × 150 mL) and the combined organic extracts were washed with water (2 × 400 mL) and dried over MgSO_4_. Filtration and concentration in vacuo afforded a reddish solid (5.15 g). The crude product was purified by trituration with EtOH to afford a pale yellow solid containing minor impurities. The pale yellow solid was triturated with EtOAc followed by washing with EtOH to afford the title compound as an off-white solid (1.415 g, 22%) that contained a small impurity (~6%). ^1^H NMR (400 MHz, CDCl_3_) δ 8.28 (s, 1H), 7.89–7.87 (m, 2H), 7.81–7.79 (m, 2H), 7.17 (s, 1H), 6.51 (t, 1H, *J* = 1.2 Hz), 5.33 (s, 2H), 5.29 (d, 2H, *J* = 1.2 Hz), 3.54 (s, 3H).

#### 5.6.15. 6-Bromo-7-hydroxycoumarin-4-aldoxime (**13**)


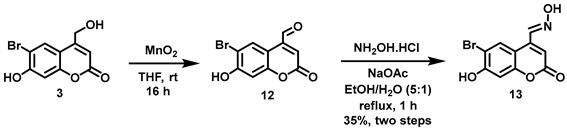


MnO_2_ (4.18 g, 4.81 mmol) was added all at once to a stirred solution of **3** (0.197 g, 0.727 mmol) in anhydrous THF (20 mL) under Ar. The resulting suspension was stirred at room temperature overnight before removing the black solid by filtration through Celite. The Celite was washed with CH_2_Cl_2_ (20 mL) and the filtrate was concentrated in vacuo to afford crude aldehyde **12** as a yellow sticky solid (0.161 g). The crude product was used in the next step without further purification. ^1^H NMR (400 MHz, DMSO-*d*_6_) δ 11.66 (s, 1H), 10.07 (s, 1H), 8.60 (s, 1H), 7.00 (s, 1H), 6.95 (s, 1H). ^13^C NMR (101 MHz, DMSO-*d*_6_) δ 193.53, 160.01, 157.48, 154.62, 142.46, 129.37, 121.46, 108.25, 106.53, 103.22.

A solution of NH_2_OH.HCl (0.046 g, 0.66 mmol) and NaOAc (0.054 mg, 0.66 mmol) in H_2_O (3 mL) was added all at once to a stirred solution of crude **12** in EtOH (15 mL). The pale yellow clear solution was heated to reflux. After 20 min the solution appeared cloudy. After refluxing for 1 h, the reaction mixture was allowed to cool to room temperature before adding H_2_O (20 mL). Since no crystallization was achieved even after placing the mixture in an ice-salt-water bath for 30 min, the solution was filtered, and the filtrate was left open inside the hood for two days to permit slow solvent evaporation. Yellow needle-like crystals were formed and were collected by filtration, washed with H_2_O (15 mL), and dried in vacuo to afford yellow flaky needles (0.054 g), characterized as the title compound but containing a minor impurity. The crude crystals were further purified by recrystallization using a mixture (1:5) of EtOH and water to afford the title compound as yellow flaky crystalline needles (0.037 g, 18%, in two steps). ^1^H NMR (400 MHz, DMSO-*d*_6_) δ 12.44 (s, 1H), 8.71 (s, 1H), 8.37 (s, 1H), 6.91 (s, 1H), 6.52 (s, 1H). N-OH peak was not observed. ^13^C NMR (101 MHz, DMSO-*d*_6_) δ 160.31, 157.77, 154.81, 147.65, 143.67, 131.42, 113.95, 110.16, 106.56, 103.75.

## Figures and Tables

**Table 1:** Effect of solvent ratio on the photoproducts derived from the *N*-hydroxysulfonamide moiety for photodecomposition of **8b** (1.0 mM).

Percentage of Photoproducts					
Solvent Ratio, % *v*/*v* (30 mM Phosphate Buffer, pH 7.0/CD_3_CN)	CH_3_SO_2_NH_2_	CH_3_SO_2_NHOH	CH_3_SO_2_^−^	CH_3_SO_3_^−^	Unknown
00/100	82	8	3	3	4
60/40	93	1	0	0	2
90/10	79	5	2	9	5

**Table 2:** Effect of the pH of the aqueous component of the solvent on the photoproducts derived from the *N*-hydroxysulfonamide moiety for photodecomposition of **8a** (1.0 mM) in 10:90 *v*/*v* CD_3_CN:aqueous buffer (30 mM). Note that small peaks (≤2%) from secondary products were also observed at pH 2.1 and 10.0.

pH	Percentages of Photoproducts	
	CF_3_SO_2_^−^	CF_3_SO_2_NH_2_
2.1 ^a^	4	96
5.0 ^b^	19	81
7.0 ^a^	20	80
10.0 ^c^	19	81

^a^ Phosphate buffer, ^b^ acetate buffer, and ^c^ carbonate buffer.

**Table 3:** Effect of the pH of the aqueous component of the solvent on the photoproducts derived from the *N*-hydroxysulfonamide for photodecomposition for **8b** (1.0 mM) in 10:90 *v*/*v* CD_3_CN:aqueous buffer (30 mM).

pH	Percentage of Photoproducts				
	CH_3_SO_2_NH_2_	CH_3_SO_2_NHOH	CH_3_SO_2_^−^	CH_3_SO_3_^−^	Unknown
2.1 ^a^	78	8	2	6	6
5.0 ^b^	78	8	2	5	7
7.0 ^c^	81	11	4	4	0
10.0 ^d^	40	4	48	8	0
10.7 ^e^	39	5	54	2	0

Aqueous component of solution: ^a,c^ phosphate buffer, ^b^ acetate buffer, and ^d,e^ carbonate buffer.

## Data Availability

The original contributions presented in the study are included in this article/Supplementary Materials. Further inquiries can be directed to the corresponding authors.

## Supplementary Materials

The following supporting information can be downloaded at: https://www.mdpi.com/article/10.3390/molecules29163918/s1, Determination of molar extinction coefficients; photostability data; spectra showing the photoisomerization of (*E*)-BHC-oxime to (*Z*)-BHC-oxime; phosphine trapping of HNO for **8a**; determination of p*K*a values, photoproducts as a function of pH; the effect of the excitation wavelength on the photoproducts and photoproduct quantum yields; NMR and HRMS spectra for synthetic intermediates and products.
