# Supplementary material for: Developing Photoactive Coumarin-Caged N-Hydroxysulfonamides for Generation of Nitroxyl (HNO)
Source: Molecules. 2024 Aug 19;29(16):3918. doi: 10.3390/molecules29163918 (PMC11356963; doi:10.3390/molecules29163918)
Supplement: Supplementary file 1 [file molecules-29-03918-s001.zip › molecules-3112336-supplementary.pdf]

Supporting Information

Developing Photoactive Coumarin-caged *N*-Hydroxysulfonamides for Generation of Nitroxyl (HNO)

Mohammad S. Rahman, Vinay Bharadwaj, Anau K. H. S. Lautaha, Paul Sampson, Nicola E. Brasch and Alexander J. Seed

Table of Contents

	Page
Section S1. Determination of the molar extinction coefficients for **8a** and **8b**	**S2**
Section S2. Studies on the photochemical stability of **8a** and **8b** under normal lab light conditions	**S2**
Section S3. Stability of 8a in the presence of red light	**S3**
Section S4. Isomerization of *(E)*-BHC-oxime(**13a**) to *(Z)*-BHC-oxime (**13b**) upon irradiation	**S4**
Section S5. Detection of HNO upon photolysis of **8a** using a phosphine trap	**S5**
Section S6. Determining the pK_a values for **8a**	**S8**
Section S7. Determining the pK_a of 6-bromo-7-hydroxy-4-methylcoumarin	**S10**
Section S8. Determining the pK_a value of **8a** using UV-Vis spectroscopy	**S11**
Section S9. Determining the pK_a values for **8b** using NMR spectroscopy	**S11**
Section S10. Determination of the photoproducts for **8a** as a function of pH	**S13**
Section S11. Determination of the photoproducts for **8b** as a function of pH	**S17**
Section S12. Effect of the excitation wavelength on the photoproducts	**S22**
Section S13. Determination of photoproduct quantum yields	**S22**
Section S14. NMR and HRMS spectra for fully characterized synthetic intermediates and products	**S24**

Section S1. Determination of the molar extinction coefficients for 8a and 8b

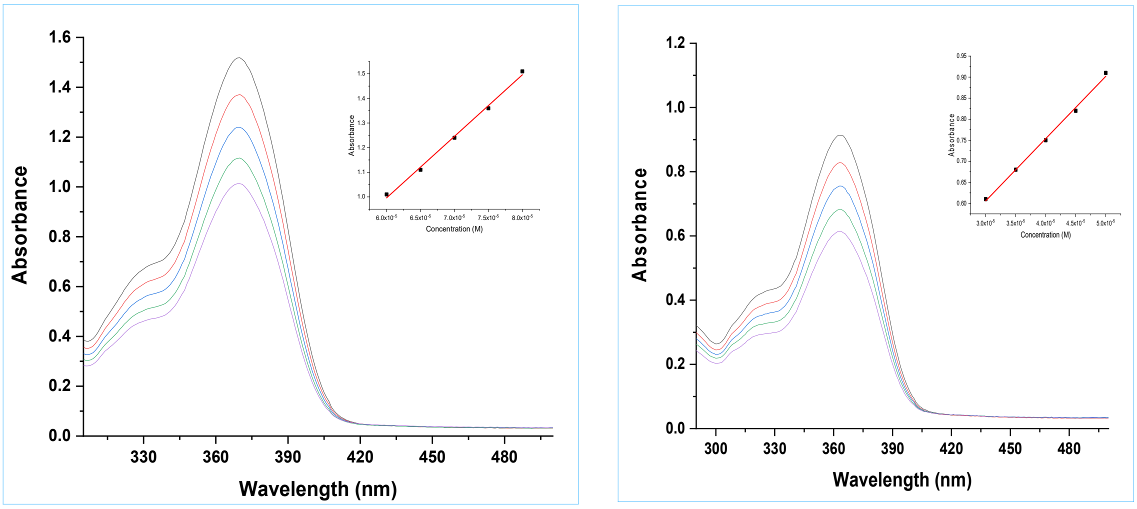

(a) (b)

Figure S1. UV-vis spectrum of (a) **8a** (60.0, 65.0, 70.0, 75.0 and 80.0 μM) and (b) **8b** (30.0, 35.0, 40.0, 45.0 and 50.0 μM) in a mixture of water and CH_3CN (92:8 v/v) at a range of concentrations at 25.0 °C. Inset: Plot of absorbance at (a) 370 nm and (b) 366 nm versus concentration**.** The best fit of the data to a line passing through the origin gives a molar extinction coefficient, $\varepsilon_{370\ nm}$ = 2.50 × 10^4 M^{-1} cm^{-1}, $\varepsilon_{366\ nm}$ = 1.42 × 10^4 M^{-1} cm^{-1}. Errors are estimated to be 2-3%.

Section S2. Studies on the photochemical stability of 8a and 8b under ambient lab light conditions

The photostability of **8a** (1.0 mM) in aerobic CD_3CN was investigated by recording the ^{19}F NMR spectrum ~10 min after preparing the sample and ~12 h later, in the presence of fluorescent light in the lab. After 12 h, 58% **8a** had decomposed to give $CF_3SO_2NH_2$ (33%), $CF_3SO_2^-$ (17%) and $CF_3SO_3^-$ (8%), Figure S2.

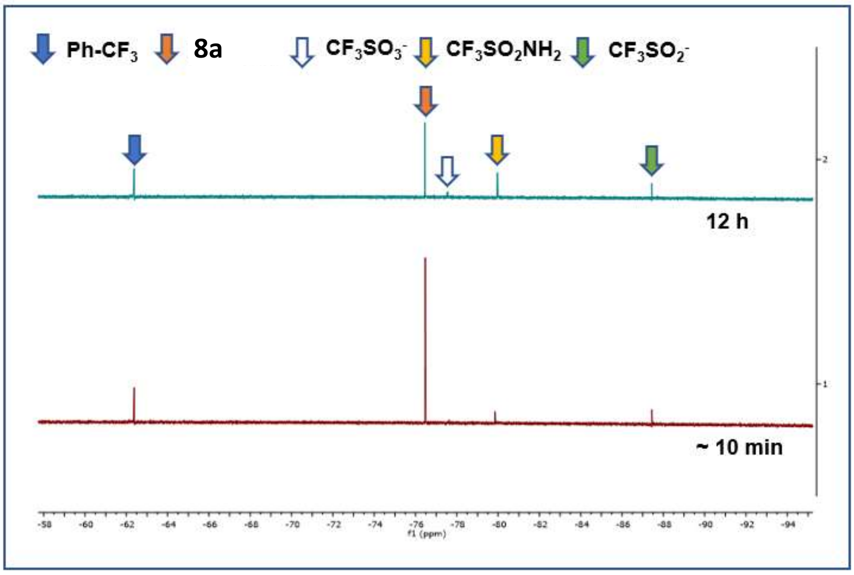

Figure S2. ^{19}F NMR spectra of **8a** (1.0 mM) in CD_3CN after ~10 min and 12 h following sample preparation under aerobic conditions. The solution was exposed to fluorescent light in the lab.

The photostability of **8b** was examined in the presence of fluorescent light in the lab by recording the ^{1}H NMR spectrum immediately after sample preparation and also after 12 h. No photodecomposition was observed (spectra not shown).

Section S3. Stability of 8a in the presence of red light

8a was found to be stable in the presence of red light (λ ≈ 620-680 nm, 40 Watts maximum). UV-Vis, ^{19}F and ^{1}H NMR spectra were recorded ~10 min after preparing a sample of **8a** in CH_3CN (UV-Vis) or CD_3CN (NMR) under red light conditions and again 240 min later (Figure S3 and Figure S4). Negligible decomposition was observed. Therefore, all the photolysis experiments were carried out in the presence of red light, including the sample preparation.

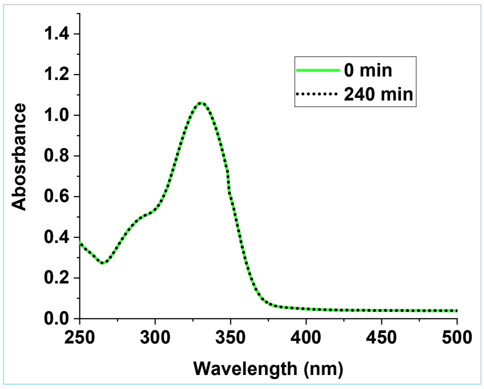

Figure S3. UV-Vis spectrum of **8a** (90 μM) in CH_3CN was recorded after ~ 10 min and 240 min later. The sample was exposed to red light. There was no change in the UV-Vis spectrum after irradiation. Spectra were recorded at 25 °C.

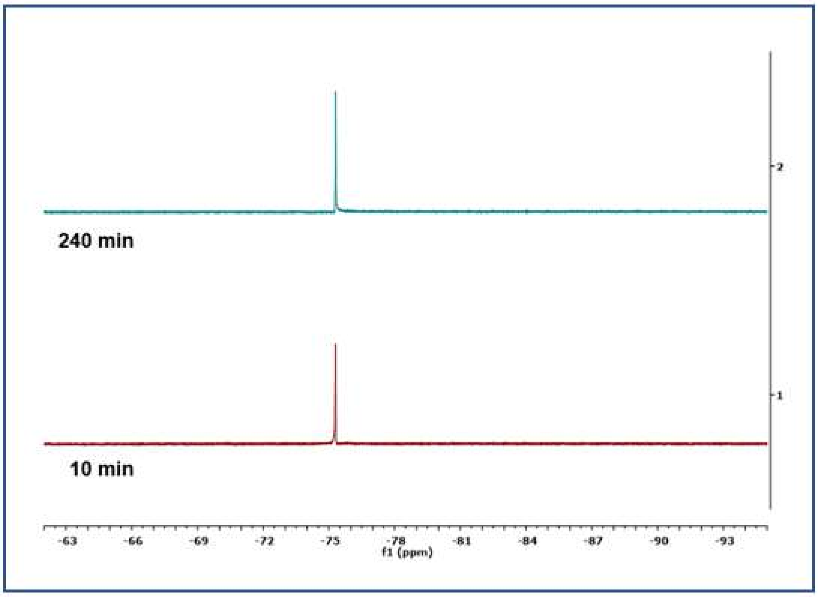

(a)

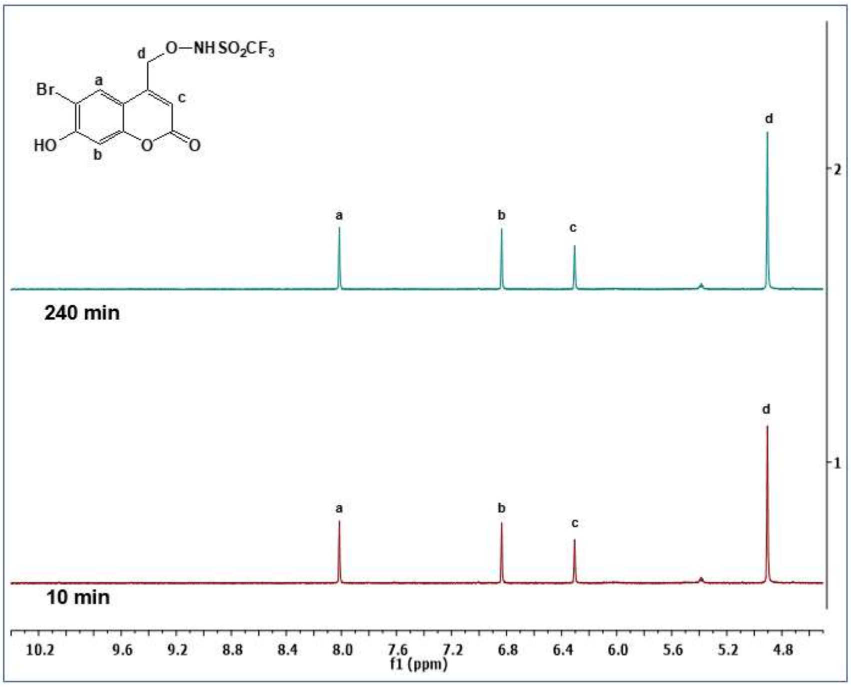

(b)

Figure S4. (a) ^{19}F and (b) ^{1}H NMR spectra of **8a** (1.0 mM) in CD_3CN recorded after ~10 min under red light conditions and again 240 min later. No decomposition occurred. The impurity at 5.37 ppm (^{1}H NMR) was from CD_3CN (solvent). Assignments for the aromatic protons of **8a** are also shown.

Section S4. Isomerization of *(E)*-BHC-oxime (13a) to *(Z)*-BHC-oxime (13b) upon irradiation

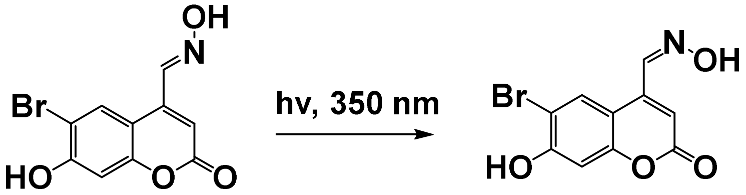

***(E)*-BHC-oxime (13a)** ***(Z)*-BHC-oxime (13b)**

Figure S5 shows the ^{1}H NMR spectrum of *(E)*-BHC-oxime before and after irradiation in CD_3CN using a Rayonet photoreactor (350 nm bulbs). Peaks at 8.63, 8.23, 6.91 and 6.36 ppm were assigned to **13a**. After 0.40 min, additional peaks appeared at 7.62, 7.71, 6.91, and 6.55 ppm. These chemical shifts were assigned to **13b**. The photoconversion of **13a** to **13b** under these conditions was 50%.

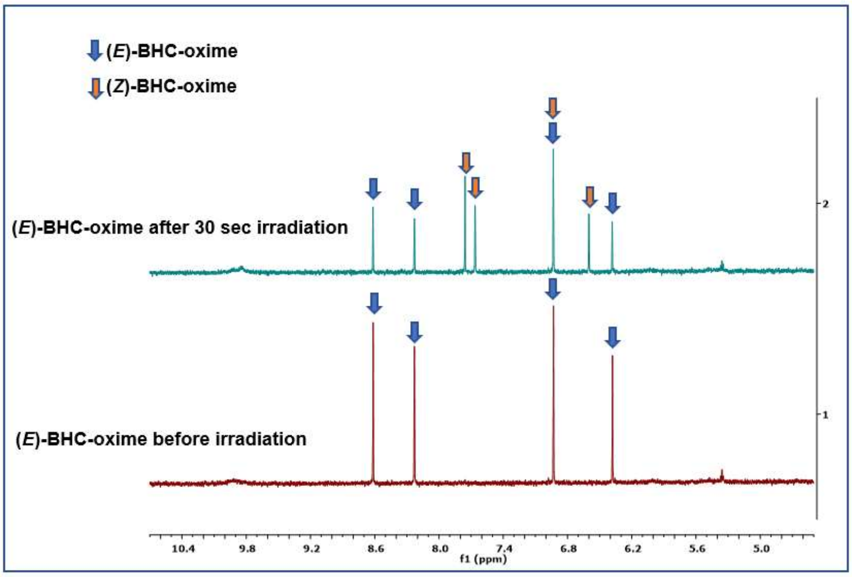

Figure S5. ^{1}H NMR spectra of *(E)*-BHC-oxime (**13a**) before irradiation and after 0.40 min irradiation in CD_3CN using a Rayonet photoreactor (350 nm). The impurity at 5.37 ppm was from the CD_3CN solvent.

Section S5. Detection of HNO upon photolysis of 8a using a phosphine trap

Triphenylphosphine derivatives have been widely used for both qualitative and quantitative detection of HNO from various HNO donor molecules [42-49]. Release of HNO from BHC-$ONHSO_2CF_3$ (**8a**) was investigated by trapping with a common triphenylphosphine detector (phosphine **S1**, Figure S6) under anaerobic conditions. Based on the literature, one equivalent of HNO should react with two equivalents of phosphine, leading to the formation of one equivalent of phosphine oxide **S2** and one equivalent of diagnostic HNO detection compound(s) including phosphine aza-ylide **S3** and/or phosphine oxide amide **S4** (Figure S6).

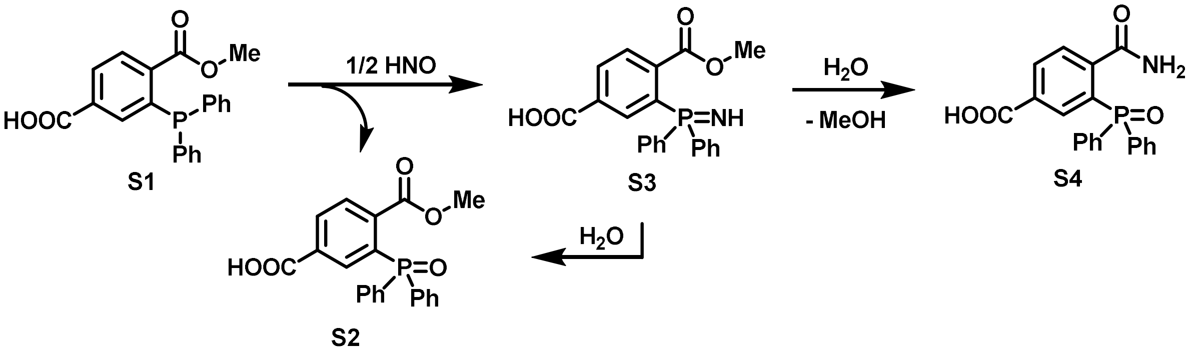

Figure S6. Reaction between HNO and phosphine **S1**.

Therefore, release of HNO from the photolysis of **8a** was analyzed using two equivalents of phosphine **S1**. A sample solution of BHCM-$ONHSO_2CF_3$ (**8a**) (1.0 mM) and phosphine **S1** (2.0 mM) was prepared using an 80:20 v/v mixture of CD_3CN and phosphate buffer (5.0 mM, pH 7.0) in an air-tight quartz NMR tube inside a glovebox. The sample solution was monitored by ^{1}H, ^{19}F and ^{31}P NMR spectroscopy before and after irradiation for 9 min under 350 nm light using a Rayonet photoreactor (8 bulbs,

4W). Complete photolysis of **8a** was observed by ^{19}F NMR spectroscopy with release of $CF_3SO_2^-$ (89%, -88.15 ppm) and $CF_3SO_2NH_2$ (11%, -80.47 ppm) (Figure S7).

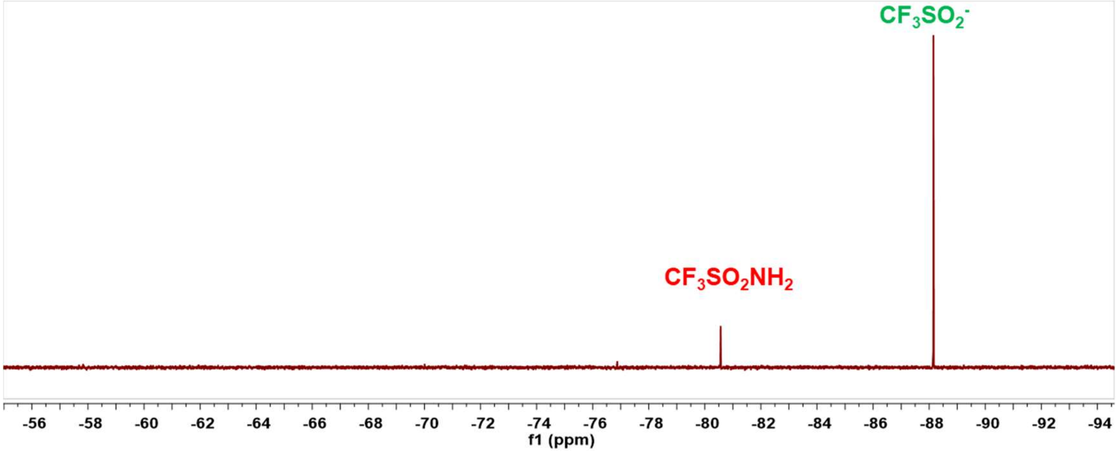

Figure S7. ^{19}F NMR spectrum of BHCM-ONHSO$_2$CF$_3$ (**8a**) in the presence of phosphine **S1** in an 80:20 mixture of CD_3CN and phosphate buffer solution (5.0 mM, pH = 7.0) after irradiation for 9 min under 350 nm light using a Rayonet photoreactor (8 bulbs, 4W)

The ^{31}P NMR spectrum of the photolysis of **8a** in the presence of phosphine **S1** indicated the formation of diagnostic phosphine oxide **S2** (33.16 ppm), phosphine aza-ylide **S3** (40.42 ppm) and phosphine amide **S4** (33.94 ppm), Figure S8. The appearance of phosphine aza-ylide **S3** and phosphine amide **S4** were a clear indication of the trapping of HNO released from the photolysis of HNO donor **8a**. Note that BHC-oxime was also observed in the product mixture by ^1H NMR spectroscopy (spectrum not shown); hence the phosphine trap does not trap all of the (H)NO generated. It was not surprising to see a smaller amount of phosphine aza-ylide **S3** and phosphine amide **S4** compared to phosphine oxide **S2**, since aza-ylide **S3** can undergo hydrolysis to phosphine oxide **S2** under photolytic conditions.[4, 9] It was also observed that a significant amount of phosphine **S1** remained unreacted. No interaction between BHC-oxime **13a** and phosphine **S1** was observed under ambient light and after irradiation for 9 min under similar photolytic conditions (spectrum 3, Figure S8). According to the literature, stoichiometric detection of HNO is frequently not observed when using such phosphine detectors; quantitation of HNO release was found to be unreliable under certain conditions [23,24,50]. Therefore, while these experiments offer convincing *qualitative* evidence for HNO release on photolysis of **8a**, reliable quantitation of HNO release from these studies is not possible.

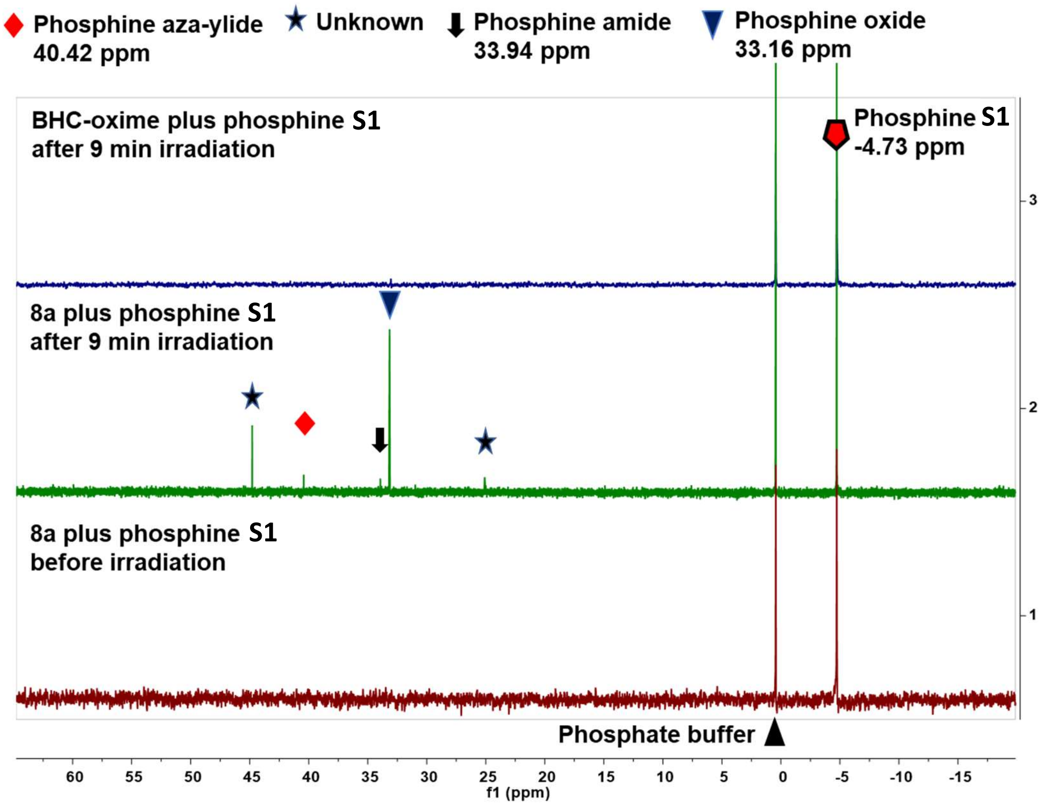

Figure S8. ^{31}P NMR spectra of BHCM-$ONHSO_2CF_3$ (**8a**) in the presence of phosphine **S1** in an anaerobic 80:20 v/v mixture of CD_3CN and phosphate buffer solution (5.0 mM, pH 7.0) before (spectrum 1) and after irradiation for 9 min under 350 nm light using a Rayonet photoreactor (8 bulbs, 4W) (spectrum 2). A control experiment involving the photolysis of a mixture of BHC-oxime **13a** and phosphine **S1** under the same conditions is also shown (spectrum 3).

Interestingly, the ^{31}P NMR spectrum of the photoproducts obtained upon aerobic photolysis of **8a** in presence of phosphine **S1** indicated extensive conversion of phosphine **S1** with the exclusive formation of phosphine oxide (33.08 ppm) (Spectra 1 and 2, Figure S9). No phosphine aza-ylide, phosphine amide or any unknown compounds were observed. This suggests that, in the presence of O_2, phosphine aza-ylide, phosphine amide and the two unknown compounds were hydrolyzed or oxidized to phosphine oxide. A control photolysis experiment with phosphine **S1** in the absence of **8a** under aerobic conditions showed significant conversion of phosphine **S1** to phosphine oxide (Spectra 3 and 4, Figure S9), whereas no such conversion was observed under anaerobic conditions. Therefore, trapping of HNO released from photoactivatable HNO donors using a phosphine detector under aerobic conditions may not be effective due to competing facile photooxidation of the phosphine **S1**.

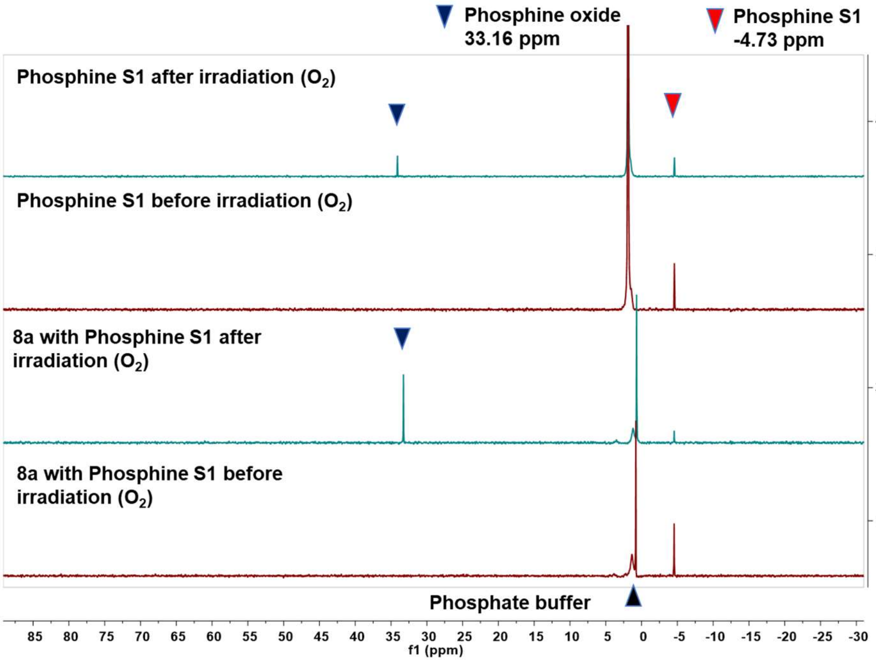

Figure S9. ^{31}P NMR spectra of BHCM-ONHSO$_2$CF$_3$ (**8a**) in the presence of phosphine **S1** (Spectra 1 and 2), and phosphine **S1** control (Spectra 3 and 4) in an aerobic 80:20 mixture of CD_3CN and phosphate buffer solution (0.10 M, pH = 7.0) before and after irradiation for 10 min under 350 nm light using a Rayonet photoreactor (8 bulbs, 4W)

Section S6. Determining the p*K*$_a$ values for 8a

A control experiment was carried out to check that **8a** does not decompose during the pK_a determination experiments. **8a** was dissolved in a mixture of CH_3CN and D_2O (8:92 v/v CH_3CN: D_2O). The pD was adjusted to 2.35 and the ^{1}H NMR spectrum was recorded. The pD was increased to pD 5.70 (adjusted by adding a small volume of NaOH dissolved in D_2O) and the ^{1}H NMR spectrum was again recorded. The solution was then adjusted back to pD 2.25 (addition of a small volume of 40% HCl in D_2O) and the ^{1}H NMR spectrum was recorded for the last time. The chemical shifts of **8a** were identical within experimental error to their original values, and no decomposition was observed (spectra not shown).

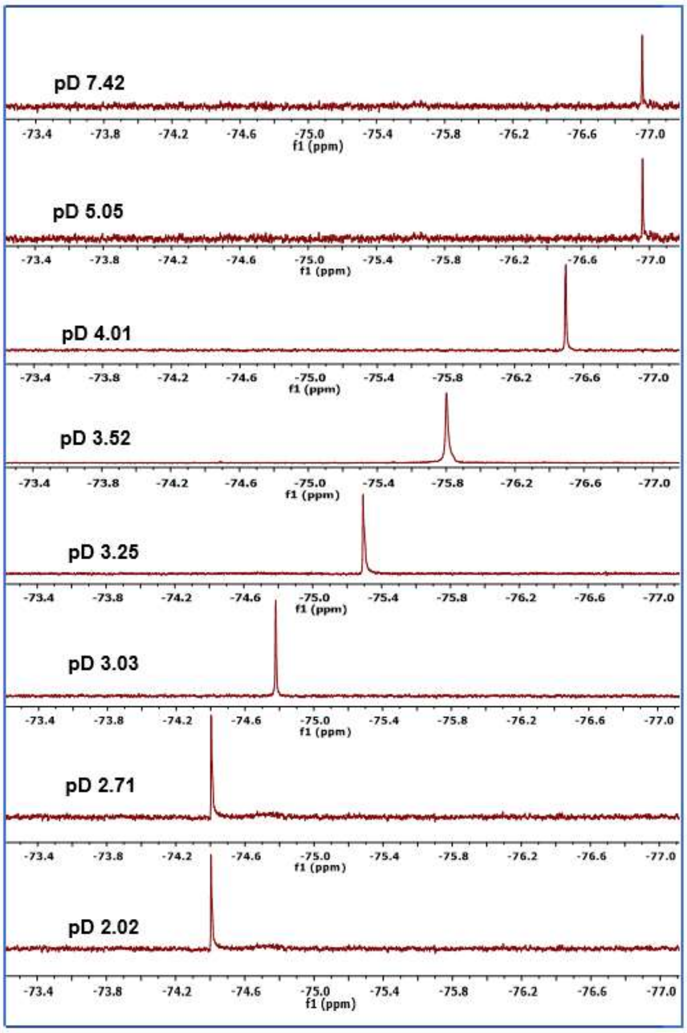

Figure S10. Selected ^{19}F NMR spectra of **8a** as a function of pD, in D_2O with 8% v/v CH_3CN, *I* = 1.0 M, CF_3SO_3Na.The spectra are referenced to α,α,α-trifluorotoluene (-62.9 ppm).

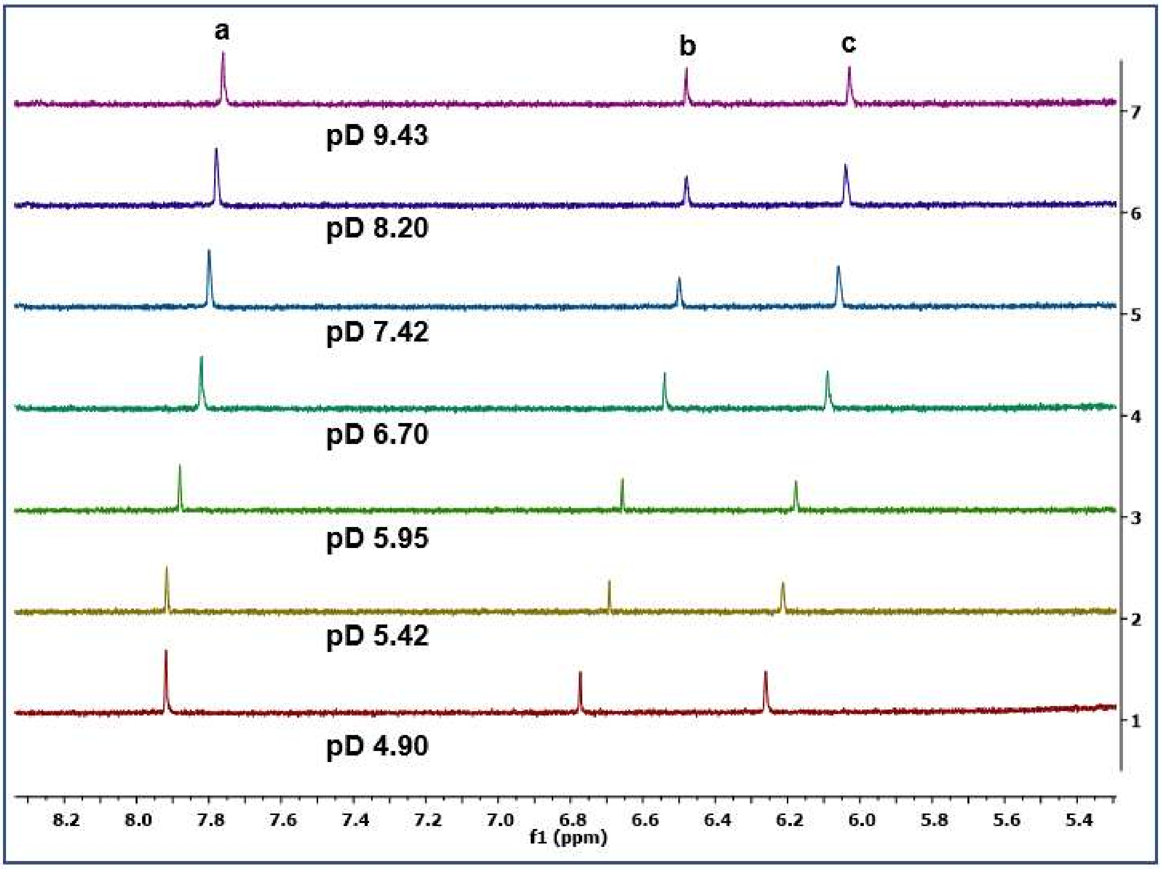

Figure S11. Selected ^{1}H NMR spectra for **8a** as a function of pD, in D_2O with 8% v/v CH_3CN, I = 1.0 M, CF_3SO_3Na. TSP was used as an external reference.

Section S7. Determining the pK_a of 6-bromo-7-hydroxy-4-methylcoumarin

A pK_a titration experiment was conducted for 6-bromo-7-hydroxy-4-methylcoumarin (equation (S.1)), so this value could be compared to the pK_a for the 7-hydroxy substituent of **8a** and **8b.**

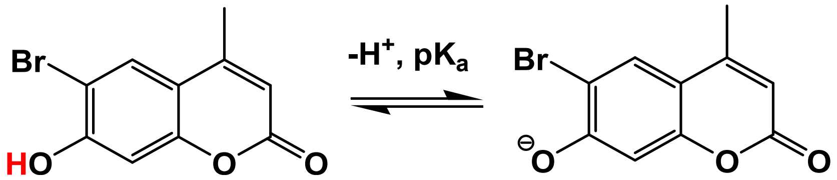

(S.1)

A UV-Vis spectroscopic titration of 6-bromo-7-hydroxy-4-methylcoumarin (110 μM, 30 mL solution) was conducted in a mixture of 8:92 v/v CH_3CN : H_2O, using a flow set up. The absorbance at 367 nm was plotted as a function of pH (Figure S12(a)). Data were fitted to equation (2) (see main article), giving pK_a = 6.91 ± 0.03 (Figure S12(b)).

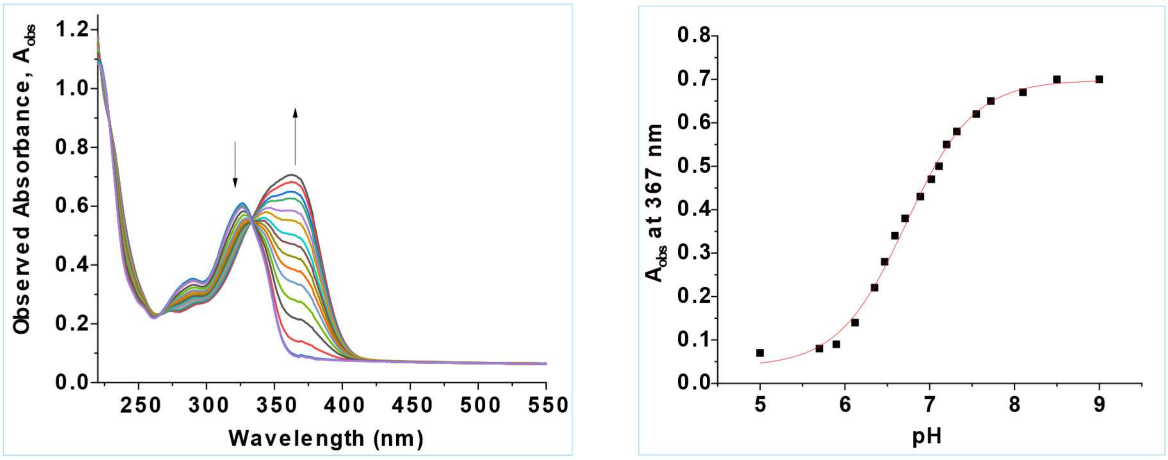

(a) (b)

Figure S12. (a) UV-Vis spectra of 6-bromo-7-hydroxy-4-methylcoumarin (110 µM) as a function of pH from pH 4.4-8.7 (8:92 v/v CH_3CN : H_2O). (b) Plot of absorbance at 367 nm versus pH. The data has been fitted to equation (2), giving pK_a = 6.91 ± 0.03, A_{HA}= 0.71 ± 0.03 and A_{A-} = 0.05 ± 0.03.

Section S8. Determining the p*K*a value of 8a using UV-Vis spectroscopy

Prior to carrying out a UV-Vis titration experiment, a control experiment was conducted to ensure that **8a** does not decompose during the pK_a determination experiments using UV-Vis spectroscopy. The UV-Vis spectrum of **8a** was recorded in a mixture of CH_3CN and H_2O (8:92 v/v CH_3CN : H_2O) at pH 2.01. The solution pH was increased to pH 9.01 (by adding a small volume of aqueous NaOH) and the UV-Vis spectrum was recorded again. The solution was then adjusted back to pH 2.10 (addition of a small volume of aqueous HCl). The final absorbance spectrum of **8a** was identical to the original UV-Vis spectrum recorded within experimental error, and no decomposition was observed (spectra not shown). This is in agreement with the control experiments conducted using ^{19}F NMR spectroscopy (see Section S6).

Section S9. Determining the p*K*a values for 8b using NMR spectroscopy

A control experiment was conducted to check that **8b** does not decompose during an NMR titration experiment. The ^{1}H NMR spectrum of **8b** (1.0 mM) was recorded in 8:92 v/v CH_3CN : D_2O. The pD was then increased to 11.03 and the ^{1}H NMR spectrum again recorded, with no observed decomposition. The pD was decreased back to pD 2.52. The ^{1}H NMR spectrum showed that no decomposition had occurred (spectra not shown). At pD values lower than 2.5 or higher than 11.0, **8b** was thermally unstable.

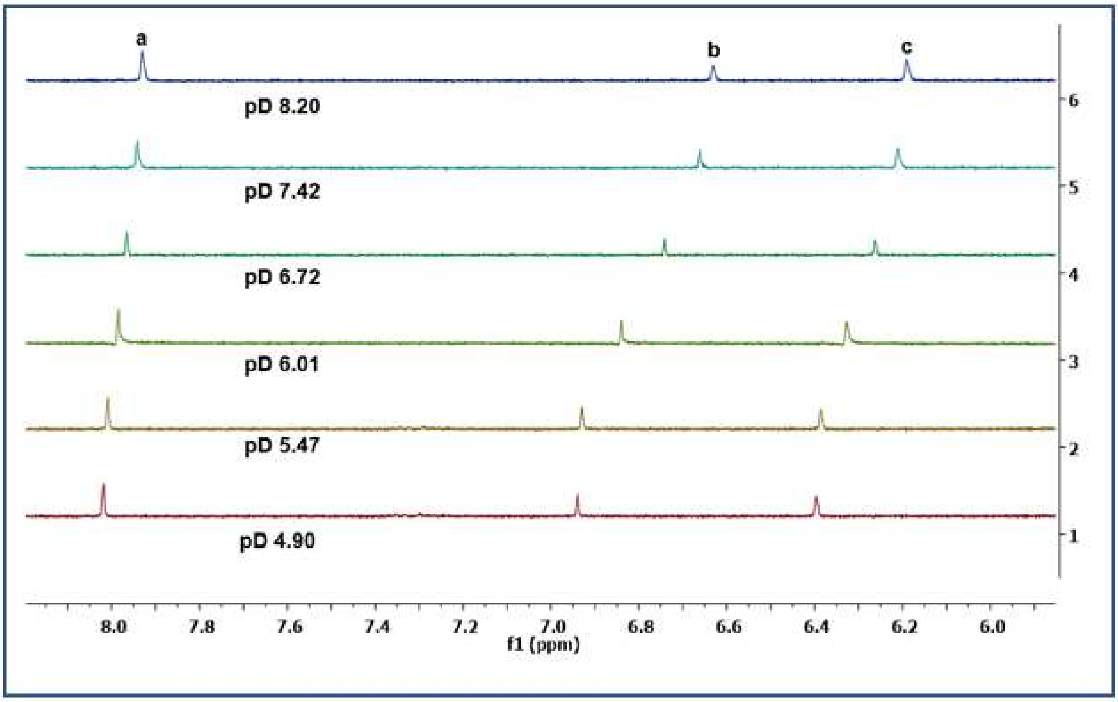

Figure S13. Selected ^{1}H NMR spectra for **8b** as a function of pD in D_2O with 8% v/v CH_3CN, I = 1.0 M, CF_3SO_3Na. TSP was used as an external reference.

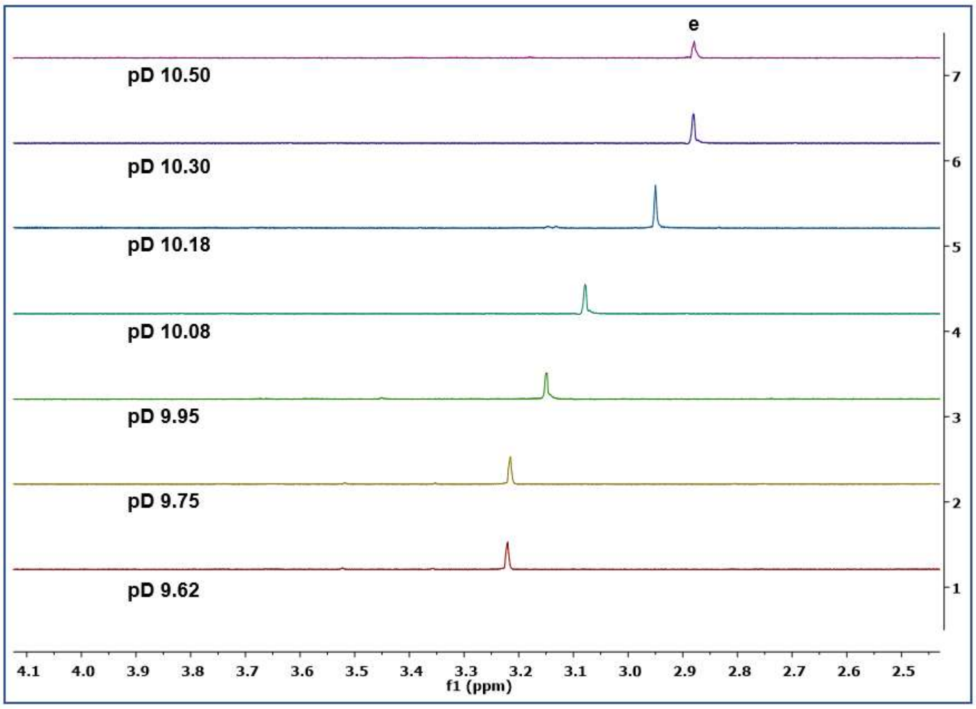

Figure S14. ^{1}H NMR spectra showing the chemical shift of the CH_3 (**e**) protons of **8b** as a function of pD in D_2O with 8% v/v CH_3CN, I = 1.0 M, $NaCF_3SO_3$.

Section S10. Determination of the photoproducts for 8a as a function of pH

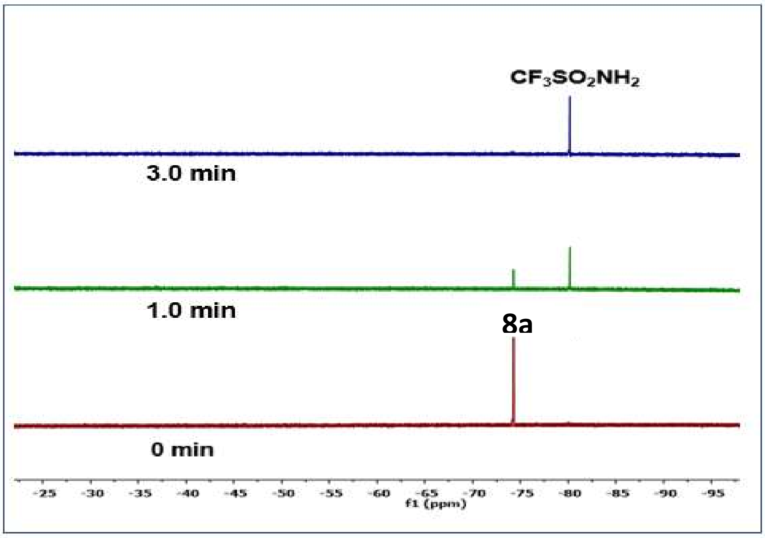

(a)

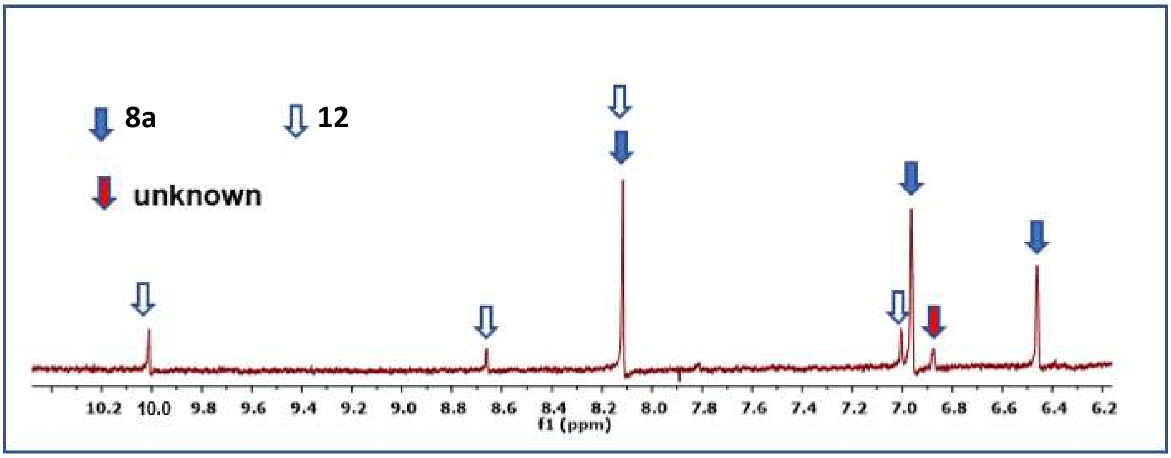

(b)

Figure S15. (a) ^{19}F NMR spectra for the photodecomposition of **8a** (1.0 mM) in 10:90 v/v CD_3CN : phosphate buffer (30 mM, pH 2.1) before irradiation, after 1.0 min, and after 3.0 min total irradiation (350 nm). $CF_3SO_2NH_2$ is observed at -80.2 ppm. (b) ^1H NMR spectrum of the same sample after 1.0 min irradiation. Approximately 30% decomposition has occurred. The chemical shifts at 10.01, 8.66, 8.11 and 7.00 ppm are assigned to BHC-CHO (**12**). An unknown peak at 6.87 ppm is observed. To establish whether this unknown peak originated from the photodecomposition of BHC-CHO, the ^1H NMR spectrum of BHC-CHO was recorded after irradiation for 1.0 min. BHC-CHO was found to be photostable within this timeframe.

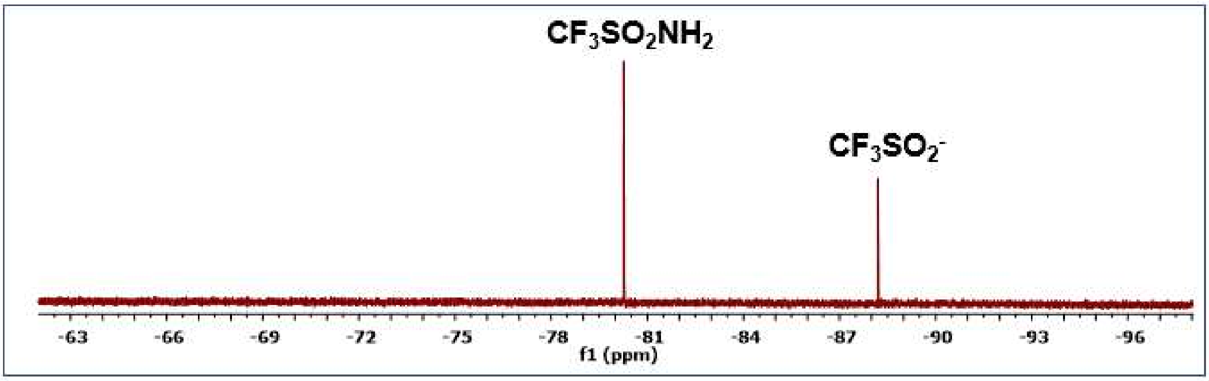

(a)

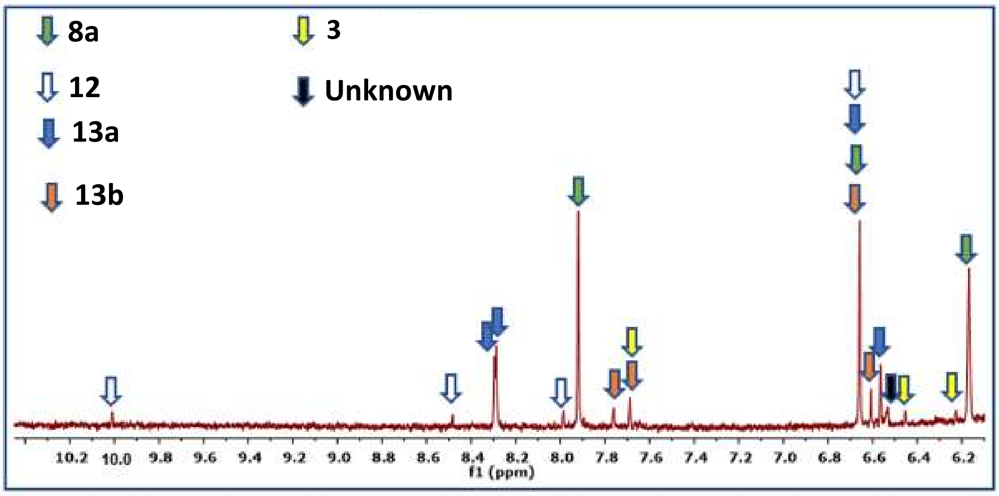

(b)

Figure S16. (a) ^{19}F NMR spectrum of **8a** (1.0 mM) in a mixture of acetate buffer (pH 5.0, 30.0 mM) and CD_3CN (90:10 v/v) after 3.0 min irradiation. (b) ^{1}H NMR spectrum of the same solution after irradiating for 0.40 min.

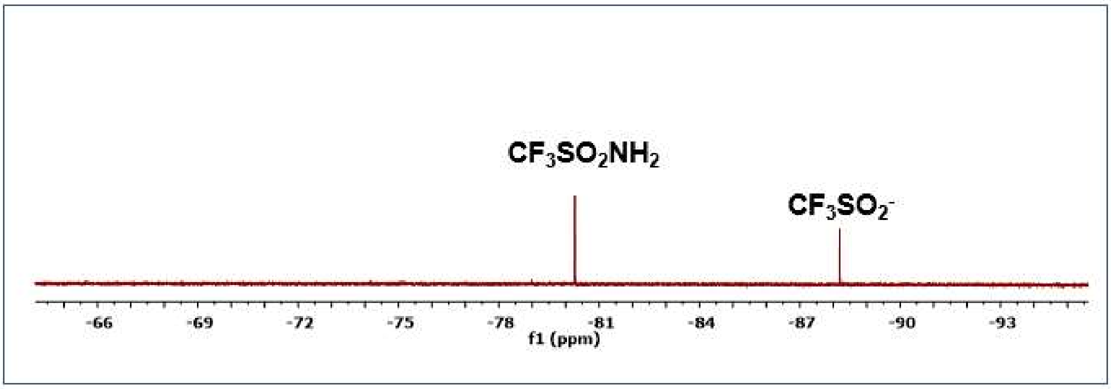

(a)

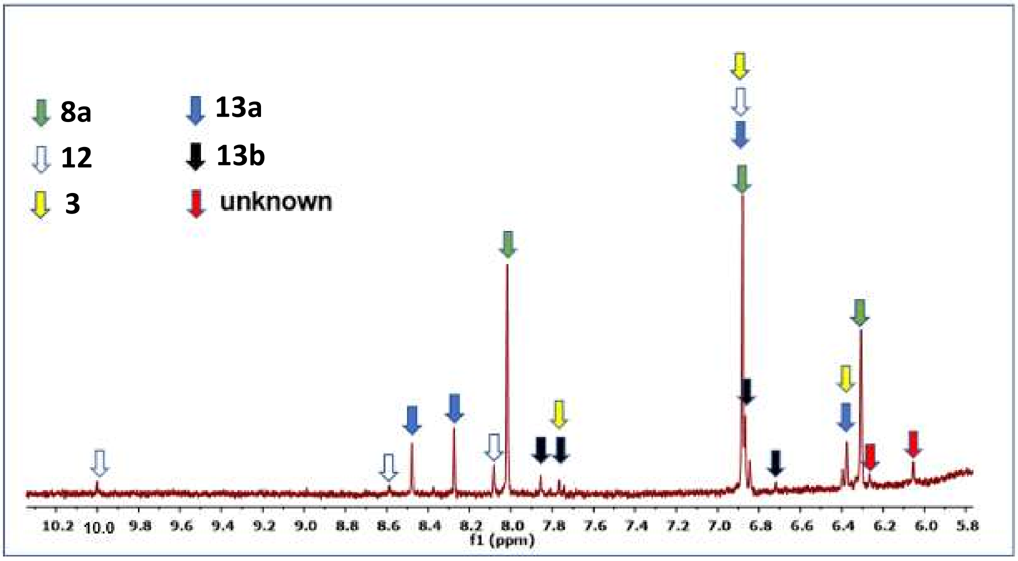

(b)

Figure S17. (a) ^{19}F NMR spectrum of **8a** (1.0 mM) in a mixture of phosphate buffer (pH 7.0, 30.0 mM) and CD_3CN (90:10 v/v) after 3.8 min irradiation. (b) ^{1}H NMR spectrum of the same solution after irradiating for 0.40 min.

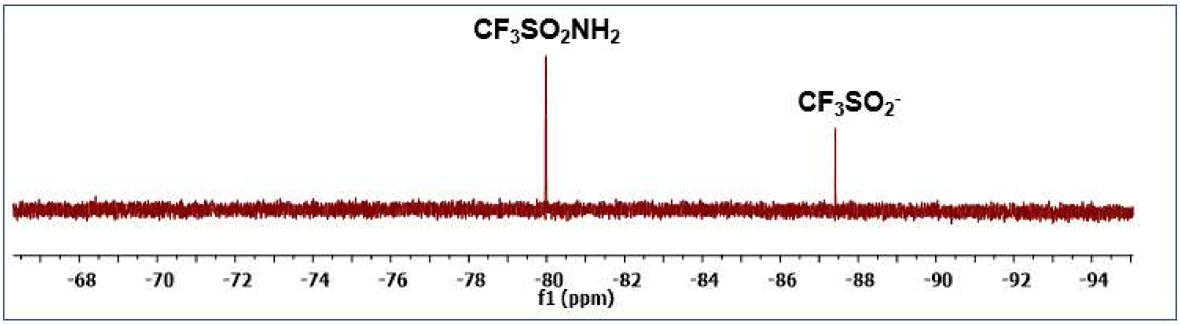

(a)

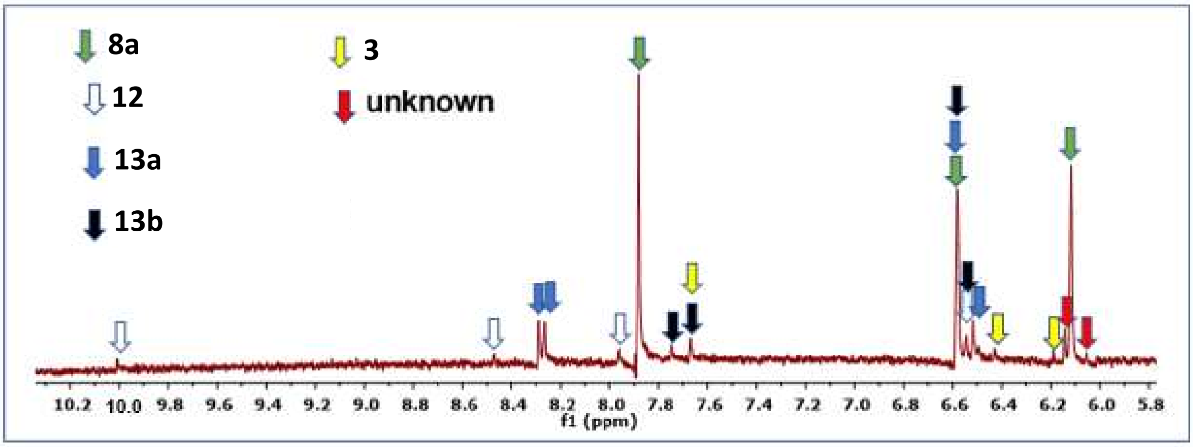

(b)

Figure S18. (a) ^{19}F NMR spectrum of **8a** (1.0 mM) in a mixture of carbonate buffer (pH 10.0, 30.0 mM) and CD_3CN (90:10, v/v) after 3.5 min irradiation. (b) ^{1}H NMR spectrum of the same solution after irradiating for 0.40 min.

Table S1. Chemical shifts of (*E*)-BHC-oxime (**13a**) and (*Z*)-BHC-oxime (**13b**) in 10:90 v/v CD_3CN : aqueous buffer (30 mM; acetate buffer (pH 5.0), phosphate buffer (pH 7.0) and carbonate buffer (pH 10.0)). The labeling scheme is also shown.

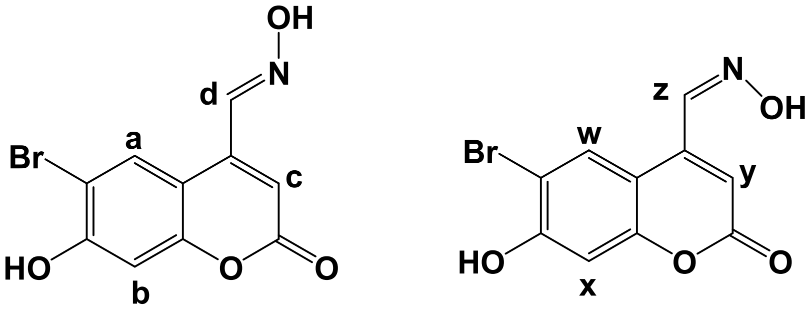

pH	Chemical shifts (ppm) of Photoproducts							
	(*E*)-BHC-oxime (**13a**) protons				(Z)-BHC-oxime (**13b**) protons			
	**a**	**b**	**c**	**d**	**w**	**x**	**y**	**z**
5.0	8.48	8.27	6.88	6.38	7.85	7.76	6.88	6.71
7.0	8.28	8.27	6.63	6.57	7.73	7.67	6.63	6.59
10.0	8.28	8.26	6.59	5.53	7.74	7.66	6.58	6.54

Table S2. Chemical shifts of BHC-CHO (**12**) and BHCM-OH (**3**) in 10:90 v/v CD_3CN : aqueous buffer (30 mM; acetate buffer (pH 5.0), phosphate buffer (pH 7.0) and carbonate buffer (pH 10.0)). The CH_2 protons from BHCM-OH overlap with the solvent signal. The labeling scheme is also shown.

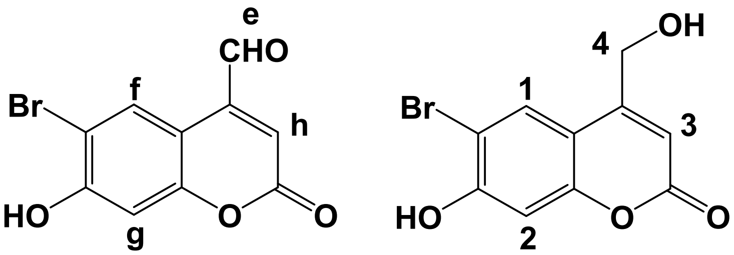

pH	Chemical shifts (ppm) of Photoproducts							
	BHC-CHO (**12**) protons				BHCM-OH (**3**) protons			
	**e**	**f**	**g**	**h**	**1**	**2**	**3**	**4**
5.0	10.01	8.58	8.07	6.88	7.76	6.88	6.38	-
7.0	10.04	8.47	7.97	6.62	7.68	6.45	6.22	-
10.0	10.00	8.47	7.96	6.54	7.66	6.45	6.18	-

Section S11. Determination of the photoproducts for 8b as a function of pH

Figure S19(a) shows the ^{1}H NMR spectrum in the 2.15-3.25 ppm region after irradiating a solution of **8b** in 90:10 v/v phosphate buffer, pH 2.5 : CD_3CN. The percentages of $CH_3SO_2NH_2$, CH_3SO_2NHOH, $CH_3SO_2^-$ and $CH_3SO_3^-$ are 78%, 8%, 2% and 6%, respectively. Hence the main mechanism of photoproduct formation is via O-N bond cleavage. A small amount of an unknown species was also observed (6%).

Figure S19(b) shows the aromatic region of the ^{1}H NMR spectrum after a short photolysis. Peaks at 10.01, 8.49, 8.00 and 6.62 ppm were assigned to BHC-CHO (**12**). Peaks at 7.69, 6.45 and 6.23 ppm were assigned to BHCM-OH (**3**). Two unassigned peaks were observed at 6.18 and 6.05 ppm.

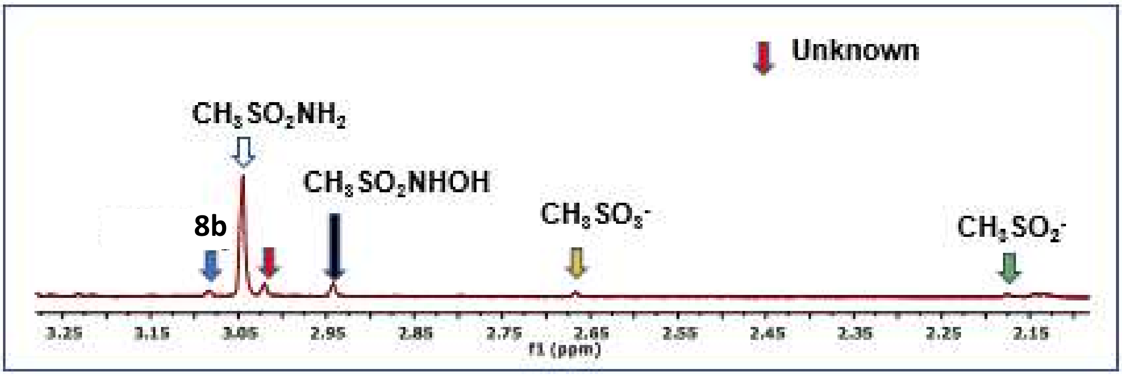

(a)

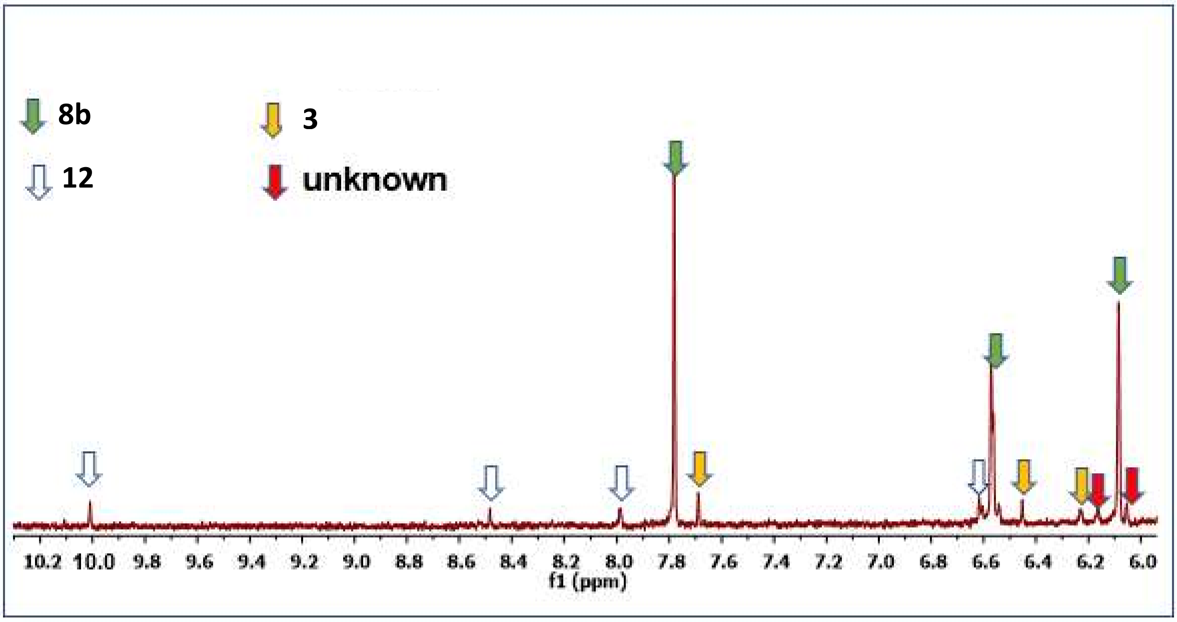

(b)

Figure S19 (a) ^{1}H NMR spectrum in the 2.15-3.25 ppm region for a solution of **8b** (1.0 mM) in a mixture of phosphate buffer (pH 2.5, 30.0 mM) and CD_3CN (90:10, v/v) after 3.0 min irradiation. (b) The aromatic region of the ^{1}H NMR spectrum for the same solution after 0.20 min.

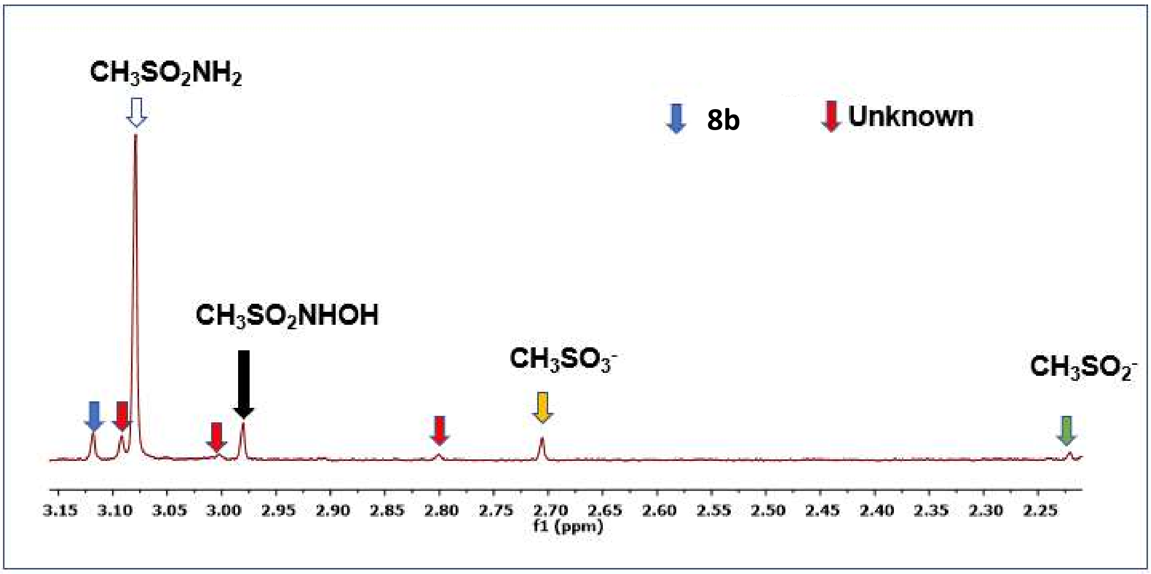

(a)

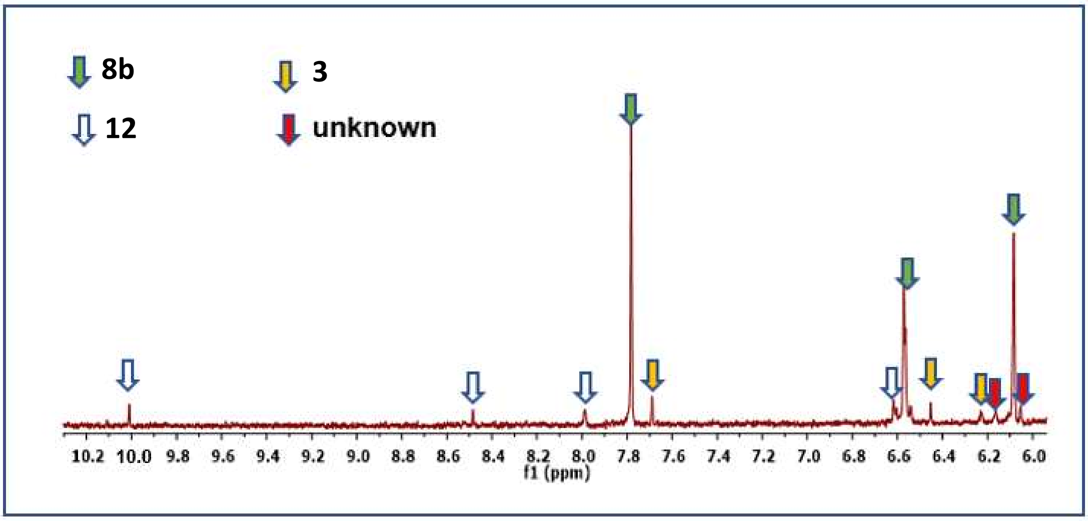

(b)

Figure S20 (a) ^{1}H NMR spectrum for a solution of **8b** (1.0 mM) in a mixture of acetate buffer (pH 5.0, 30.0 mM) and CD_3CN (90:10, v/v) after 3.0 min irradiation. (b) The aromatic region of the ^{1}H NMR spectrum for same solution after 0.20 min.

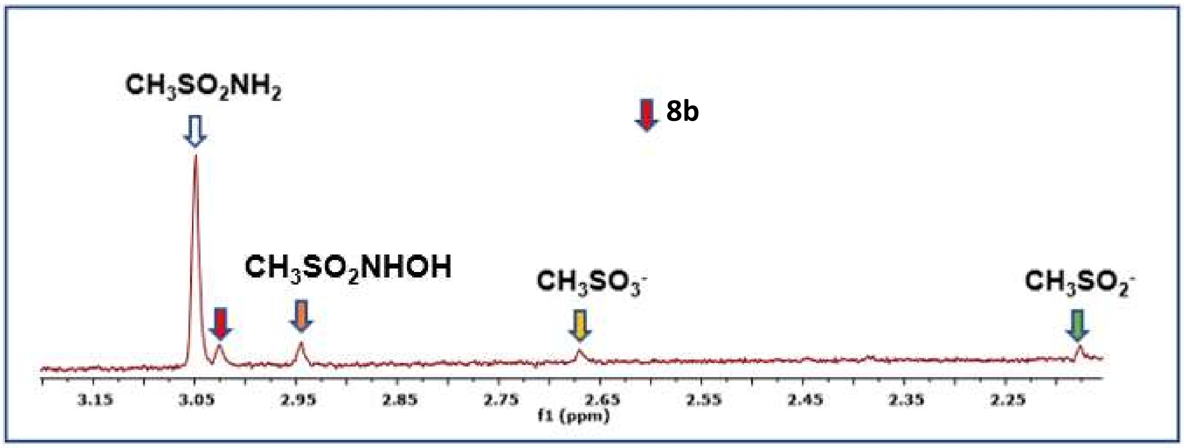

(a)

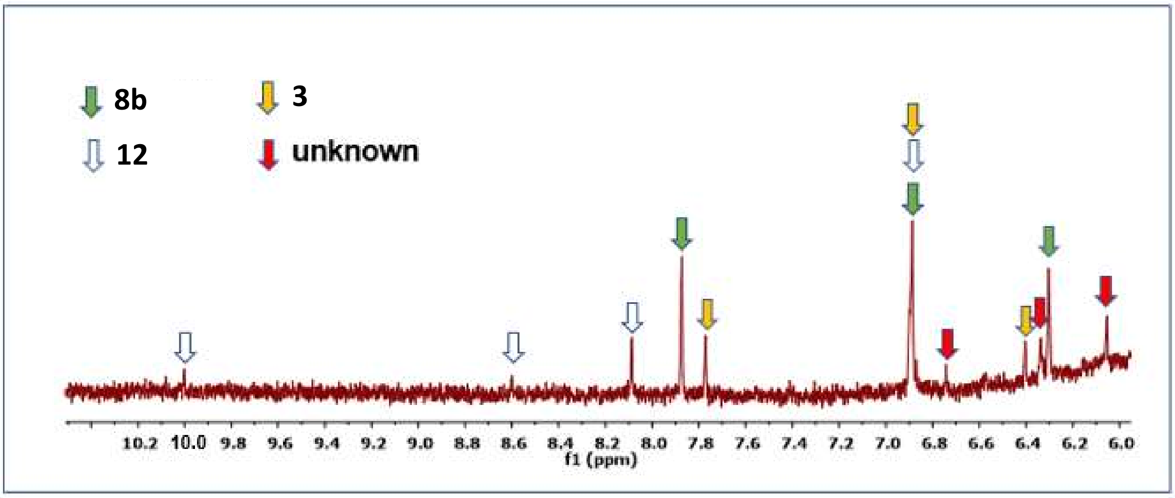

(b)

Figure S21 (a) ^{1}H NMR spectrum in the 2.15-3.15 ppm region for a solution of **8b** (1.0 mM) in a mixture of phosphate buffer (pH 7.0, 30.0 mM) and CD_3CN (90:10, v/v) after 5.0 min irradiation. (b) The aromatic region of ^{1}H NMR spectrum for the same solution after 0.20 min irradiation.

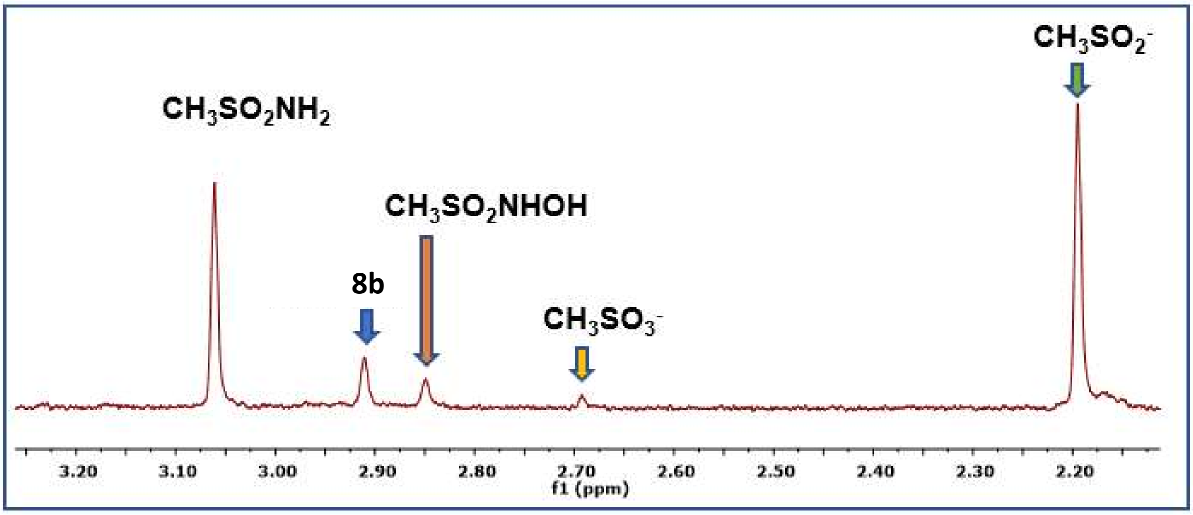

(a)

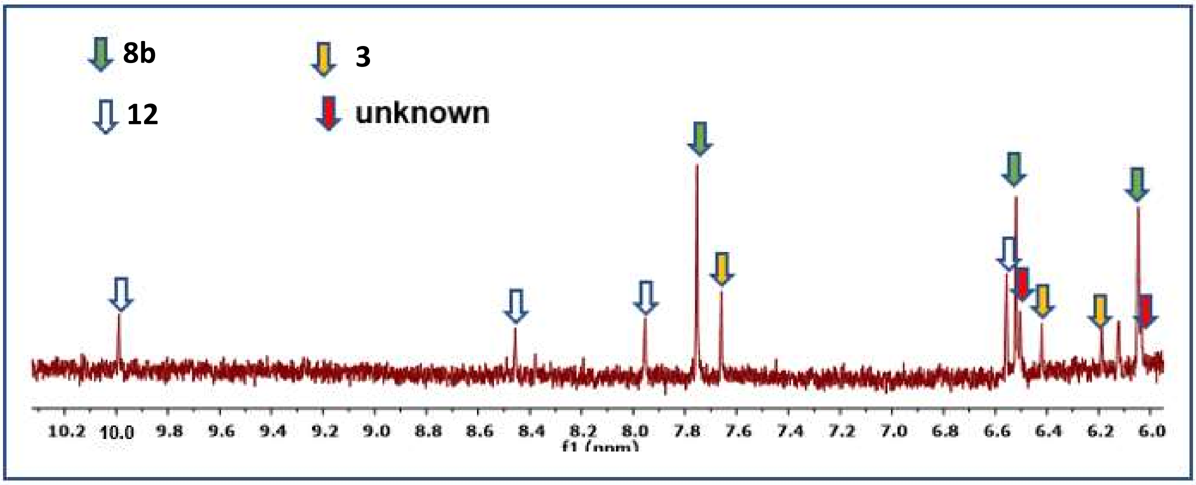

(b)

Figure S22 (a) ^{1}H NMR spectrum in the 2.20-3.20 ppm region for a solution of **8b** (1.0 mM) in a mixture of carbonate buffer (pH 10.0, 30.0 mM) and CD_3CN (90:10, v/v) after 5 min irradiation. (b) The aromatic region of ^{1}H NMR spectrum for the same solution after 0.20 min irradiation.

Section S12. Effect of the excitation wavelength on the photoproducts

Table S3. Effect of the excitation wavelength on the photoproducts derived from *N*-hydroxysulfonamide upon irradiation of **8a** or **8b** (1.0 mM) in a mixture of phosphate buffer (30 mM, pH 7.0) and CD_3CN (40:60, v/v).

compound	λ_{exc}	Photoproducts
**8a**	270	$CF_3SO_2NH_2$ (40%) and $CF_3SO_2^-$ (60%)
**8a**	355	$CF_3SO_2NH_2$ (38%) and $CF_3SO_2^-$ (62%)
**8b**	270	CH_3SO_2NHOH (7%), $CF_3SO_2NH_2$ (78%), $CH_3SO_2^-$ (4%), $CH_3SO_3^-$ (3%) and unknown (8%)
**8b**	355	CH_3SO_2NHOH (8%), $CF_3SO_2NH_2$ (77%), $CH_3SO_2^-$ (3%), $CH_3SO_3^-$ (5%) and unknown (7%)

Section S13. Determination of photoproduct quantum yields

The photoproduct quantum yields for **8a** and **8b** at 313 nm were determined in CH_3OH by actinometry, using the isomerisation of *trans*-azobenzene to its *cis* isomer as a reference compound (Φ = 0.14 at 313 nm, in CH_3OH [57]). The percentage of *trans*-azobenzene (6.61×10^{-5} M) converted to *cis*-azobenzene upon irradiation was followed by UV-Vis spectroscopy, whereas the photodecomposition of **8a** (1.00 mM) and **8b** (1.00 mM) was followed by ^{19}F or ^{1}H NMR spectroscopy. All the experiments were carried out on the same day. Figure S23(a) shows a plot of the number of moles of *cis*-azobenzene as a function of total irradiation time. The slope is $(4.96 \pm 0.03) \times 10^{-9}$ mol min^{-1}. Figure S23(b) and Figure S23(c) are plots of the total number of moles of photoproduct as a function of total irradiation time for **8a** and **8b**, respectively. The slope values for **8a** and **8b** are $(7.62 \pm 0.02) \times 10^{-9}$ mol min^{-1} and $(5.12 \pm 0.03) \times 10^{-9}$ mol min^{-1}, respectively. The slopes were substituted into equation (S1) to obtain photoproduct quantum yields.

$$\phi\,(\text{BHC-CF}_3 \text{ or BHC-CH}_3) = \frac{\text{slope (BHC-CF}_3 \text{ or BHC-CH}_3\text{) / Absorbance (BHC-CF}_3 \text{ or BHC-CH}_3\text{)}}{\text{Slope (reference) / Absorbance (reference)}} \times \phi\,(\text{reference}) \qquad \text{(S1)}$$

The absorbance of the solutions (1.00 mM) at the excitation wavelength were calculated using the molar extinction coefficient of each reactant in CH_3OH at 313 nm and were $(7.50 \pm 0.04) \times 10^3$ M^{-1} cm^{-1} (**8a**) and $(2.40 \pm 0.03) \times 10^3$ M^{-1} cm^{-1} (**8b**). The photoproduct quantum yields were 0.038 **(8a)** and 0.079 **(8b)**.

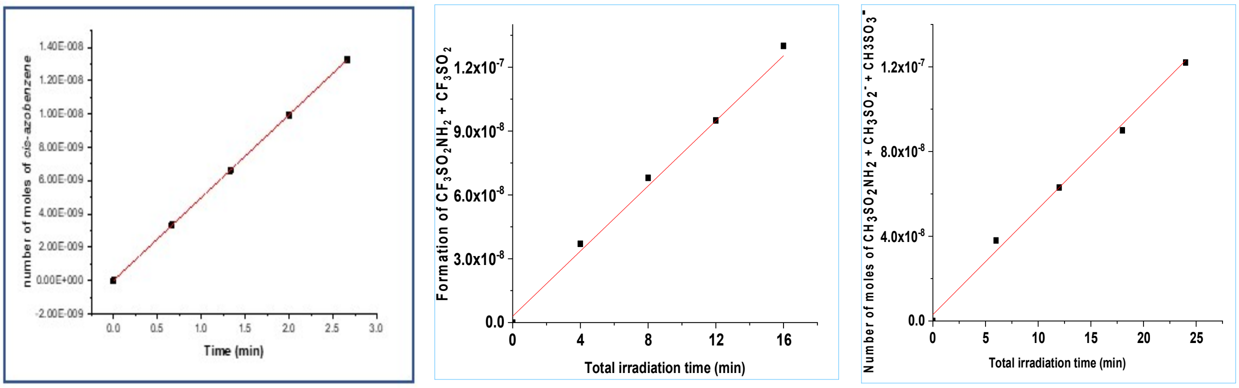

(a) (b) (c)

Figure S23. (a) Plot of the number of moles of *cis*-azobenzene vs total irradiation time (min). The slope was (4.96 ± 0.03) × 10^{-9} mol min^{-1}, and the absorbance of *trans*-azobenzene before irradiation was 1.32. (b) Plot of the number of moles of the photoproducts ($CF_3SO_2NH_2$ + $CF_3SO_2^-$) versus total irradiation time (min) obtained from the photolysis of **8a** (1.00 mM, 313 nm) in CD_3OD. The best fit of the data to a straight line gives a slope of (7.62 ± 0.02) × 10^{-9} mol min^{-1}. (c) Plot of the number of moles of the photoproducts ($CH_3SO_2NH_2$ + CH_3SO_2NHOH) versus total irradiation time (min) obtained from the photolysis of **8b** (1.00 mM, 313 nm) in CD_3OD. The best fit of the data to a straight line gives a slope of (5.12 ± 0.03) × 10^{-9} mol min^{-1}.

Section S14. NMR and HRMS spectra for fully characterized synthetic intermediates and products.

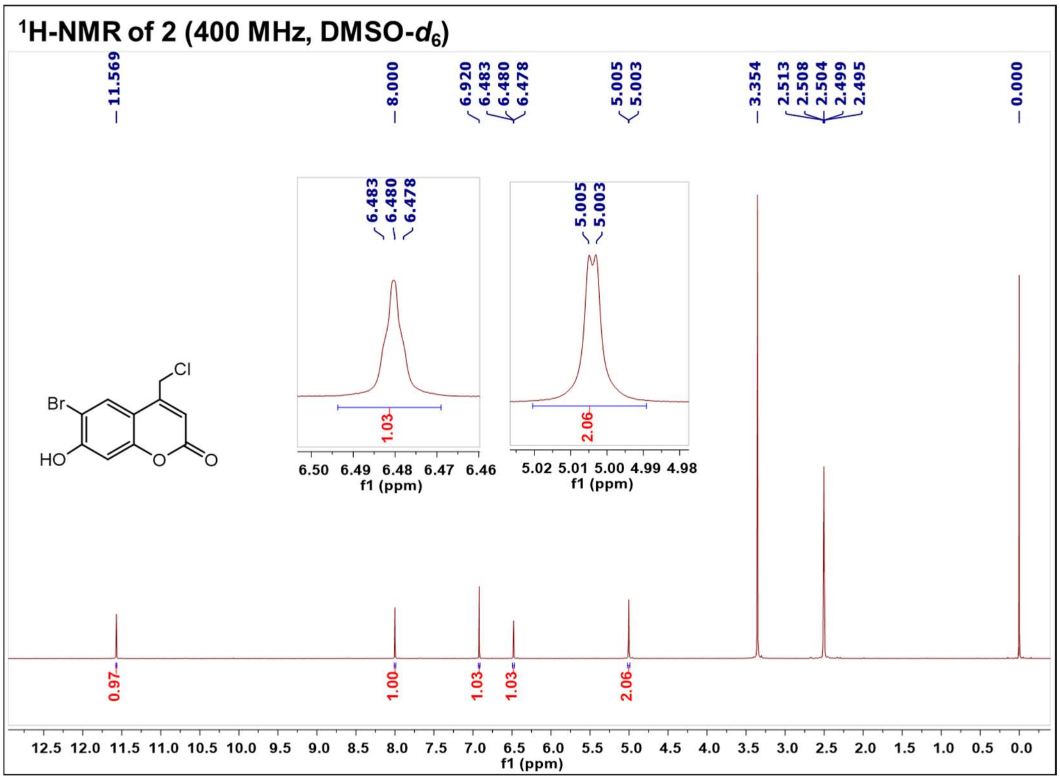

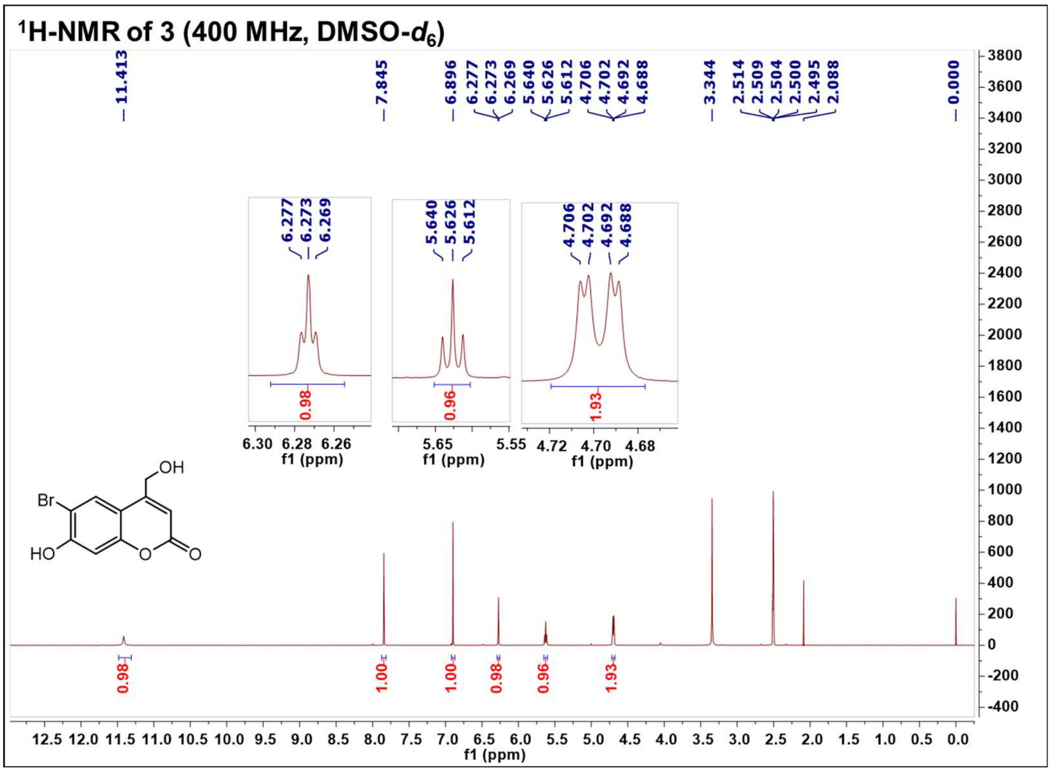

^{1}H-NMR of 4 (400 MHz, DMSO-d_6)

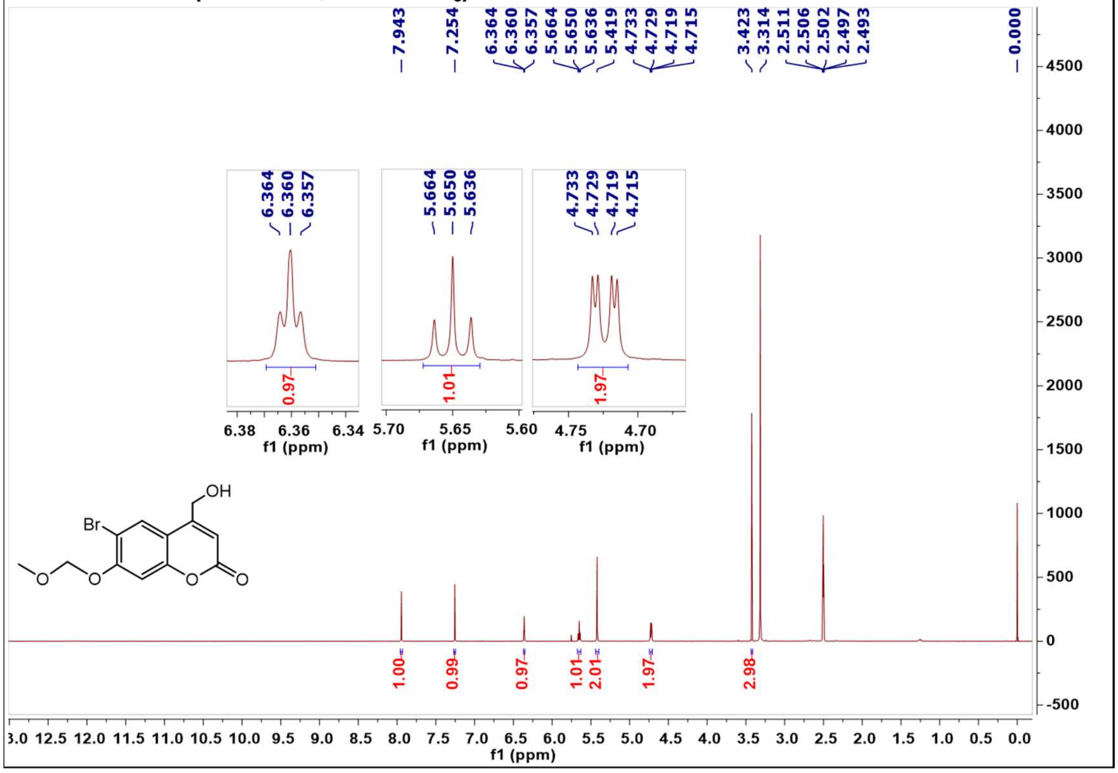

^{1}H-NMR of 5 (400 MHz, CDCl$_3$)

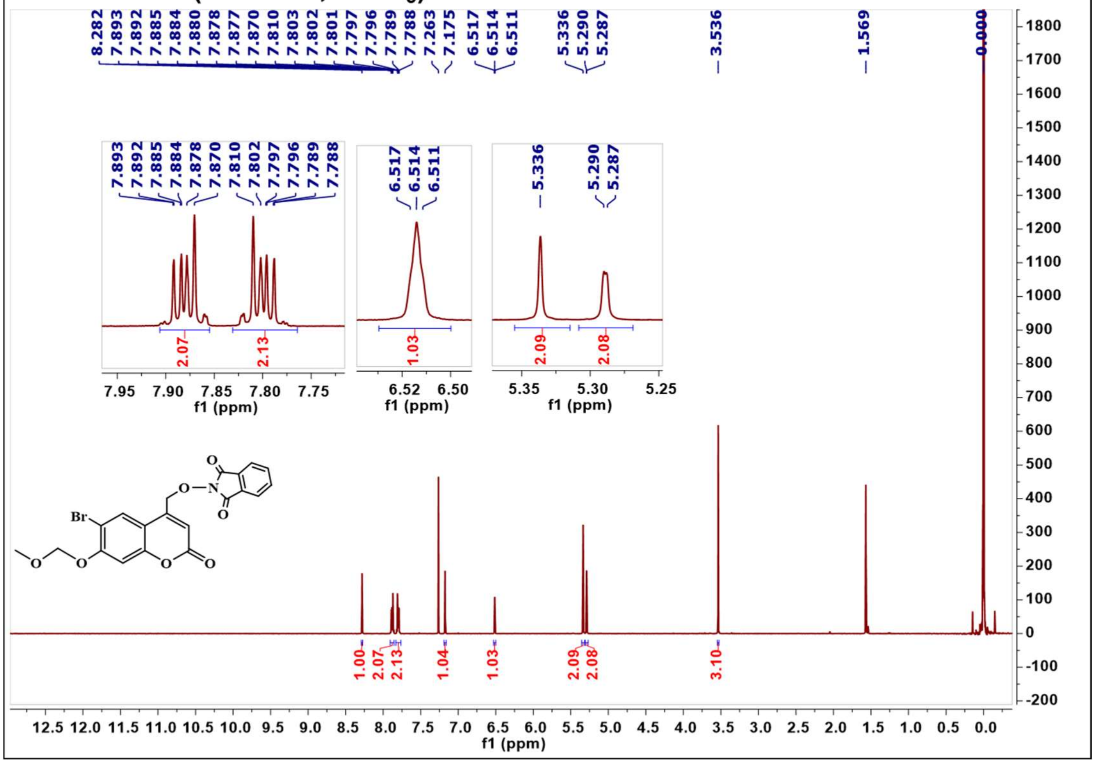

^{13}C-NMR of 5 (101 MHz, CDCl$_3$)

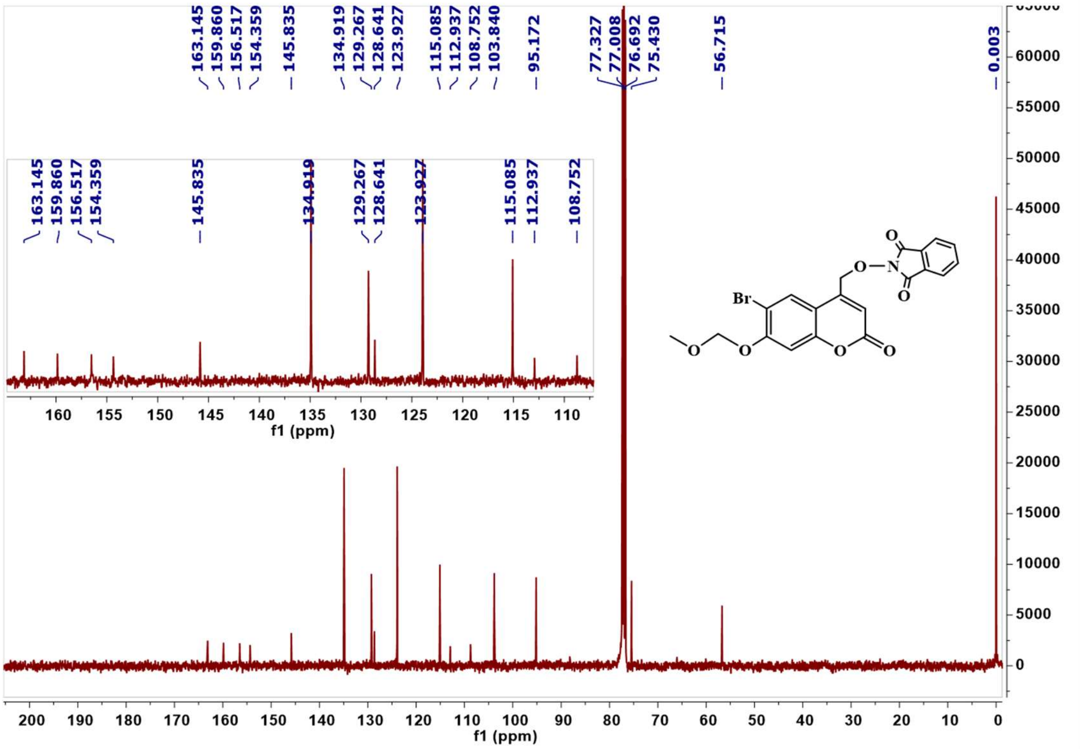

HRMS m/z (DART) data of 5

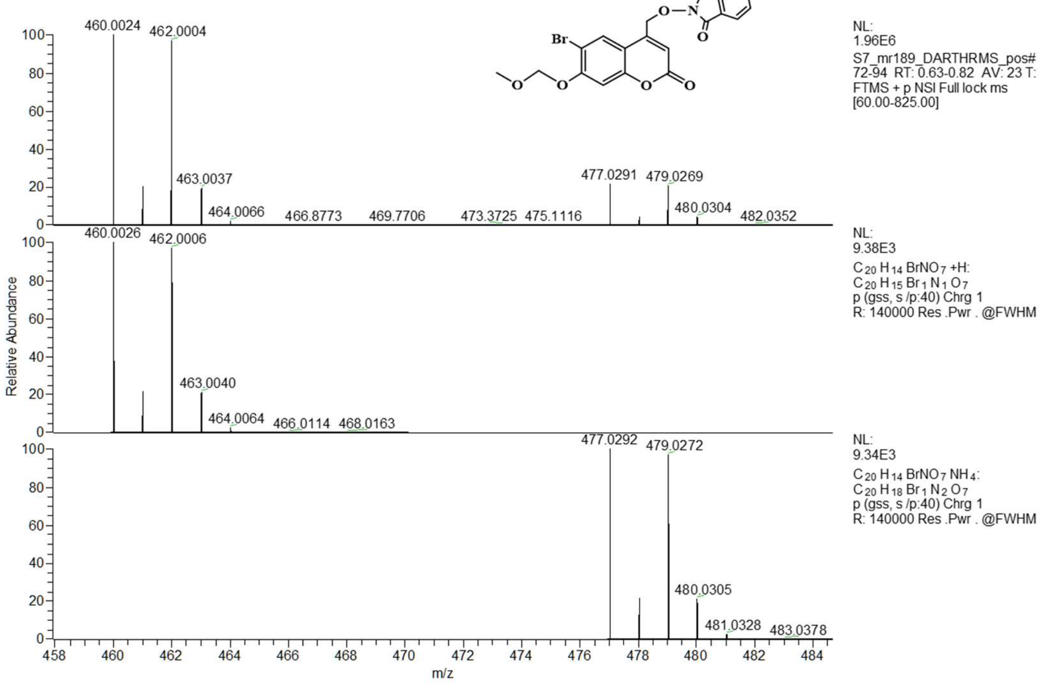

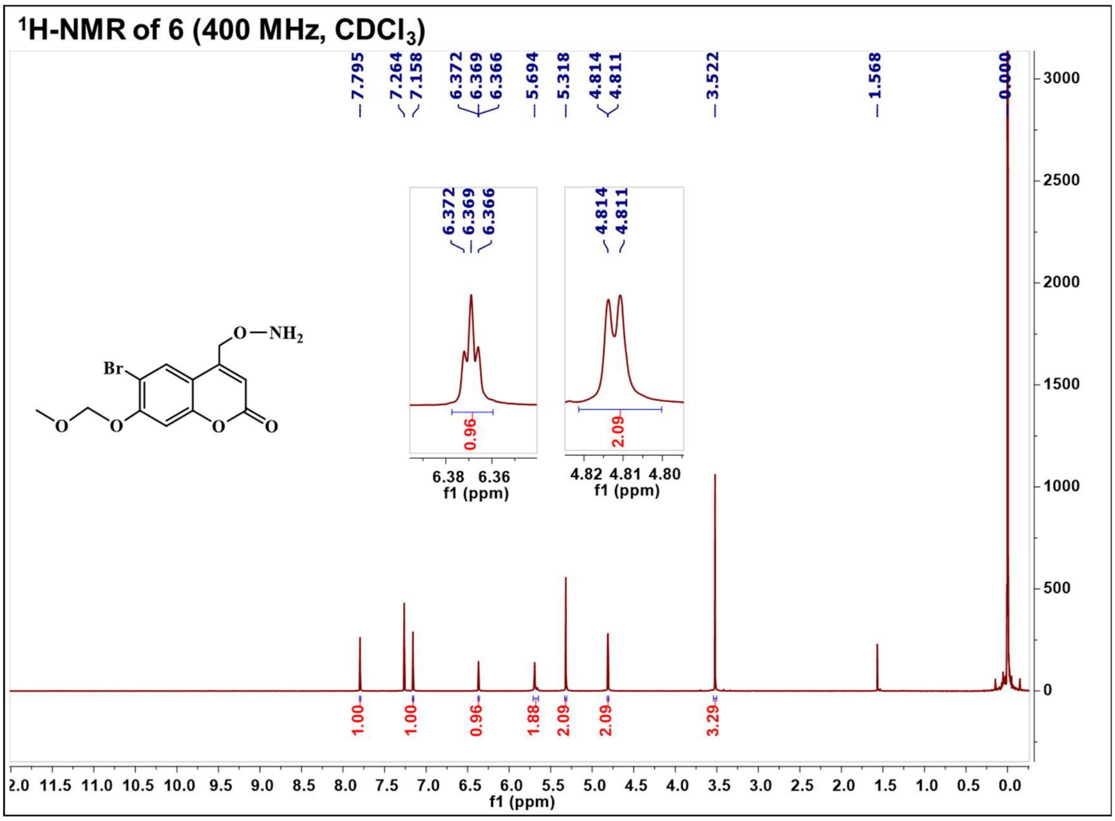

1H-NMR of 6 (400 MHz, CDCl3)
7.795
7.264
7.158
6.372
6.369
6.366
5.694
5.318
4.814
4.811
3.522
1.568
0.000
1.00
1.00
0.96
1.88
2.09
2.09
3.29
f1 (ppm)
O—NH2
Br
O
O
O
O

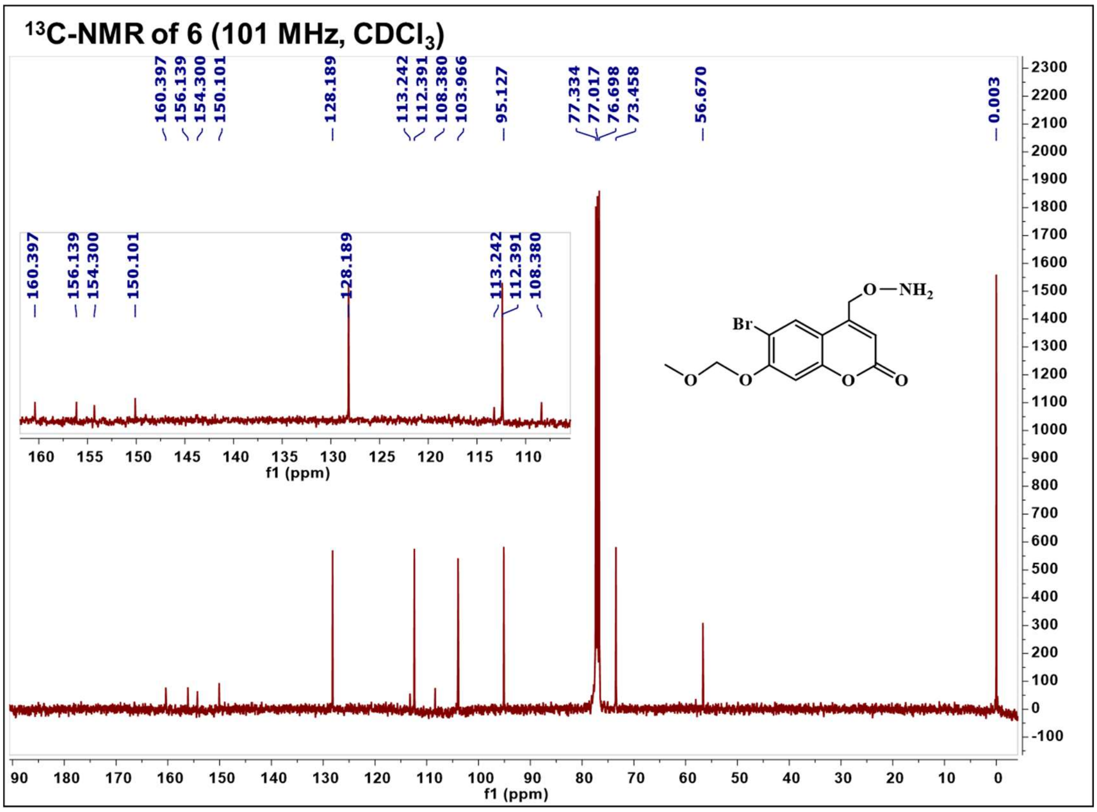

13C-NMR of 6 (101 MHz, CDCl3)
160.397
156.139
154.300
150.101
128.189
113.242
112.391
108.380
103.966
95.127
77.334
77.017
76.698
73.458
56.670
0.003
f1 (ppm)
O—NH2
Br
O
O
O
O

HRMS m/z (DART) data of 6

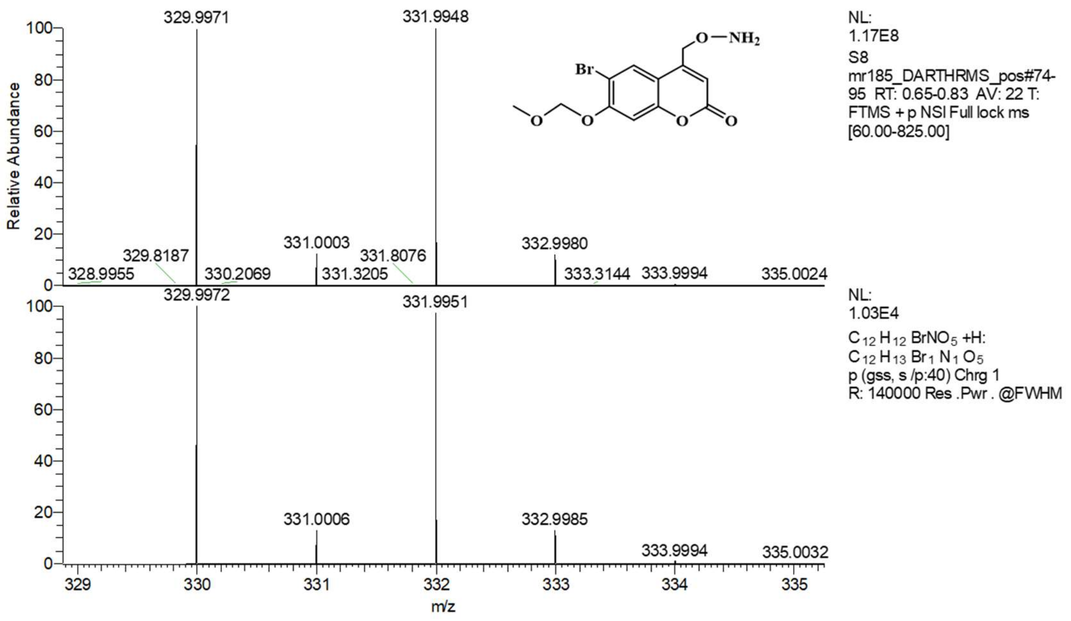

^{19}F-NMR of 7a (376 MHz, DMSO-d_6)

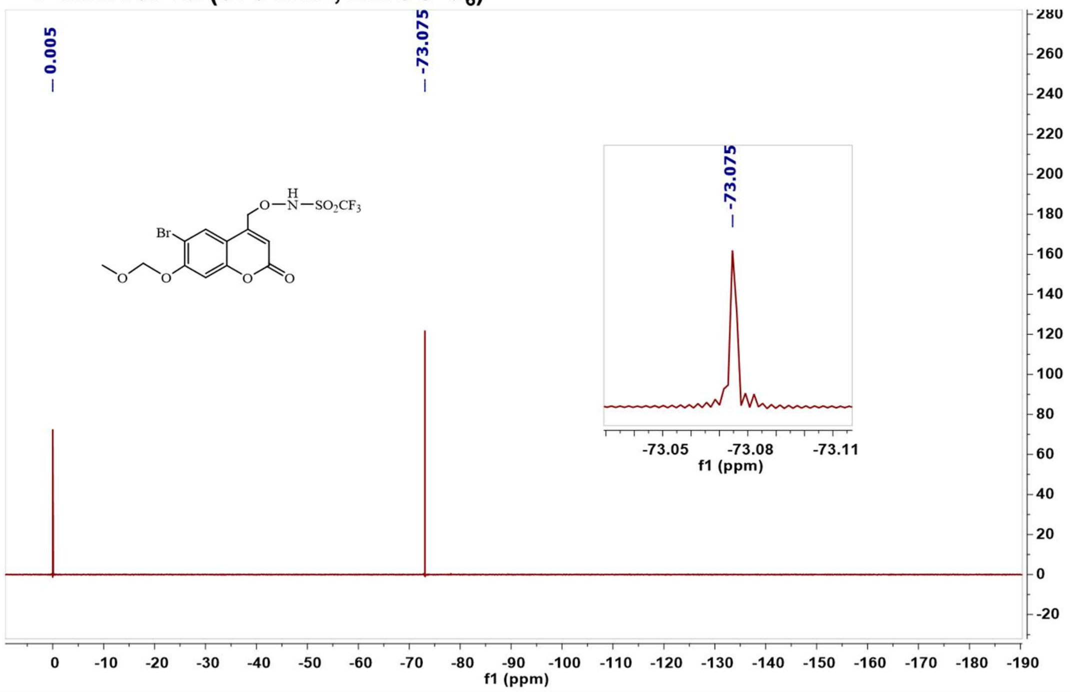

^{1}H-NMR of 7a (400 MHz, DMSO-d_6)

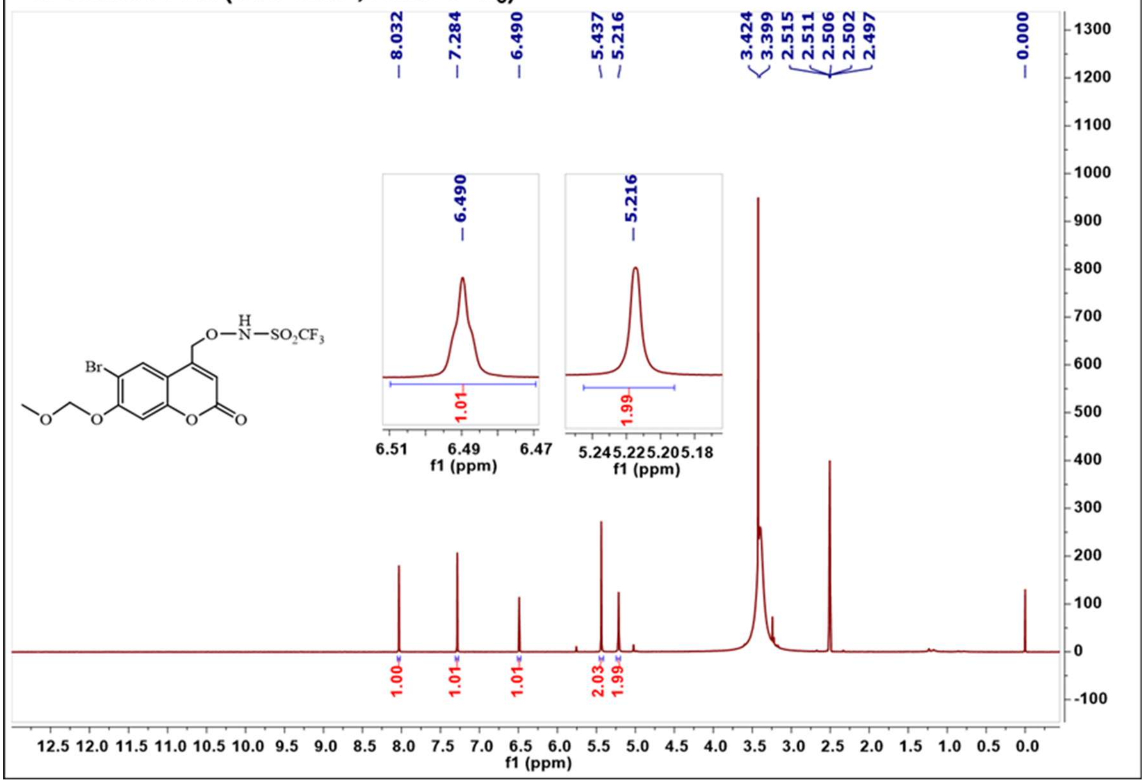

^{13}C-NMR of 7a (101 MHz, DMSO-d_6)

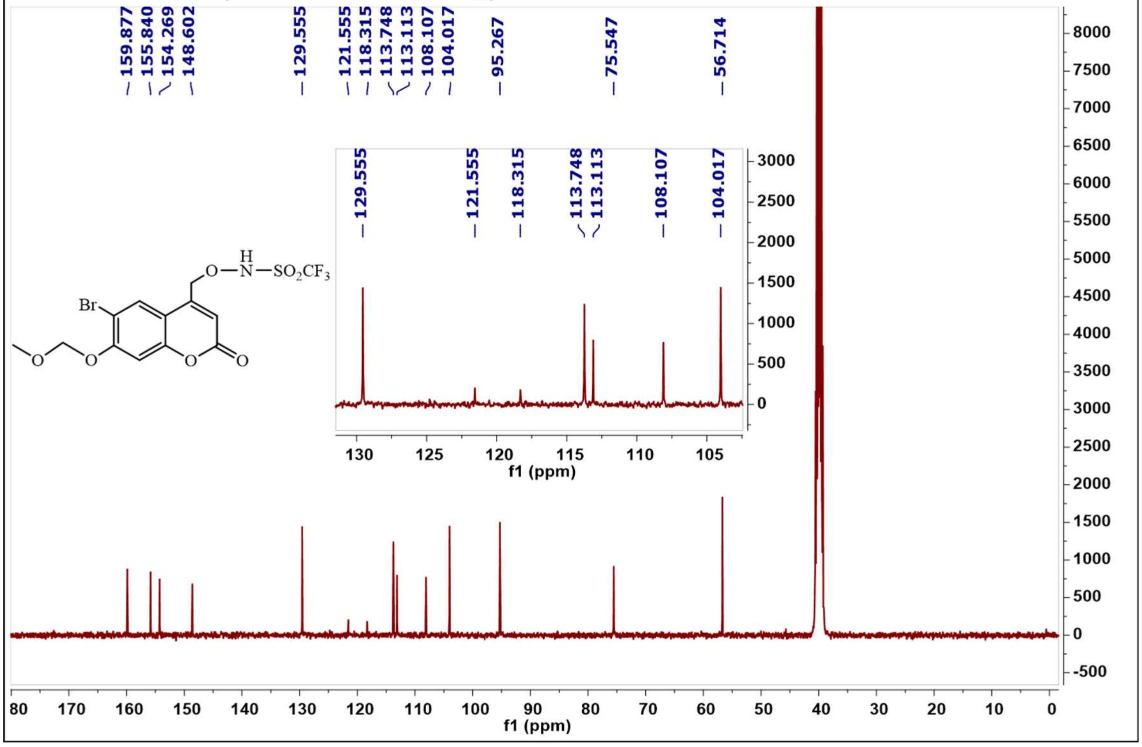

HRMS m/z (DART) data of 7a

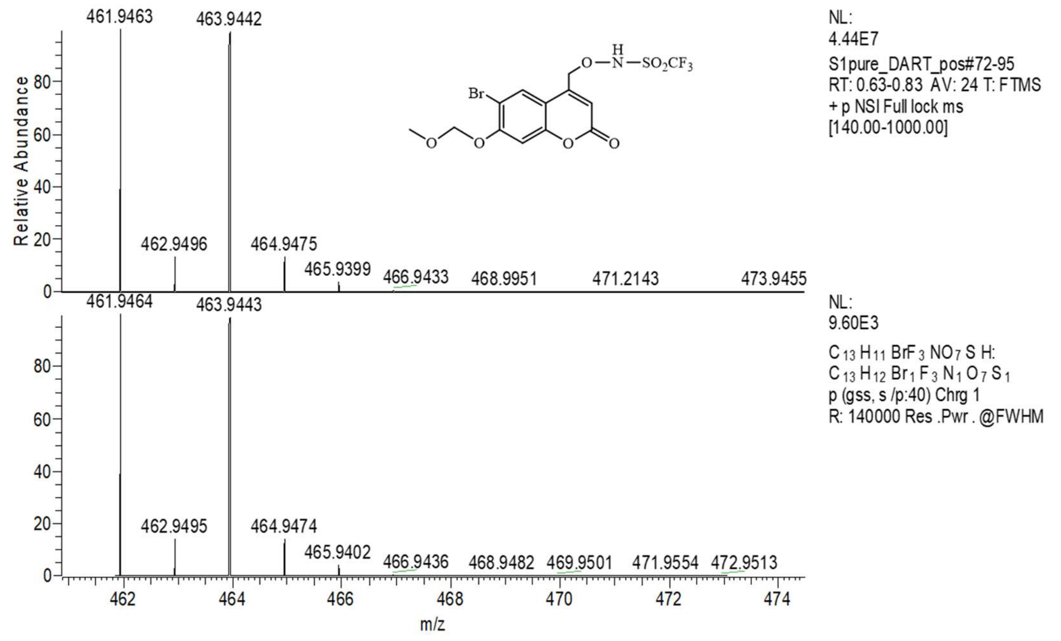

^{1}H-NMR of 7b (400 MHz, DMSO-d_6)

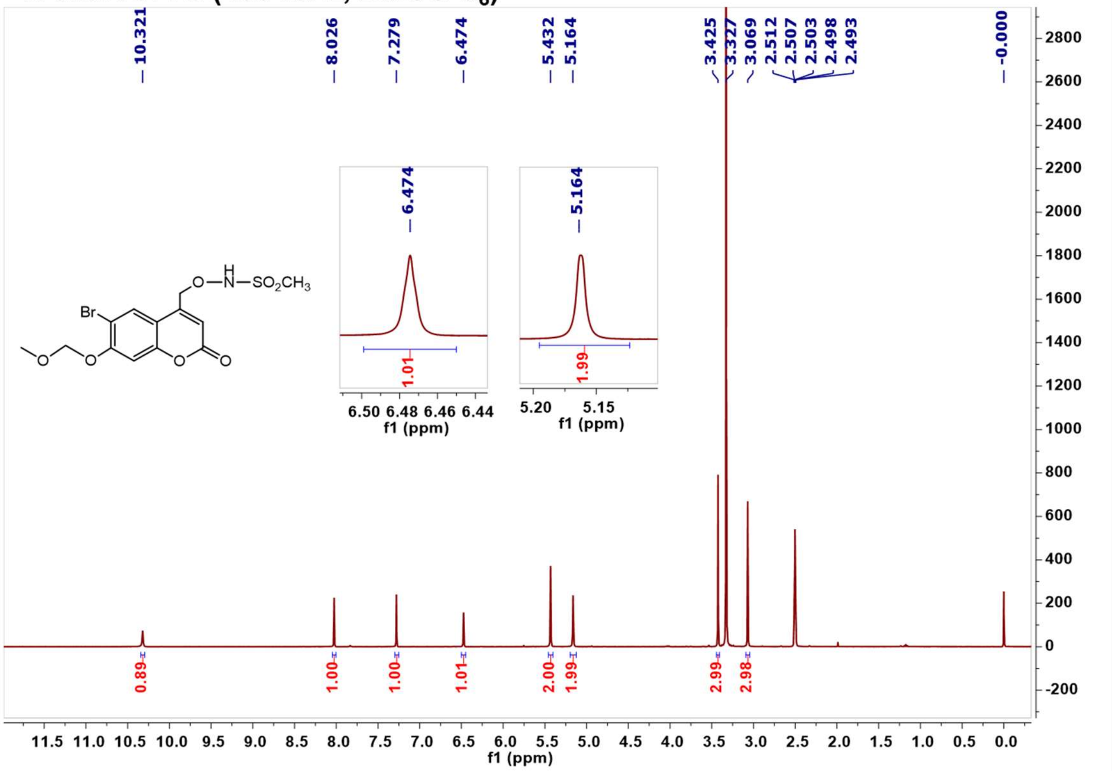

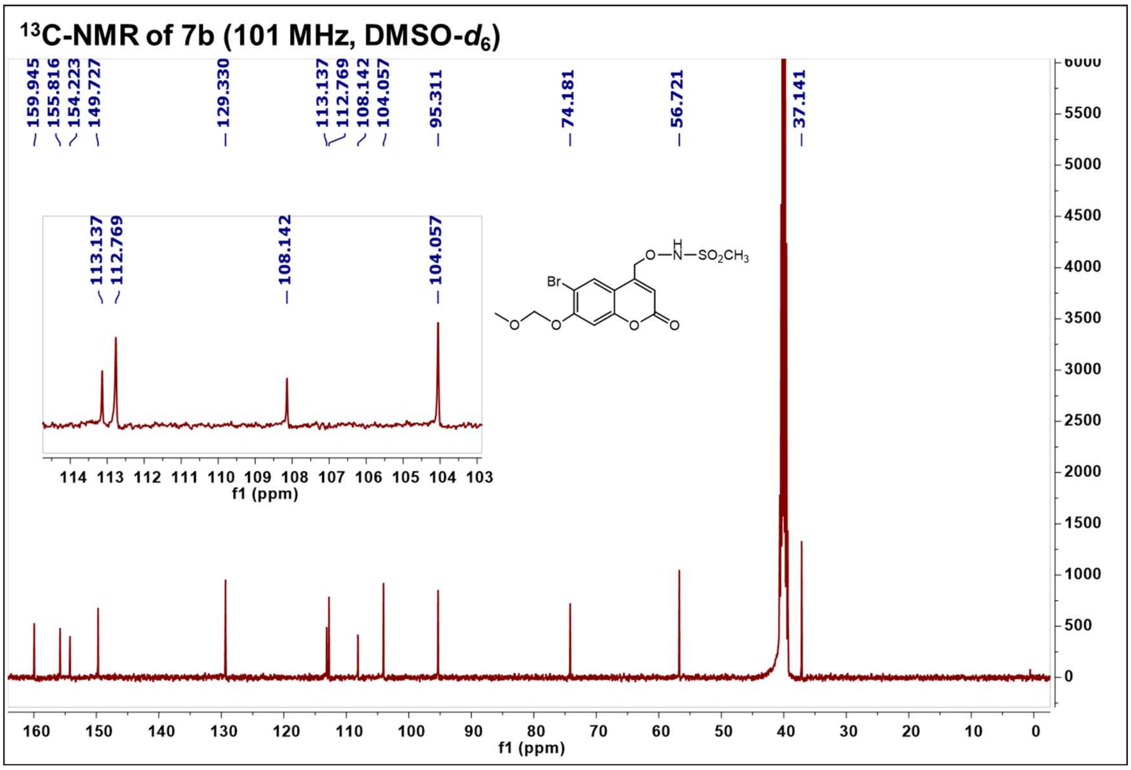
13C-NMR of 7b (101 MHz, DMSO-d6)
159.945
155.816
154.223
149.727
129.330
113.137
112.769
108.142
104.057
95.311
74.181
56.721
37.141
f1 (ppm)

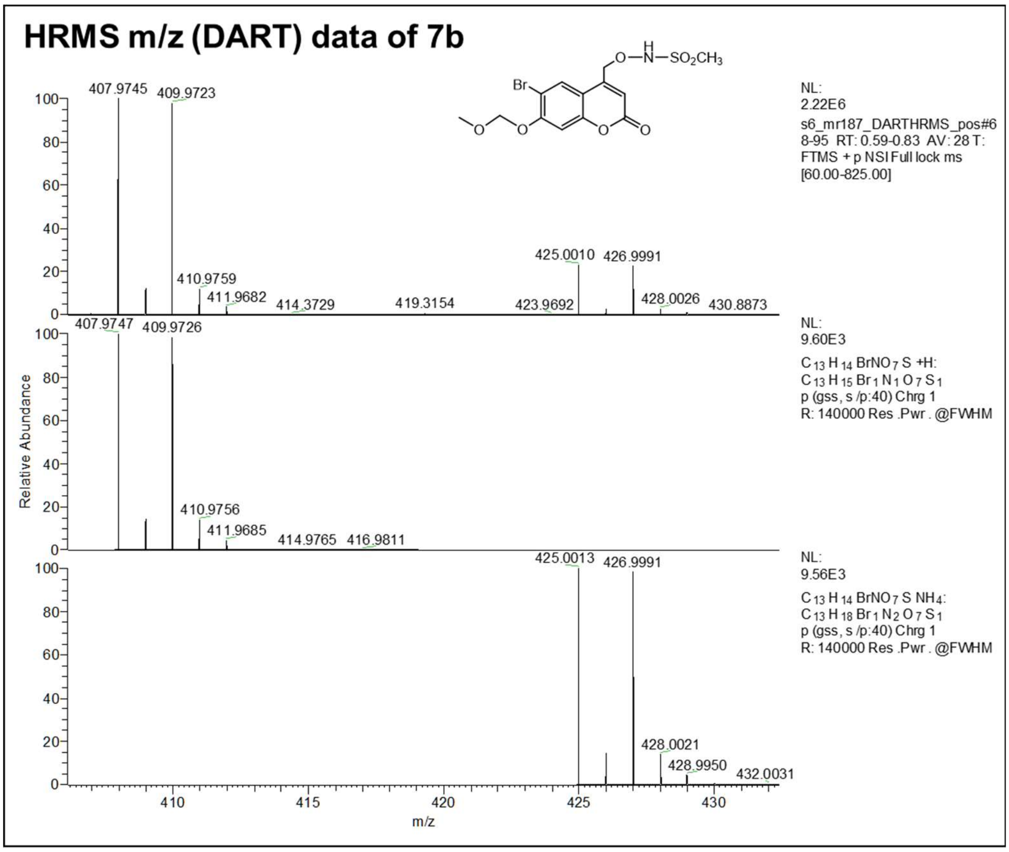
HRMS m/z (DART) data of 7b
407.9745
409.9723
410.9759
411.9682
425.0010
426.9991
NL: 2.22E6
s6_mr187_DARTHRMS_pos#68-95 RT: 0.59-0.83 AV: 28 T: FTMS + p NSI Full lock ms [60.00-825.00]
NL: 9.60E3
C 13 H 14 BrNO 7 S +H: C 13 H 15 Br 1 N 1 O 7 S 1
p (gss, s /p:40) Chrg 1
R: 140000 Res .Pwr . @FWHM
NL: 9.56E3
C 13 H 14 BrNO 7 S NH4: C 13 H 18 Br 1 N 2 O 7 S 1
p (gss, s /p:40) Chrg 1
R: 140000 Res .Pwr . @FWHM
Relative Abundance
m/z

^{1}H-NMR of 7c (400 MHz, DMSO-d_6)

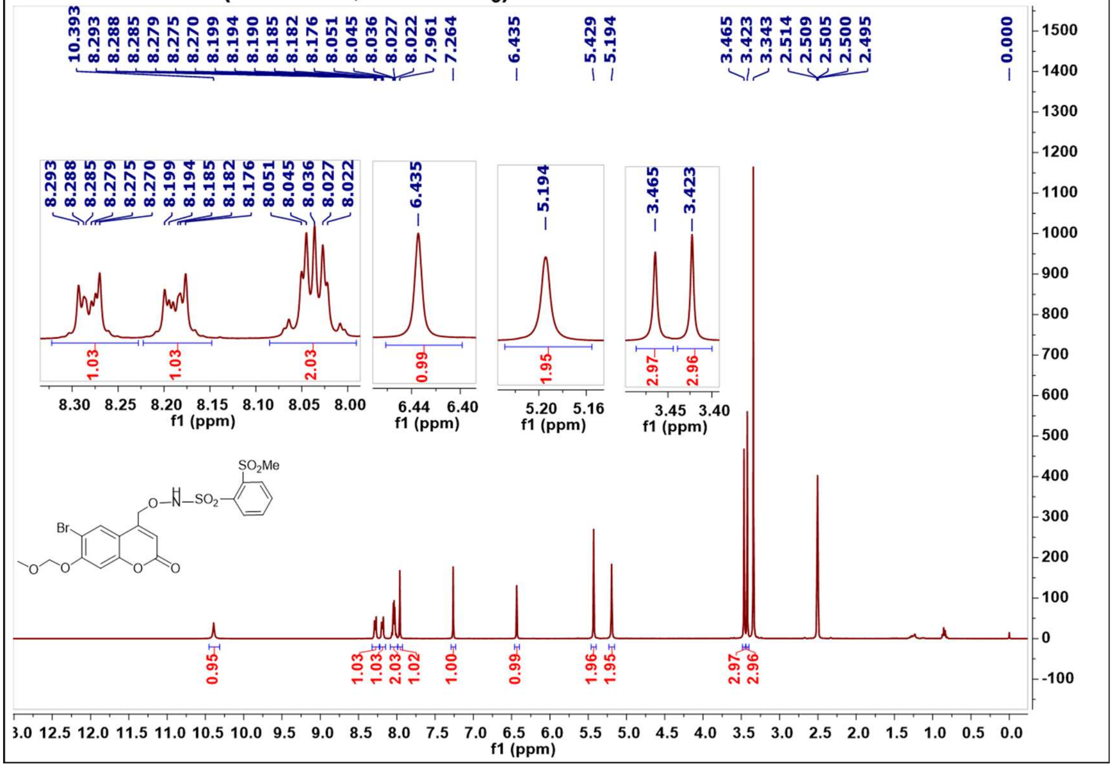

^{13}C-NMR of 7c (101 MHz, DMSO-d_6)

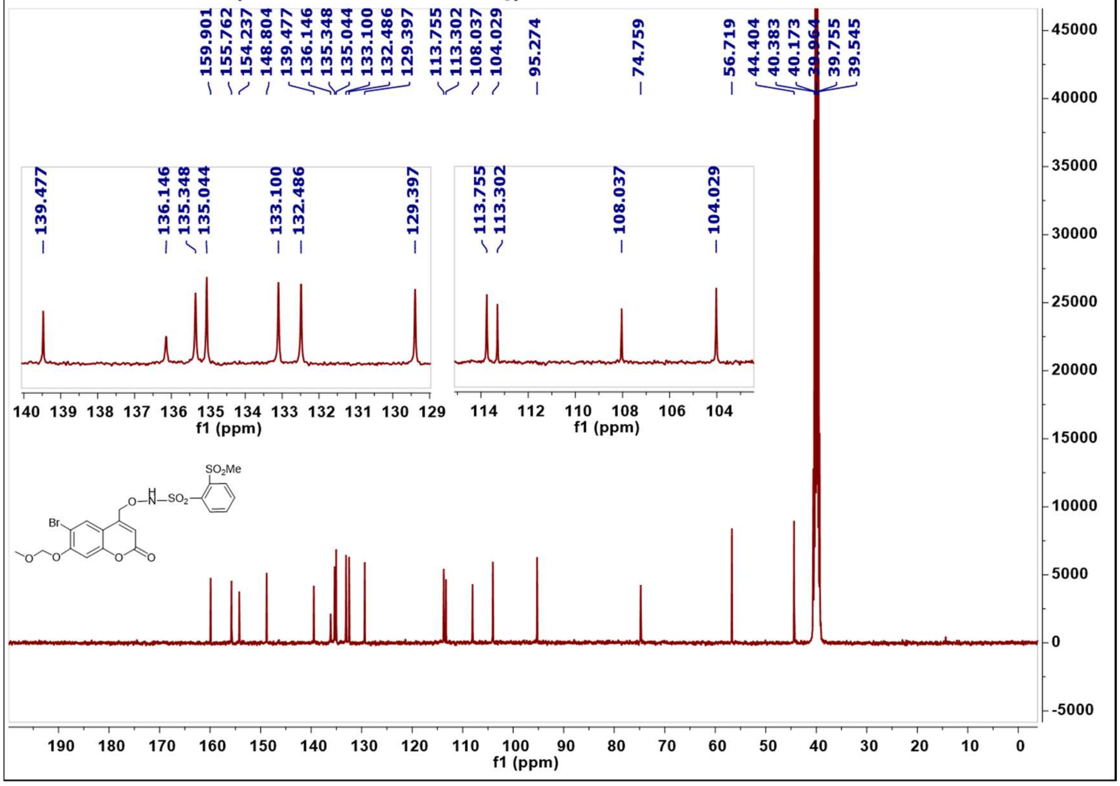

HRMS m/z (DART) data of 7c

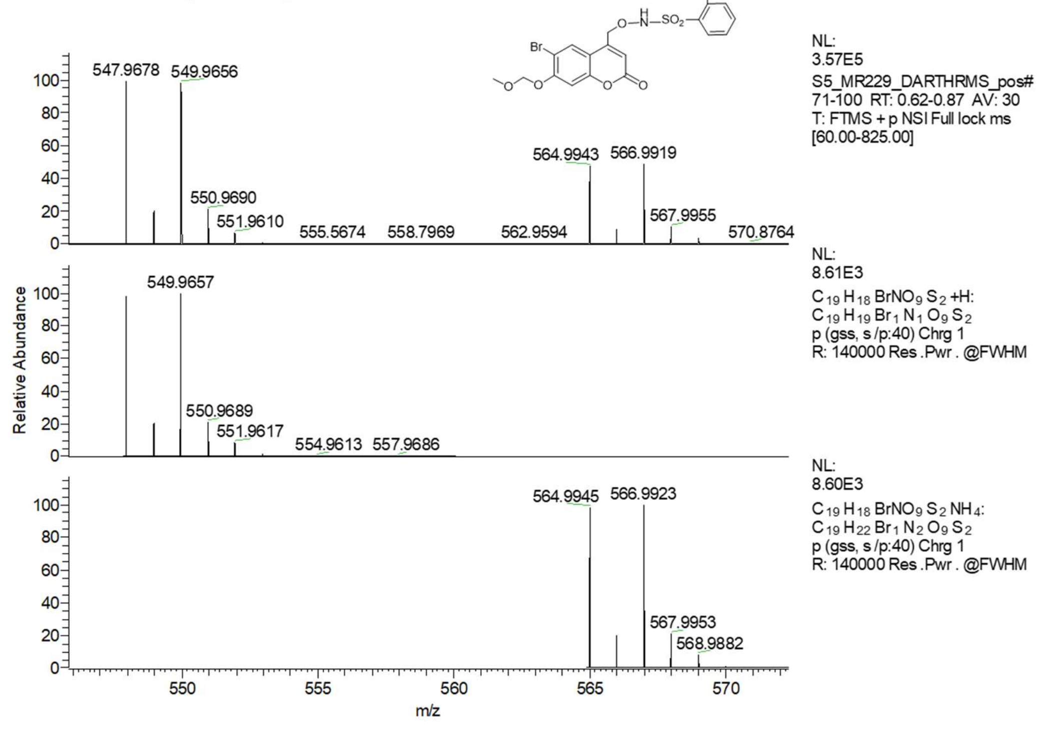

^{19}F-NMR of 8a (376 MHz, DMSO-d_6)

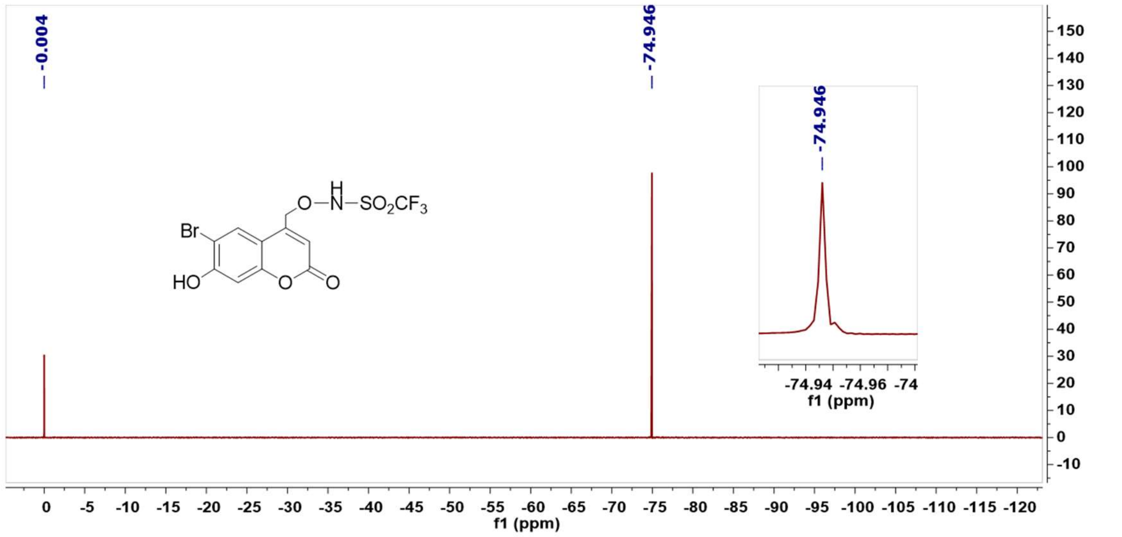

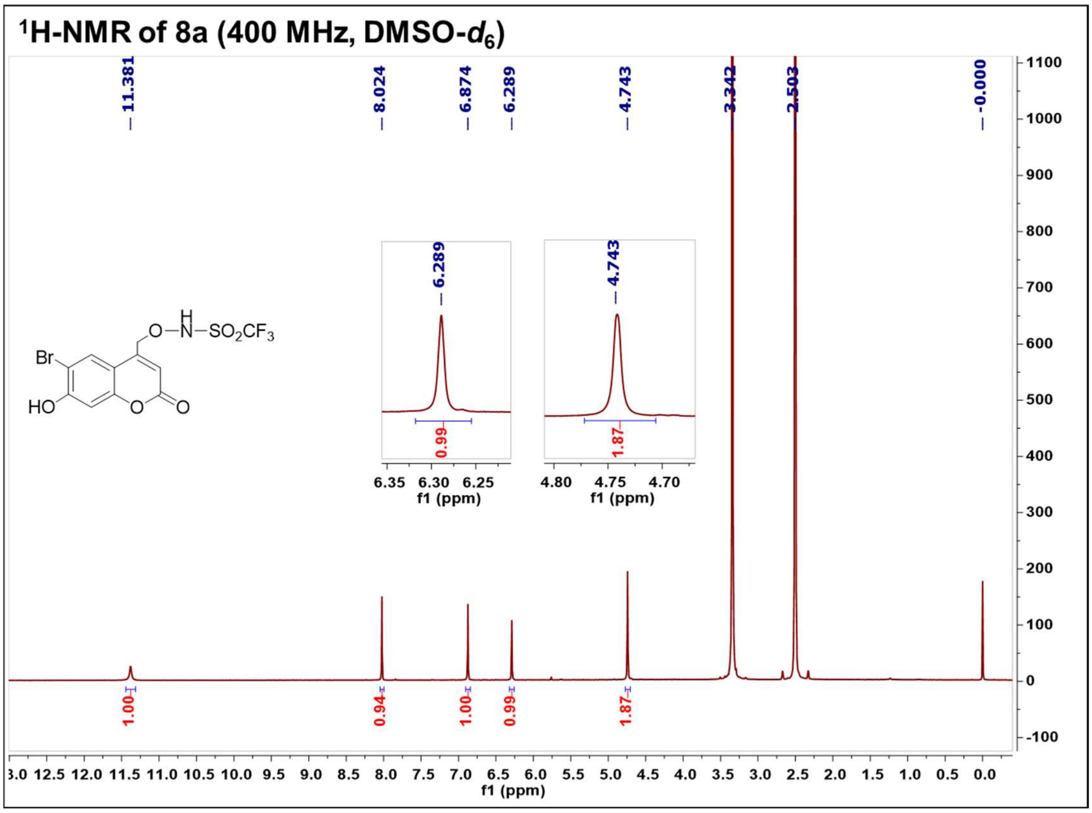

^{1}H-NMR of 8a (400 MHz, DMSO-d_6)
11.381
8.024
6.874
6.289
4.743
3.342
2.503
-0.000
O–NH–SO2CF3
Br
HO
O
O
1.00
0.94
1.00
0.99
1.87
f1 (ppm)

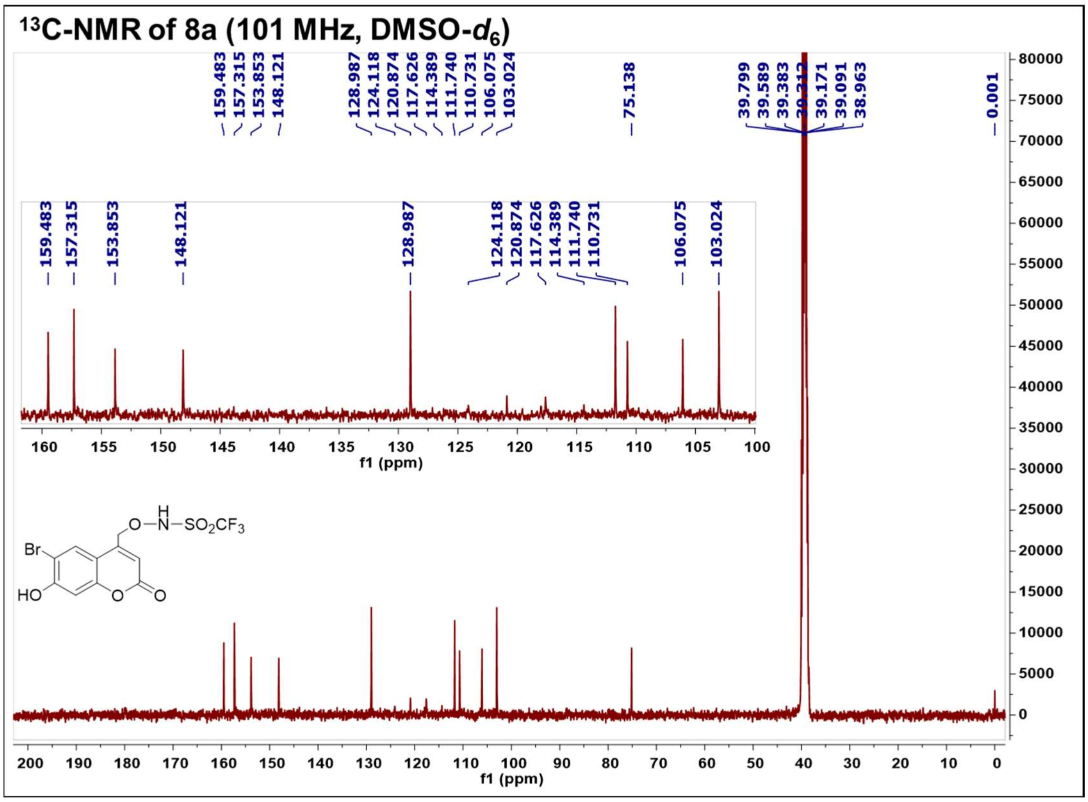

^{13}C-NMR of 8a (101 MHz, DMSO-d_6)
159.483
157.315
153.853
148.121
128.987
124.118
120.874
117.626
114.389
111.740
110.731
106.075
103.024
75.138
39.799
39.589
39.383
39.171
39.091
38.963
0.001
O–NH–SO2CF3
Br
HO
O
O
f1 (ppm)

HRMS m/z (DART) data of 8a

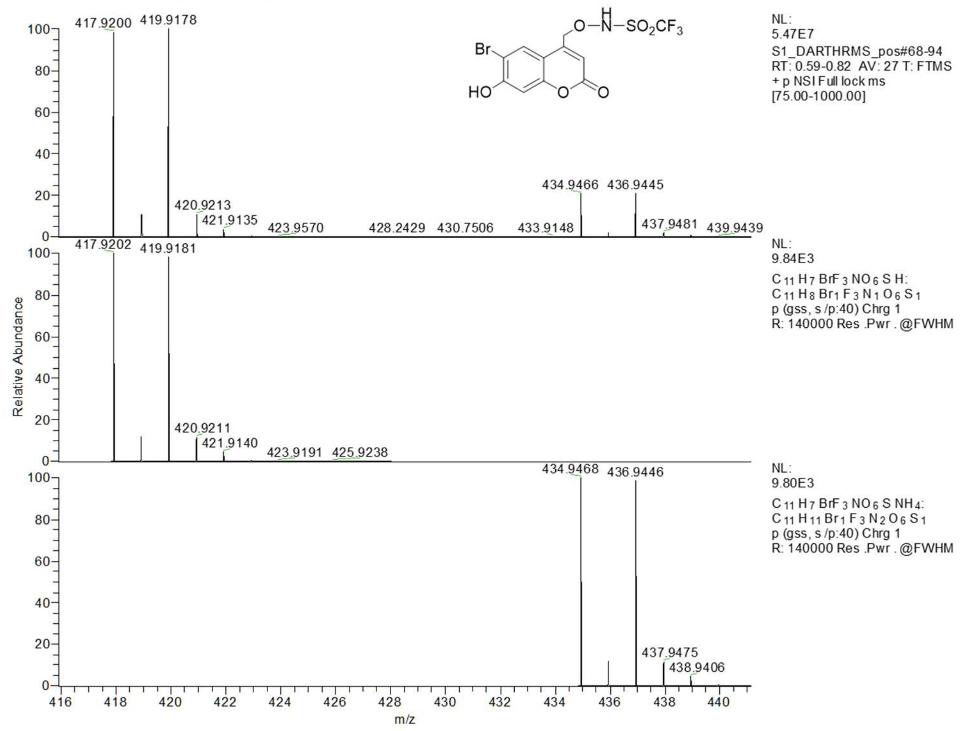

^{1}H-NMR of 8b (400 MHz, DMSO-d_6)

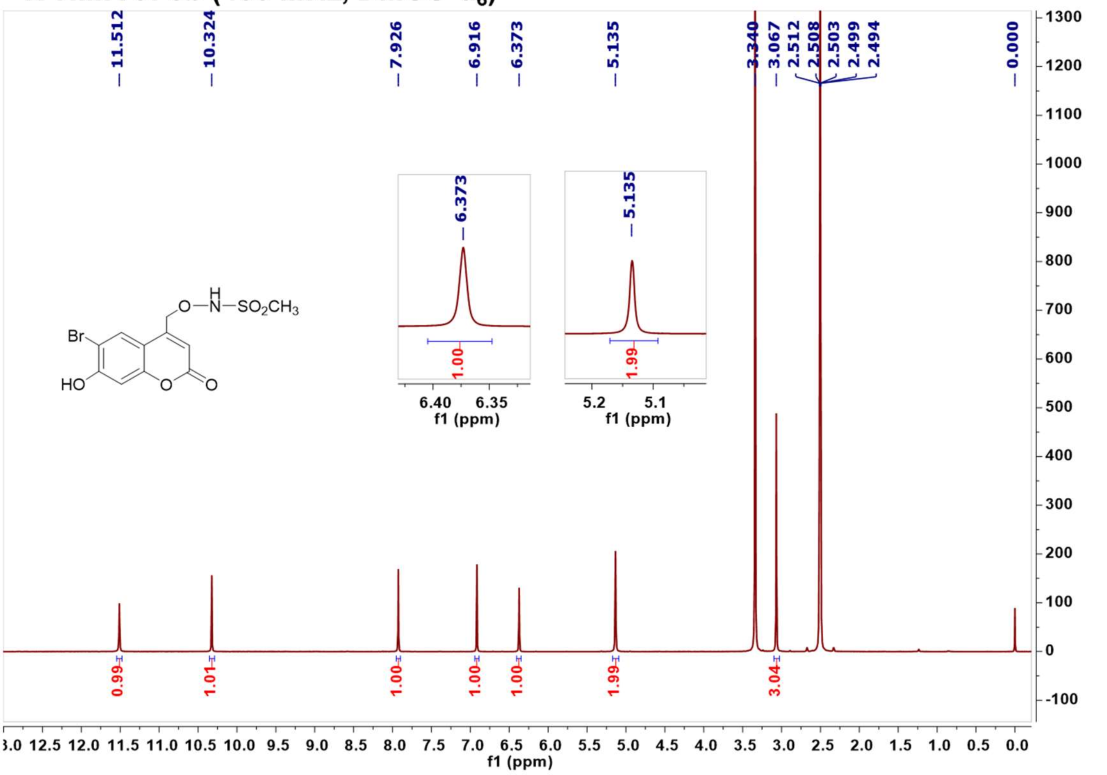

^{13}C-NMR of 8b (101 MHz, DMSO-d_6)

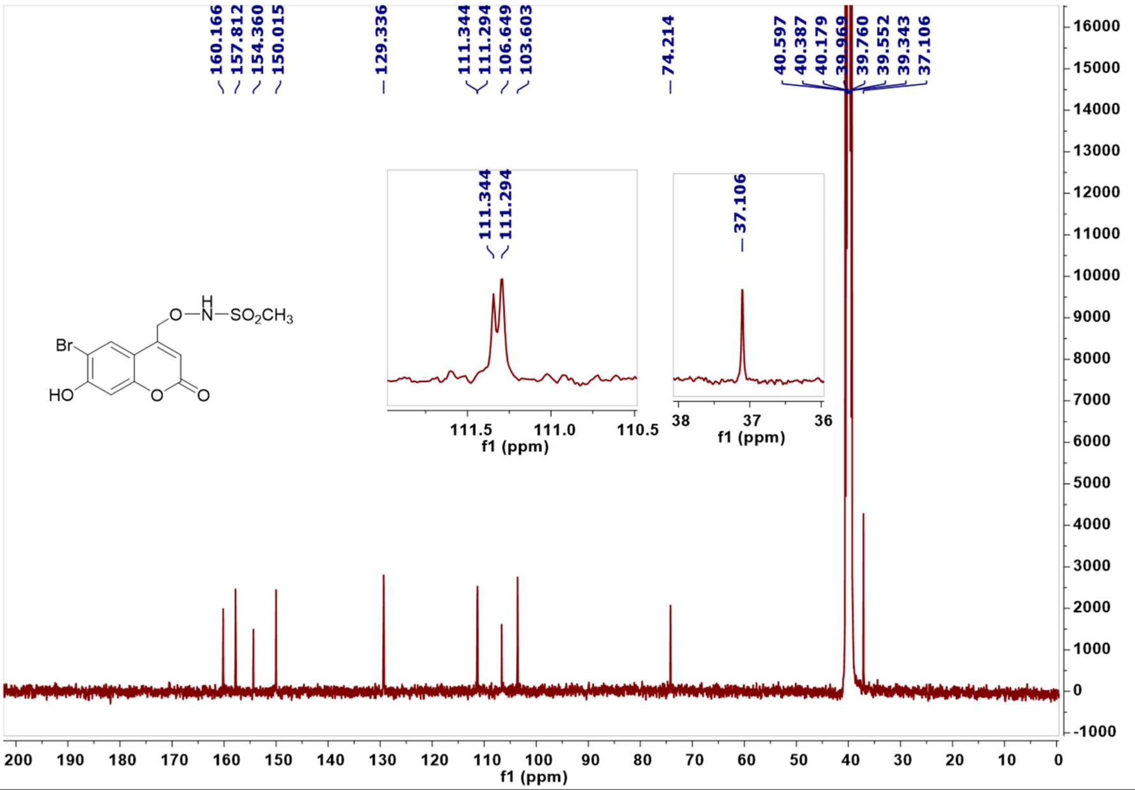

HRMS m/z (DART) data of 8b

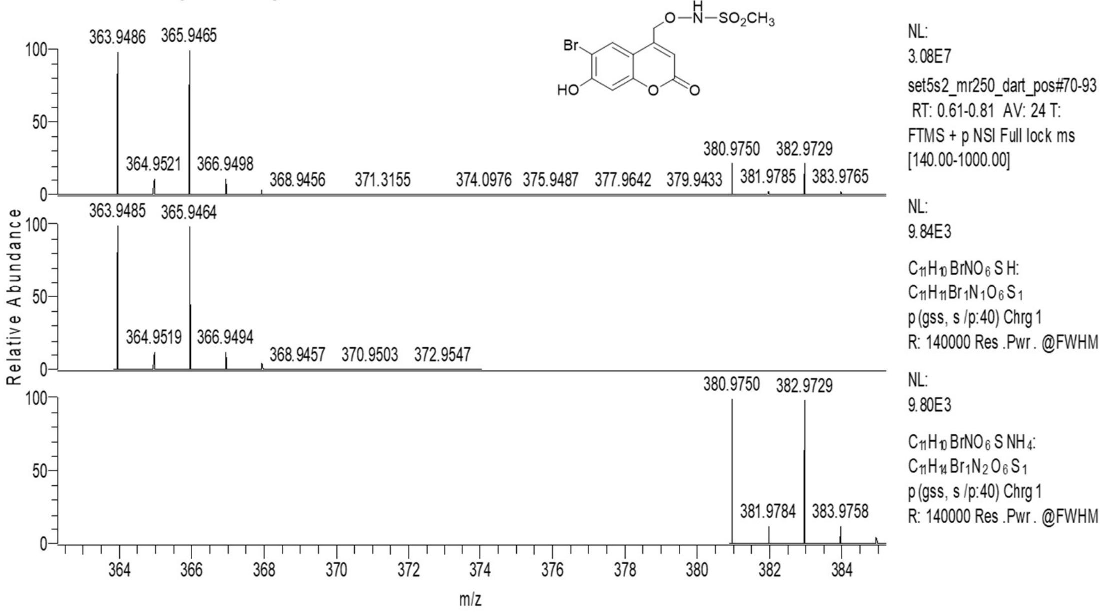

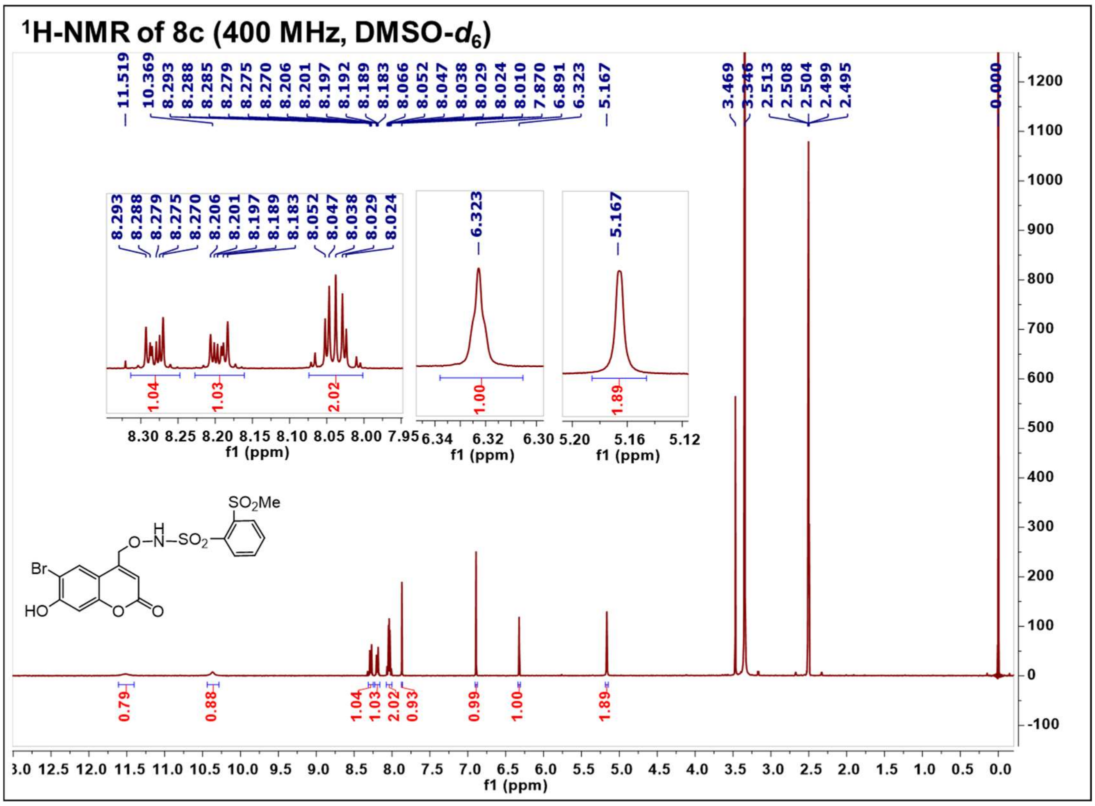

^{1}H-NMR of 8c (400 MHz, DMSO-d_6)
11.519
10.369
8.293
8.288
8.285
8.279
8.275
8.270
8.206
8.201
8.197
8.192
8.189
8.183
8.066
8.052
8.047
8.038
8.029
8.024
8.010
7.870
6.891
6.323
5.167
3.469
3.346
2.513
2.508
2.504
2.499
2.495
0.000
SO2Me
H
O–N–SO2
Br
HO
O
O
f1 (ppm)

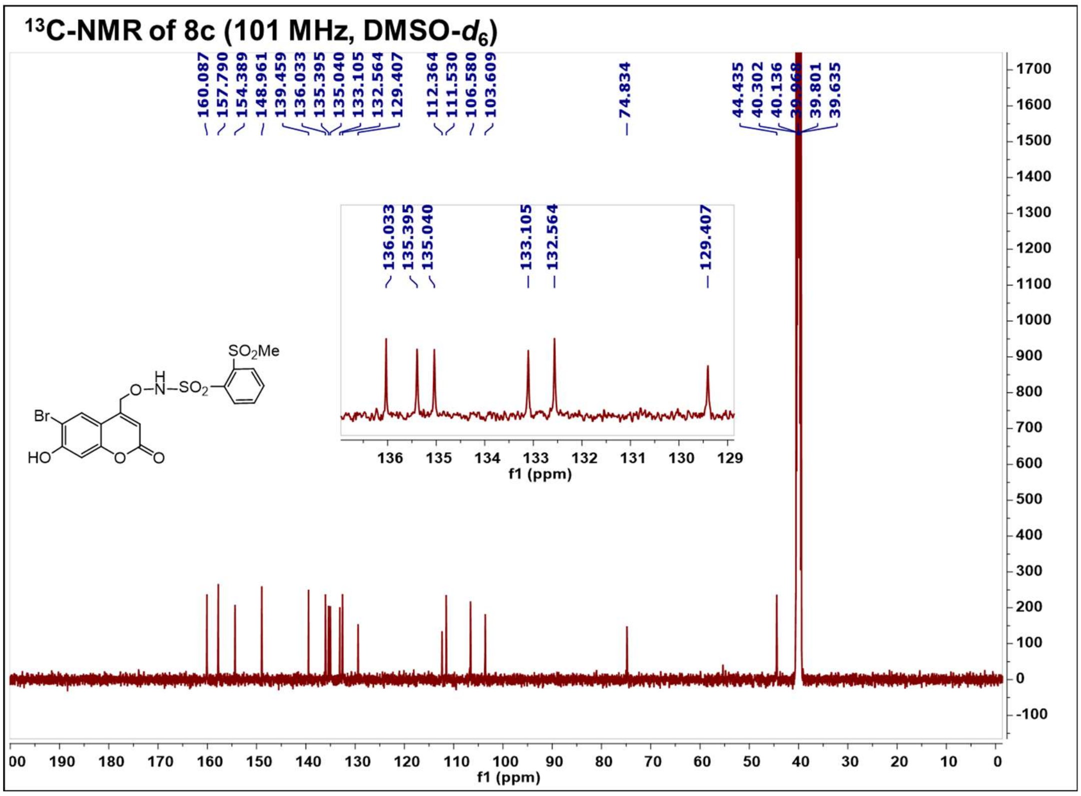

^{13}C-NMR of 8c (101 MHz, DMSO-d_6)
160.087
157.790
154.389
148.961
139.459
136.033
135.395
135.040
133.105
132.564
129.407
112.364
111.530
106.580
103.609
74.834
44.435
40.302
40.136
39.801
39.635
SO2Me
H
O–N–SO2
Br
HO
O
O
f1 (ppm)

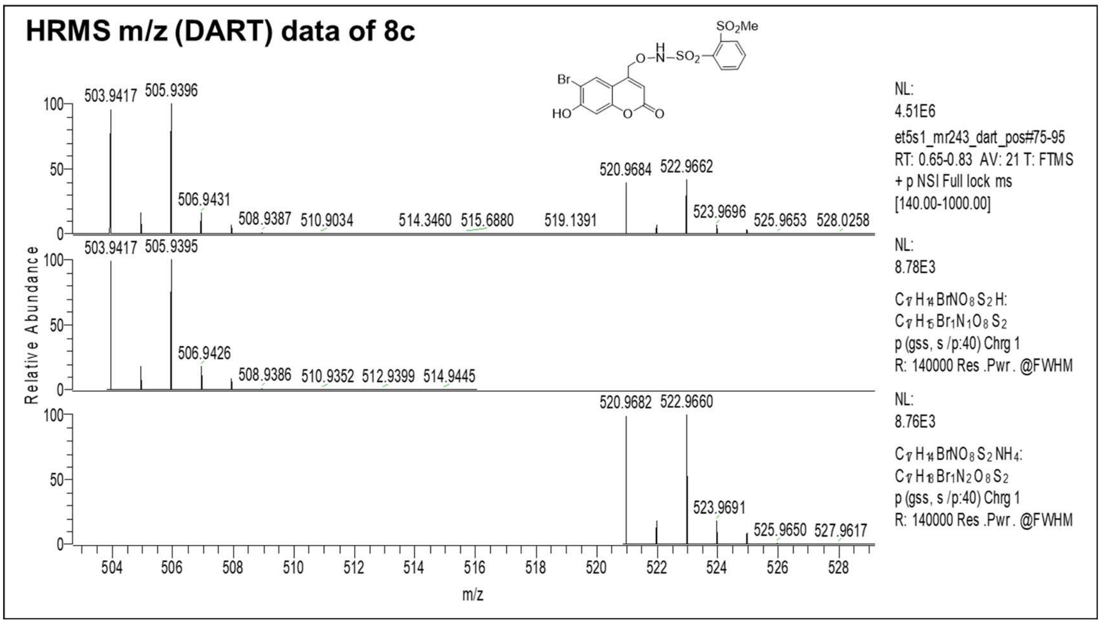
HRMS m/z (DART) data of 8c
SO2Me
O–N(H)–SO2
Br
HO
O O
503.9417 505.9396
506.9431
508.9387 510.9034 514.3460 515.6880 519.1391
520.9684 522.9662
523.9696 525.9653 528.0258
NL: 4.51E6
et5s1_mr243_dart_pos#75-95
RT: 0.65-0.83 AV: 21 T: FTMS + p NSI Full lock ms [140.00-1000.00]
503.9417 505.9395
506.9426
508.9386 510.9352 512.9399 514.9445
NL: 8.78E3
C17H14BrNO8S2H: C17H15Br1N1O8S2
p (gss, s /p:40) Chrg 1
R: 140000 Res .Pwr . @FWHM
520.9682 522.9660
523.9691
525.9650 527.9617
NL: 8.76E3
C17H14BrNO8S2NH4: C17H18Br1N2O8S2
p (gss, s /p:40) Chrg 1
R: 140000 Res .Pwr . @FWHM
Relative Abundance
m/z

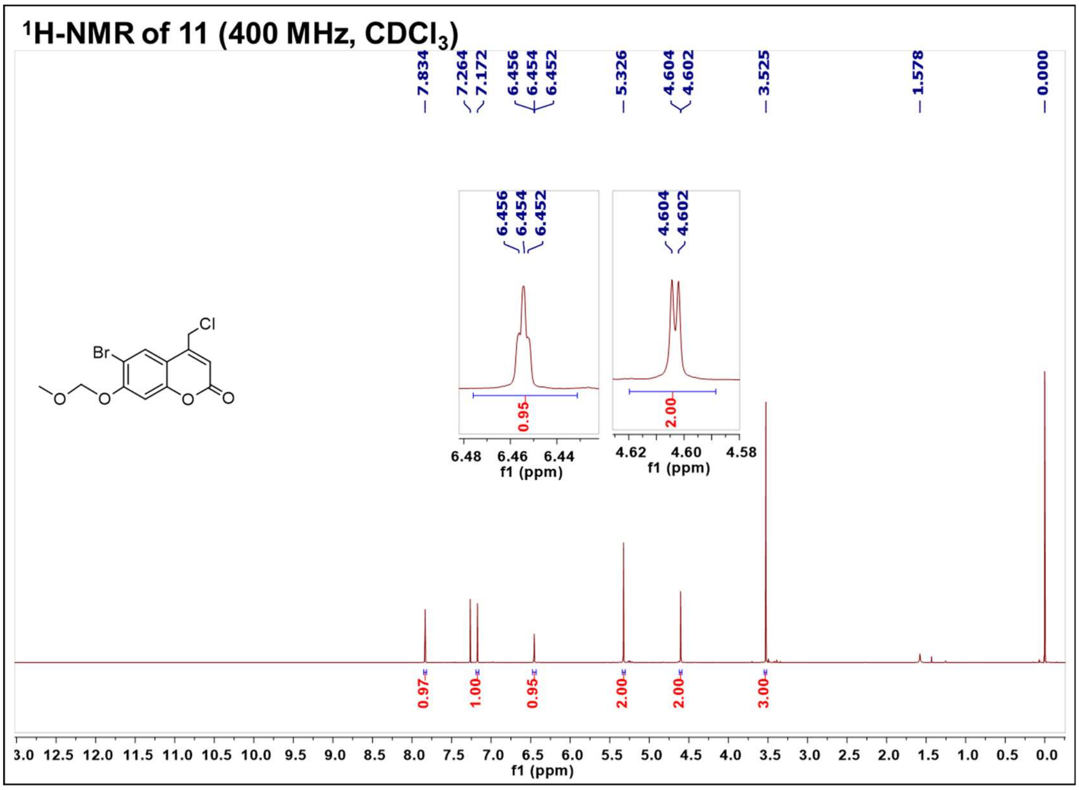
1H-NMR of 11 (400 MHz, CDCl3)
7.834 7.264 7.172 6.456 6.454 6.452 5.326 4.604 4.602 3.525 1.578 0.000
Cl
Br
O O O O
0.95
2.00
f1 (ppm)
0.97 1.00 0.95 2.00 2.00 3.00
f1 (ppm)

^{1}H-NMR of 5 (400 MHz, $CDCl_3$)

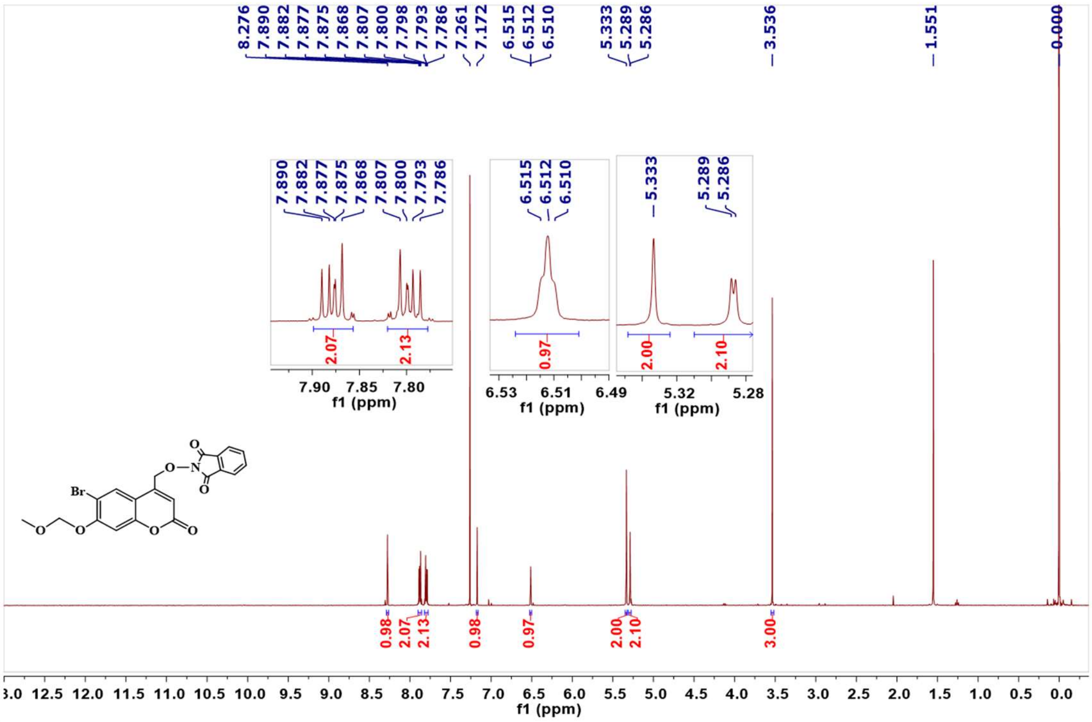

^{1}H-NMR of 12 crude (400 MHz, DMSO-d_6)

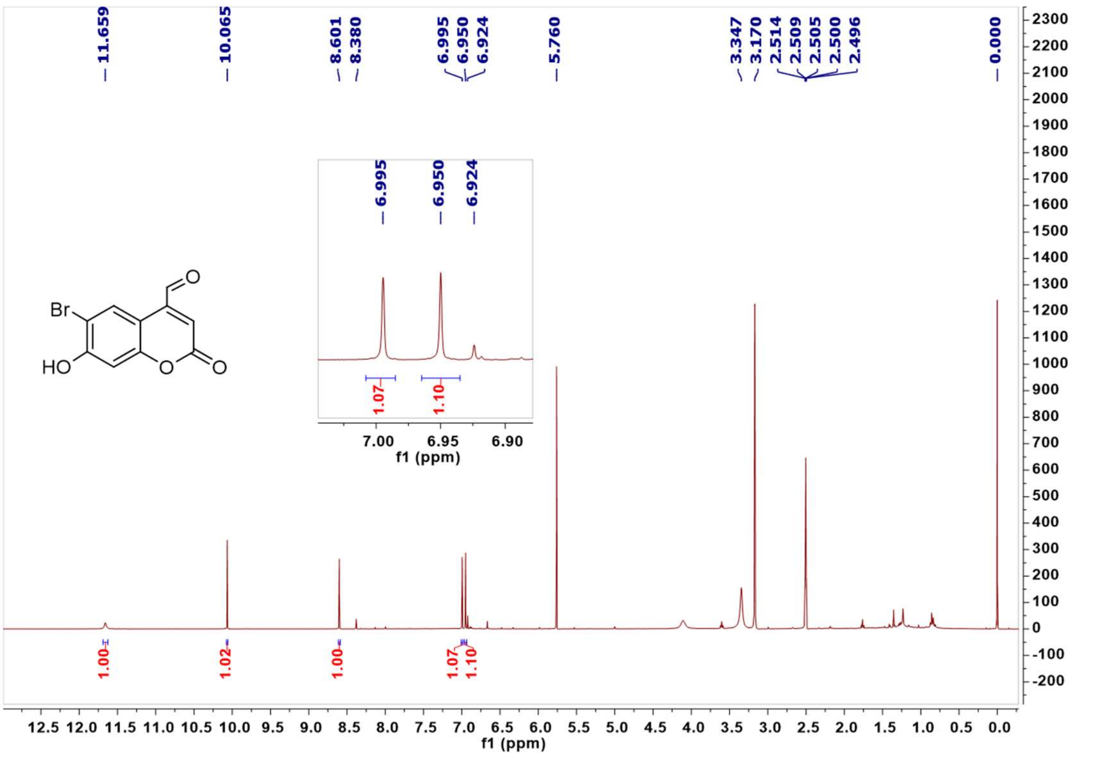

^{13}C-NMR of 12 crude (101 MHz, DMSO-d_6)

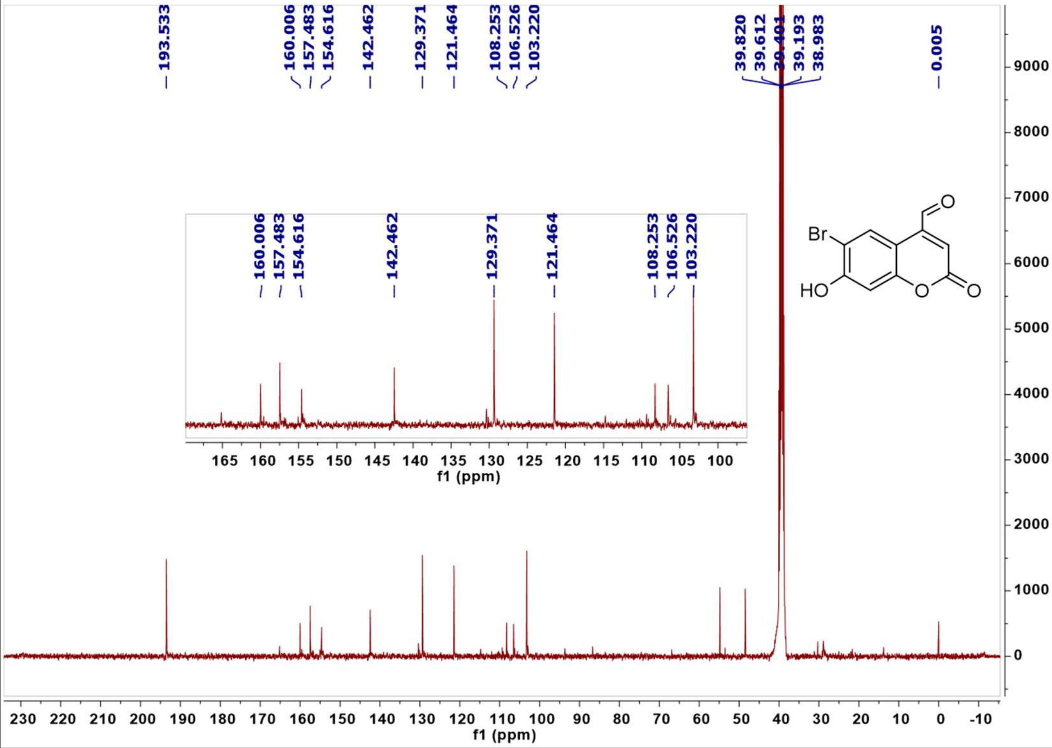

^{1}H-NMR of 13 (400 MHz, DMSO-d_6)

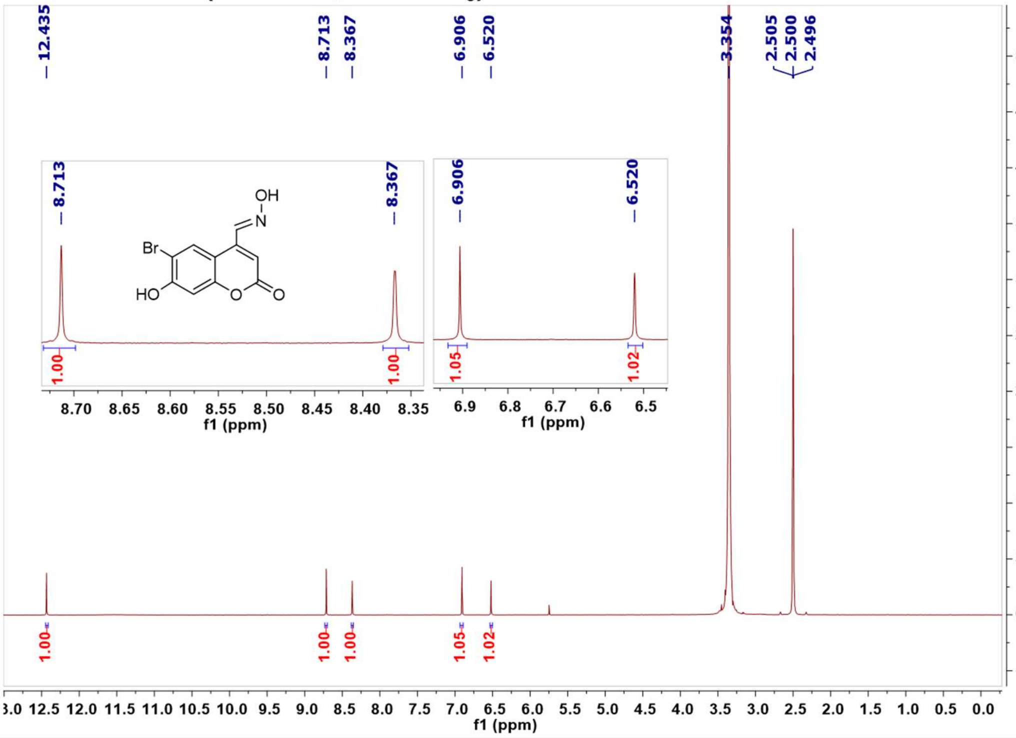

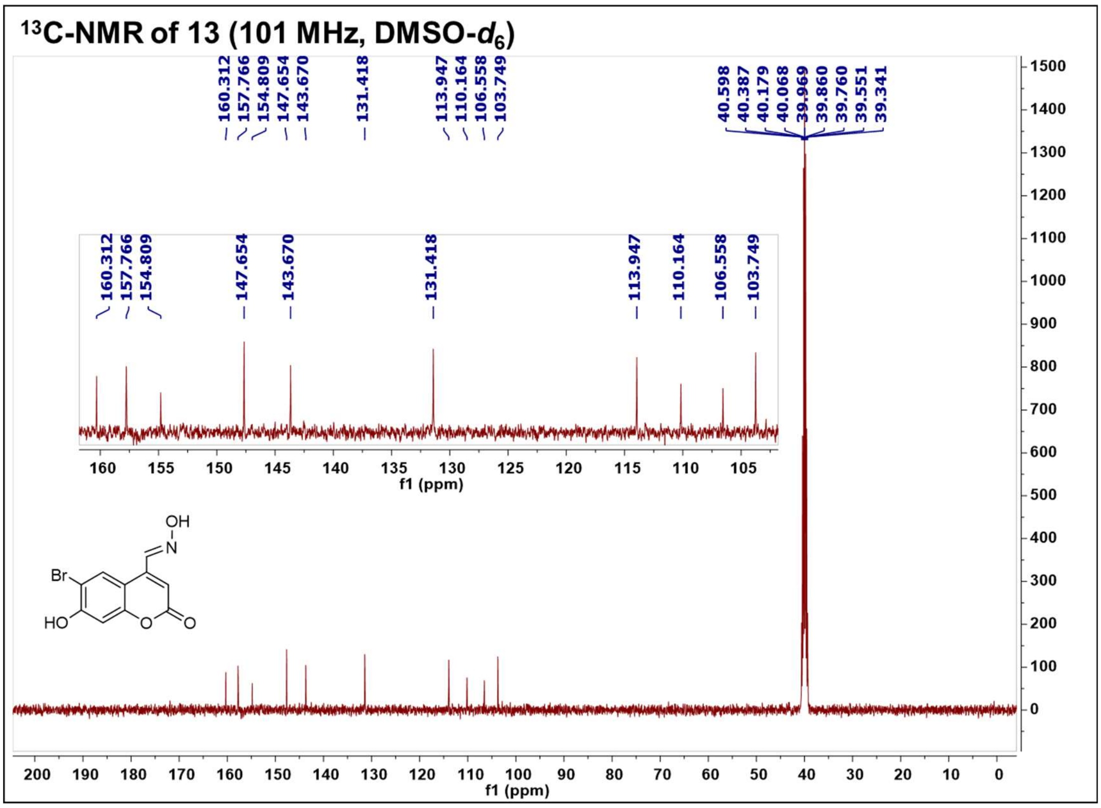

13C-NMR of 13 (101 MHz, DMSO-d6)
160.312
157.766
154.809
147.654
143.670
131.418
113.947
110.164
106.558
103.749
40.598
40.387
40.179
40.068
39.969
39.860
39.760
39.551
39.341
f1 (ppm)
OH
N
Br
HO
O
O

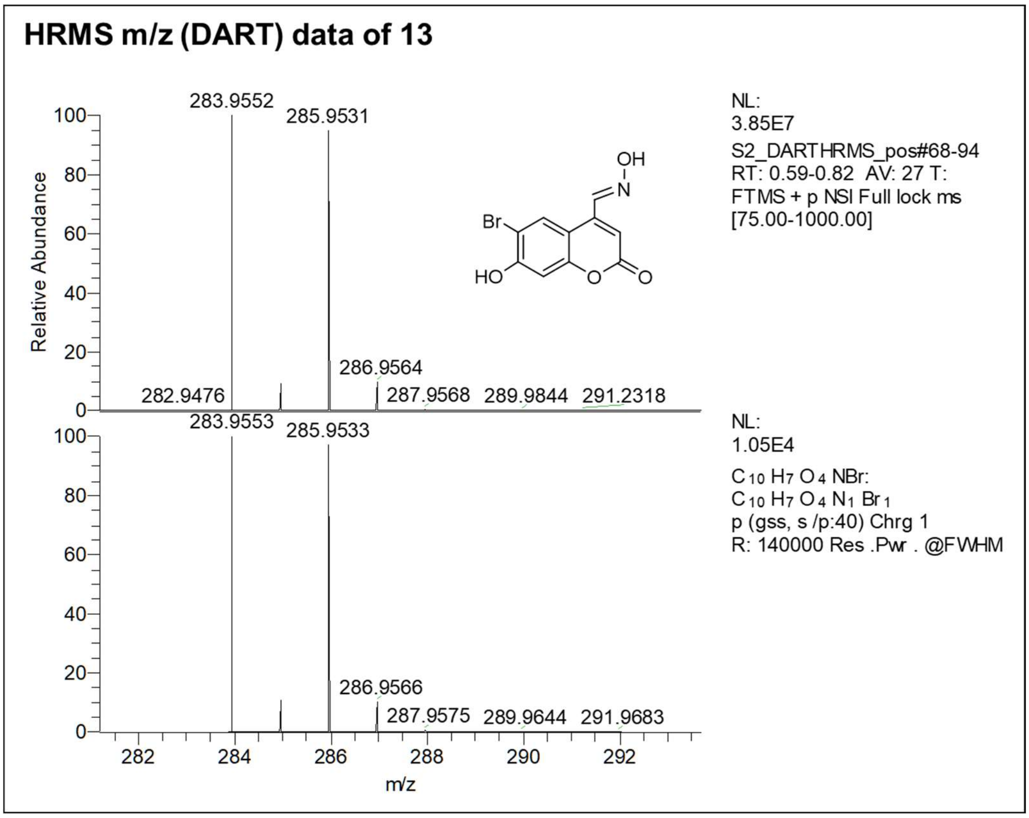

HRMS m/z (DART) data of 13
283.9552
285.9531
286.9564
282.9476
287.9568
289.9844
291.2318
283.9553
285.9533
286.9566
287.9575
289.9644
291.9683
Relative Abundance
m/z
NL:
3.85E7
S2_DARTHRMS_pos#68-94
RT: 0.59-0.82 AV: 27 T:
FTMS + p NSI Full lock ms
[75.00-1000.00]
NL:
1.05E4
C 10 H7 O 4 NBr:
C 10 H7 O 4 N1 Br 1
p (gss, s /p:40) Chrg 1
R: 140000 Res .Pwr . @FWHM
OH
N
Br
HO
O
O
